# Supplementary material for: CRISPR Screens Uncover Genes that Regulate Target Cell Sensitivity to the Morphogen Sonic Hedgehog
Source: Dev Cell. 2018 Jan 8;44(1):113–129.e8. doi: 10.1016/j.devcel.2017.12.003 (PMC5792066; doi:10.1016/j.devcel.2017.12.003)
Supplement: Document S1. Figures S1–S7 [file mmc1.pdf]

Developmental Cell, Volume *44*

Supplemental Information

CRISPR Screens Uncover Genes that Regulate Target Cell Sensitivity to the Morphogen Sonic Hedgehog

Ganesh V. Pusapati, Jennifer H. Kong, Bhaven B. Patel, Arunkumar Krishnan, Andreas Sagner, Maia Kinnebrew, James Briscoe, L. Aravind, and Rajat Rohatgi

Figure S1

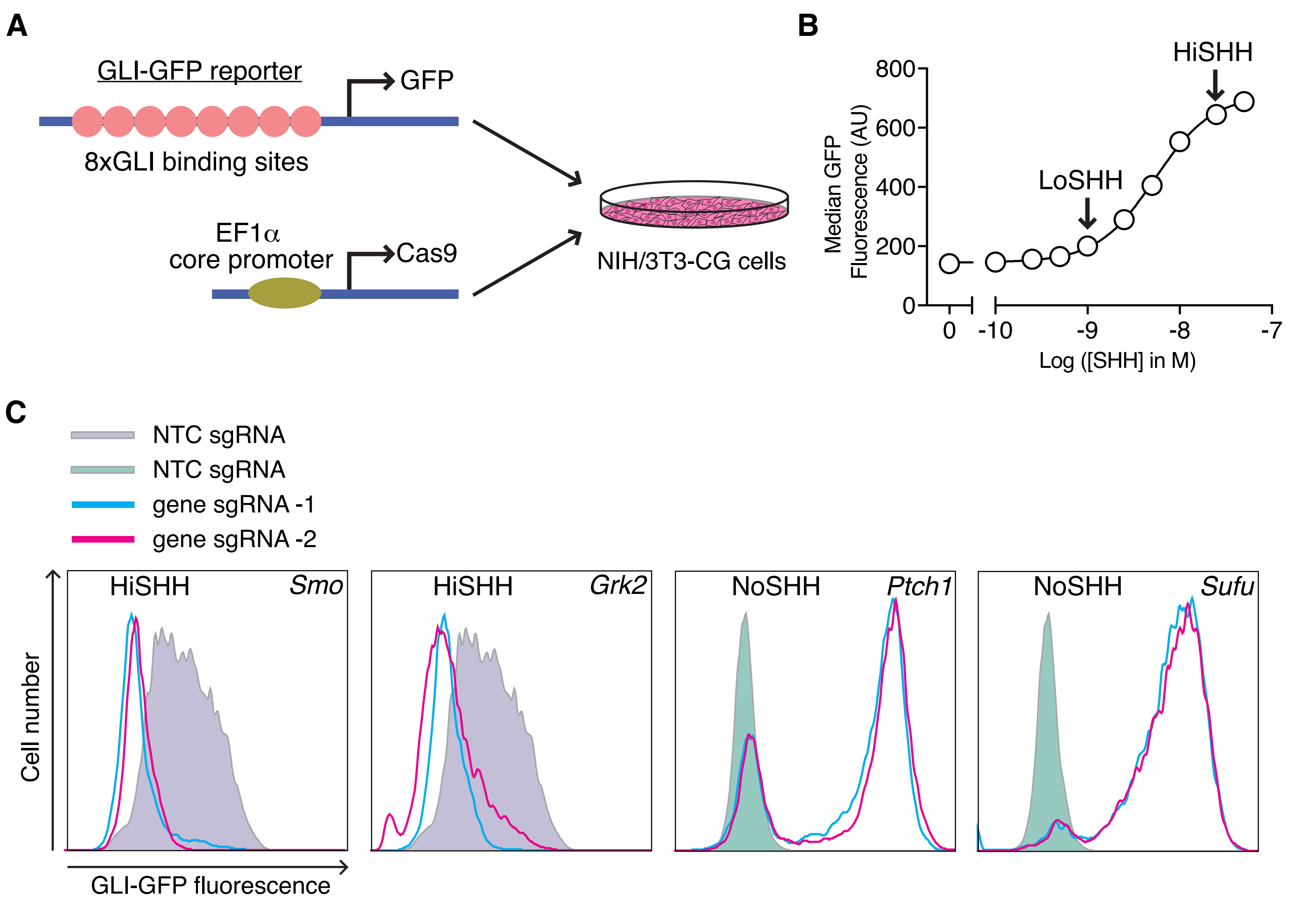
A
GLI-GFP reporter
GFP
8xGLI binding sites
EF1α
core promoter
Cas9
NIH/3T3-CG cells
B
HiSHH
LoSHH
Median GFP Fluorescence (AU)
800
600
400
200
0
0
-10
-9
-8
-7
Log ([SHH] in M)
C
NTC sgRNA
NTC sgRNA
gene sgRNA -1
gene sgRNA -2
HiSHH
Smo
HiSHH
Grk2
NoSHH
Ptch1
NoSHH
Sufu
Cell number
GLI-GFP fluorescence

Figure S1. Related to Figure 1: **Characterization of the NIH/3T3-CG reporter line used in the screens.** (A) NIH/3T3-CG is a clonal derivative of NIH/3T3 cells transduced with a GFP gene driven by a synthetic Hh-responsive promoter composed of eight consecutive binding sites for the GLI transcription factors (GLI-GFP reporter) and a Cas9 gene driven by an EF1α core promoter. (B) SHH induces a dose-dependent increase in the median GFP fluorescence from ~10,000 NIH/3T3-CG cells. Arrows indicate the points on the dose-response curve that correspond to the LoSHH and HiSHH concentrations used throughout the text. (C) Flow cytometry histograms showing the distribution of GFP fluorescence in ~10,000 NIH/3T3-CG cells after transduction with two independent sgRNAs against known positive (*Smo*, *Grk2*) and negative (*Sufu*, *Ptch1*) regulators or one non-targeting control (NTC) sgRNA, followed by treatment with the indicated doses of SHH.

Figure S2

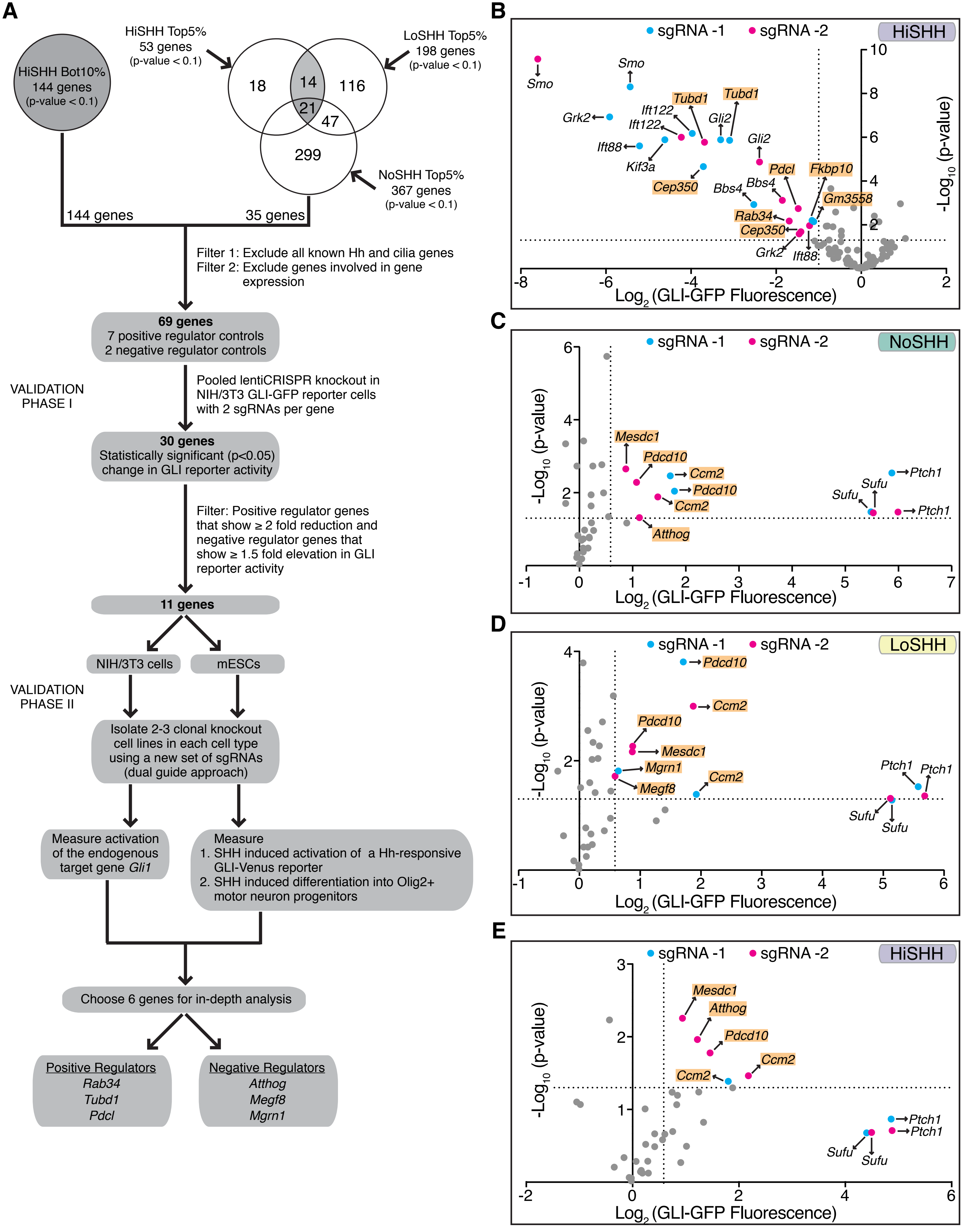
A
HiSHH Bot10% 144 genes (p-value < 0.1)
HiSHH Top5% 53 genes (p-value < 0.1)
LoSHH Top5% 198 genes (p-value < 0.1)
NoSHH Top5% 367 genes (p-value < 0.1)
18
14
116
21
47
299
144 genes
35 genes
Filter 1: Exclude all known Hh and cilia genes
Filter 2: Exclude genes involved in gene expression
69 genes
7 positive regulator controls
2 negative regulator controls
VALIDATION PHASE I
Pooled lentiCRISPR knockout in NIH/3T3 GLI-GFP reporter cells with 2 sgRNAs per gene
30 genes
Statistically significant (p<0.05) change in GLI reporter activity
Filter: Positive regulator genes that show ≥ 2 fold reduction and negative regulator genes that show ≥ 1.5 fold elevation in GLI reporter activity
11 genes
NIH/3T3 cells
mESCs
VALIDATION PHASE II
Isolate 2-3 clonal knockout cell lines in each cell type using a new set of sgRNAs (dual guide approach)
Measure activation of the endogenous target gene Gli1
Measure
1. SHH induced activation of a Hh-responsive GLI-Venus reporter
2. SHH induced differentiation into Olig2+ motor neuron progenitors
Choose 6 genes for in-depth analysis
Positive Regulators
Rab34
Tubd1
Pdcl
Negative Regulators
Atthog
Megf8
Mgrn1
B
sgRNA -1
sgRNA -2
HiSHH
Smo
Grk2
Ift122
Tubd1
Gli2
Ift88
Kif3a
Cep350
Pdcl
Fkbp10
Bbs4
Gm3558
Rab34
Ift88
-Log10 (p-value)
Log2 (GLI-GFP Fluorescence)
C
sgRNA -1
sgRNA -2
NoSHH
Mesdc1
Pdcd10
Ccm2
Ptch1
Sufu
Atthog
-Log10 (p-value)
Log2 (GLI-GFP Fluorescence)
D
sgRNA -1
sgRNA -2
LoSHH
Pdcd10
Ccm2
Mesdc1
Mgrn1
Megf8
Ptch1
Sufu
-Log10 (p-value)
Log2 (GLI-GFP Fluorescence)
E
sgRNA -1
sgRNA -2
HiSHH
Mesdc1
Atthog
Pdcd10
Ccm2
Ptch1
Sufu
-Log10 (p-value)
Log2 (GLI-GFP Fluorescence)

Figure S2. Related to Figure 3: Flow chart for the validation strategy for top hits from all four genome-wide screens and validation of candidate genes by gene-disruption using individually cloned sgRNAs. (A) Flowchart showing our validation pipeline. Genes from the HiSHH_Bot10% screen (*n*=144) enriched with an FDR-corrected p-value<0.1 (shaded circle at top left) and genes (*n*=35) that were enriched in both the HiSHH_Top5% and the LoSHH_Top5% screens with an FDR-corrected p-value<0.1 (shaded section of the Venn diagram at the top right) were passed through two filters, yielding 69 genes that entered Phase I of validation. In Phase I, the top two sgRNAs from the Brie library targeting each of these 69 genes (a total of 138 sgRNAs) were individually expressed in NIH/3T3-CG cells using a lentiviral vector and tested for effects on the GLI-GFP reporter after exposure to NoSHH, HiSHH, or LoSHH (for negative regulators only; B-E). For Phase II of validation, eleven genes (10 in mESCs) were selected for clonal knockout analysis in NIH/3T3 cells and NPCs derived from mESCs (see Figure 3). Finally, 6 genes were chosen for in-depth analysis based on their strong effect on the SHH-induced Hh responses in both NIH/3T3s and NPCs. (B) Fold-activation of the GLI-GFP reporter in pooled NIH/3T3 GLI-GFP reporter cell lines transduced with two sgRNAs against each of the candidate positive regulators from the HiSHH_Bot10% screen after treatment with HiSHH. The median GFP fluorescence from ~10,000 cells, expressed relative that of a control cell line expressing a non-targeting control (NTC) sgRNA, from three independent experiments was used to calculate the overall mean change in GLI-

GFP fluorescence (x-axis) and a *p*-value (y-axis) using the unpaired student's *t*-test. Analogous experiments with candidate negative regulators were performed under conditions of NoSHH (C), LoSHH (D), or HiSHH (E). Dotted lines denote cut-offs for the fold-change in GLI-GFP fluorescence and *p*-value (<0.05) used to select genes (highlighted in orange on the plots) for Phase II of the validation pipeline. *Smo, Grk2, Ift88*, *Ift122, Kif3a, Gli2,* and *Bbs4* served as controls for positive regulators; *Ptch1* and *Sufu* served as controls for negative regulators.

Figure S3

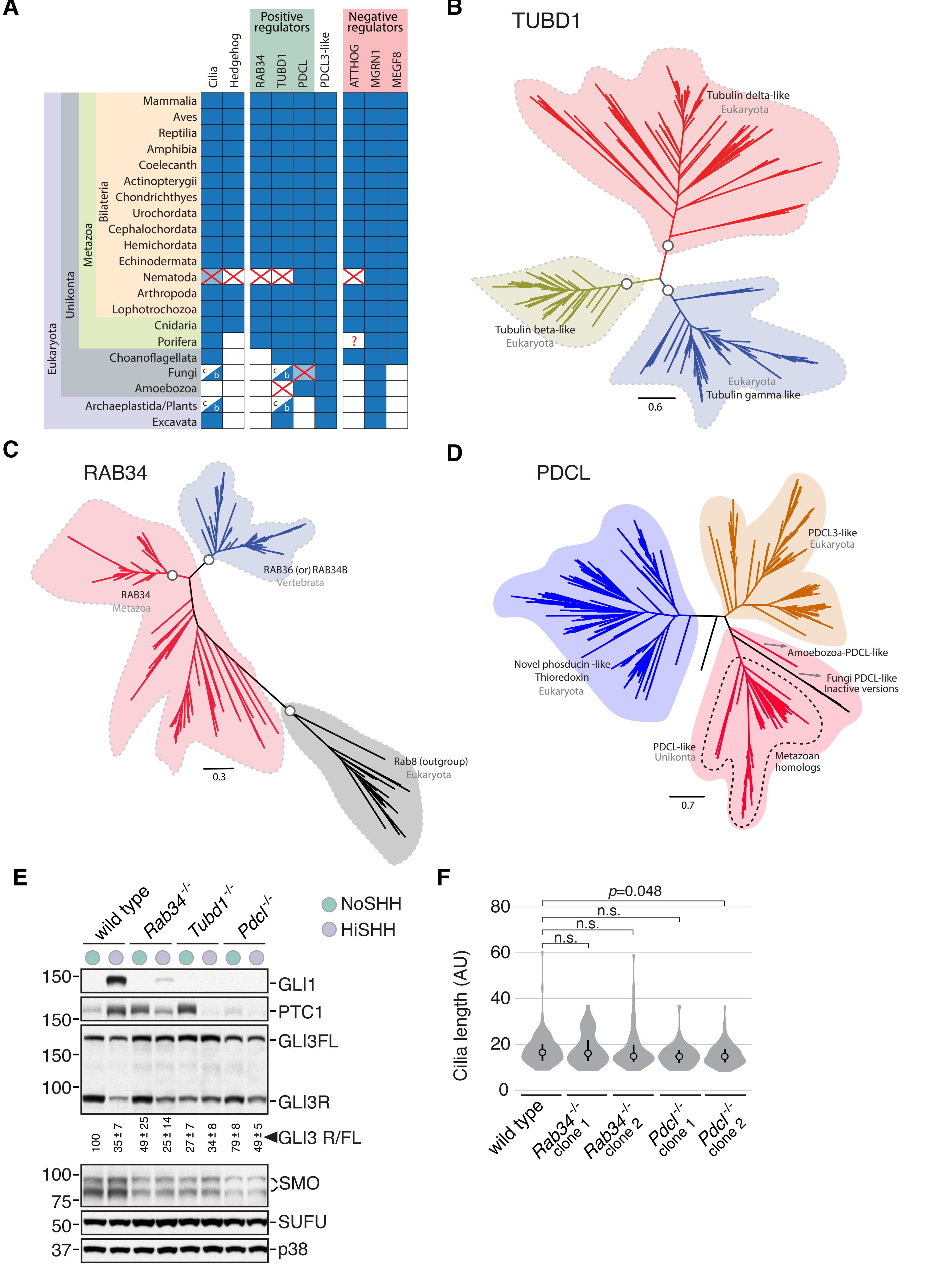
A
Cilia
Hedgehog
Positive regulators
RAB34
TUBD1
PDCL
PDCL3-like
Negative regulators
ATTHOG
MGRN1
MEGF8
Eukaryota
Unikonta
Metazoa
Bilateria
Mammalia
Aves
Reptilia
Amphibia
Coelecanth
Actinopterygii
Chondrichthyes
Urochordata
Cephalochordata
Hemichordata
Echinodermata
Nematoda
Arthropoda
Lophotrochozoa
Cnidaria
Porifera
Choanoflagellata
Fungi
Amoebozoa
Archaeplastida/Plants
Excavata
B
TUBD1
Tubulin delta-like
Eukaryota
Tubulin beta-like
Eukaryota
Eukaryota
Tubulin gamma like
0.6
C
RAB34
RAB36 (or) RAB34B
Vertebrata
RAB34
Metazoa
Rab8 (outgroup)
Eukaryota
0.3
D
PDCL
PDCL3-like
Eukaryota
Amoebozoa-PDCL-like
Fungi PDCL-like
Inactive versions
Novel phosducin -like
Thioredoxin
Eukaryota
PDCL-like
Unikonta
Metazoan
homologs
0.7
E
wild type
Rab34-/-
Tubd1-/-
Pdcl-/-
NoSHH
HiSHH
150
GLI1
PTC1
150
GLI3FL
150
100
GLI3R
100
35 ± 7
49 ± 25
25 ± 14
27 ± 7
34 ± 8
79 ± 8
49 ± 5
GLI3 R/FL
100
75
SMO
50
SUFU
37
p38
F
p=0.048
n.s.
n.s.
n.s.
80
60
40
20
0
Cilia length (AU)
wild type
Rab34-/- clone 1
Rab34-/- clone 2
Pdcl-/- clone 1
Pdcl-/- clone 2

Figure S3. Related to Figure 4: Phylogenetic relationships of the Hh signaling regulators identified by CRISPR-based screens. (A) Phyletic patterns of the positive and negative regulators of Hh signaling from our screens relative to the evolution of cilia and Hh signaling. Blue fill denotes presence, white fill denotes absence prior to origin of the gene/pathway/organelle, a red cross denotes a loss, and a red "?" denotes uncertainty given the insufficiency of whole genome data. Half-filled squares shown for fungi and plants denote the presence in basal lineages but a loss in crown lineages. TUBD1, which localizes to centrioles in metazoans, is lost in nematodes, which have an aberrant centriolar morphology and composition and have lost both motile cilia (faded blue box) and Hh signaling but have retained primary (sensory) cilia. The faded blue box for PDCL indicates loss across most fungi but sporadic retention of divergent versions (see D). (B, C, D) Unrooted tree topologies showing evolutionary relationships of TUBD1, RAB34, and PDCL to their closest related families as identified by iterative sequence profile searches. Divergent families forming monophyletic clades are highlighted in distinct colors within each tree. The evolutionary provenance of each family traced in this study is indicated below the protein name. Filled white circles with black outlines on the nodes denote a support of ≥ 0.9 using the Shimodaira-Hasegawa test on 1000 resamples. Phylogenetic analysis of the phosducin-like clade (A and D) indicates that a single member, prototyped by *Pdcl3,* was ancestrally present in eukaryotes and functioned independently of the presence of heterotrimeric G-protein function.

However*,* *Pdcl* itself is only found in the unikont clade of eukaryotes, including amoebozoans, fungi, and metazoans. Notably, *Pdcl* (unlike *Pdcl3*) is lost or potentially inactive in most fungi and is anomalously fast-evolving in nematodes, both of which lack motile cilia and canonical Hh signaling. (E) Immunoblots were used to measure levels of the indicated Hh pathway proteins in extracts of *Rab34*$^{-/-}$, *Tubd1*$^{-/-}$, or *Pdcl*$^{-/-}$ NIH/3T3 cells. This data is derived from a set of clonal cell lines distinct from that used in Figure 4B. (F) Violin plots showing ciliary length distributions of either wild type NIH/3T3 or two clonal NIH/3T3 cell lines carrying deletions in *Rab34* and *Pdcl*. Statistical significance was determined by the Kruskal-Wallis test and depicted as follows: $p > 0.05$ (ns, not significant).

Figure S4

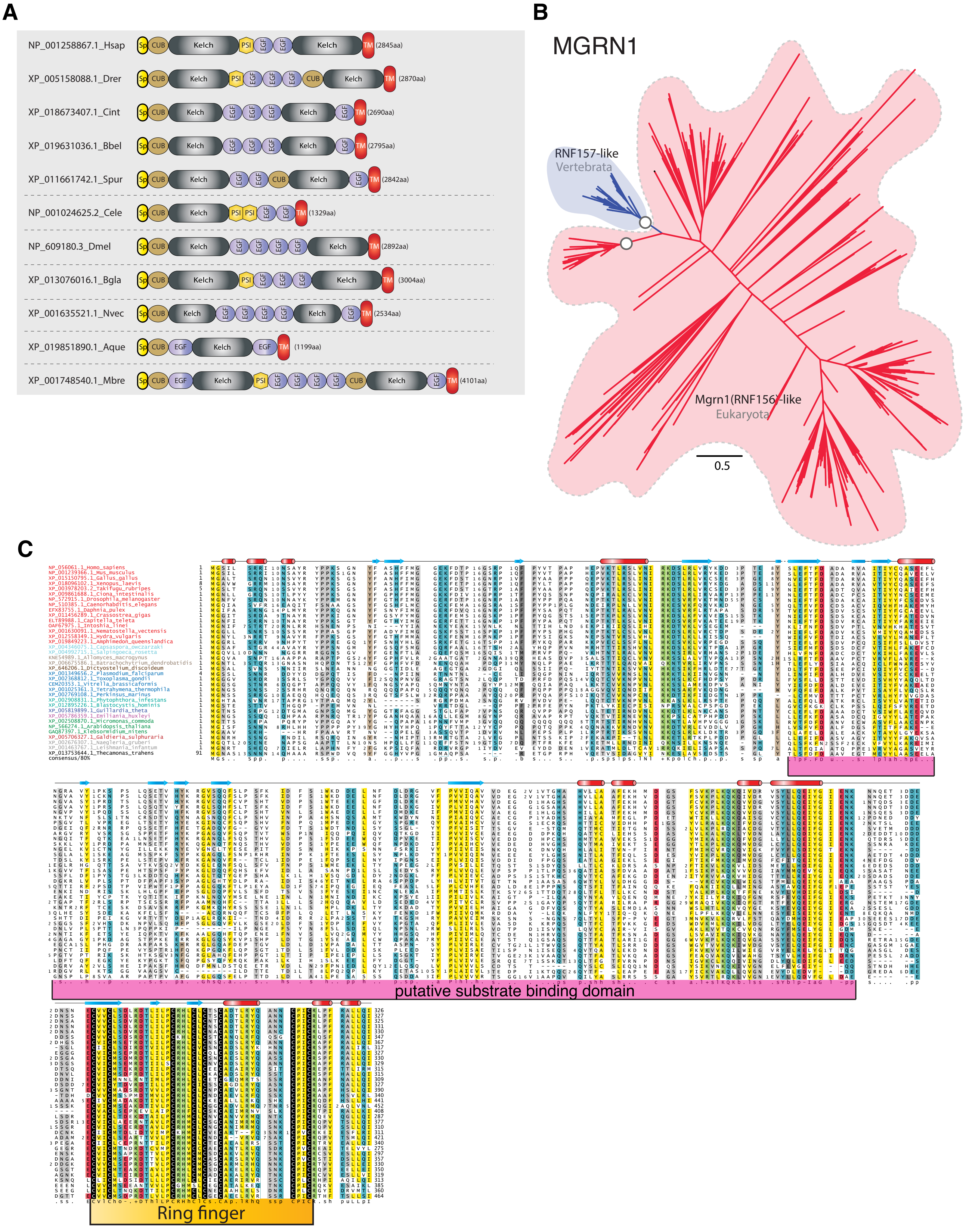
A
NP_001258867.1_Hsap
XP_005158088.1_Drer
XP_018673407.1_Cint
XP_019631036.1_Bbel
XP_011661742.1_Spur
NP_001024625.2_Cele
NP_609180.3_Dmel
XP_013076016.1_Bgla
XP_001635521.1_Nvec
XP_019851890.1_Aque
XP_001748540.1_Mbre
Sp
CUB
Kelch
PSI
EGF
TM
(2845aa)
(2870aa)
(2690aa)
(2795aa)
(2842aa)
(1329aa)
(2892aa)
(3004aa)
(2534aa)
(1199aa)
(4101aa)
B
MGRN1
RNF157-like
Vertebrata
Mgrn1(RNF156)-like
Eukaryota
0.5
C
NP_056061.1_Homo_sapiens
NP_001239366.1_Mus_musculus
XP_015150795.1_Gallus_gallus
XP_018096102.1_Xenopus_laevis
XP_003978203.2_Takifugu_rubripes
XP_009861688.1_Ciona_intestinalis
NP_572915.1_Drosophila_melanogaster
NP_510385.1_Caenorhabditis_elegans
EFX83755.1_Daphnia_pulex
XP_011456289.1_Crassostrea_gigas
ELT89988.1_Capitella_teleta
OAF67975.1_Intoshia_linei
XP_001630091.1_Nematostella_vectensis
XP_012558349.1_Hydra_vulgaris
XP_019849223.1_Amphimedon_queenslandica
XP_004346075.1_Capsaspora_owczarzaki
XP_004992715.1_Salpingoeca_rosetta
KNE54989.1_Allomyces_macrogynus
XP_006675586.1_Batrachochytrium_dendrobatidis
XP_646206.1_Dictyostelium_discoideum
XP_001349656.2_Plasmodium_falciparum
XP_002368812.1_Toxoplasma_gondii
CEM20353.1_Vitrella_brassicaformis
XP_001025361.3_Tetrahymena_thermophila
XP_002769108.1_Perkinsus_marinus
XP_002908831.1_Phytophthora_infestans
XP_012895226.1_Blastocystis_hominis
XP_005819899.1_Guillardia_theta
XP_005786359.1_Emiliania_huxleyi
XP_002508870.1_Micromonas_commoda
NP_566274.1_Arabidopsis_thaliana
GAQ87397.1_Klebsormidium_nitens
XP_005706327.1_Galdieria_sulphuraria
XP_002676307.1_Naegleria_gruberi
XP_001463767.1_Leishmania_infantum
XP_013753644.1_Thecamonas_trahens
consensus/80%
putative substrate binding domain
Ring finger

Figure S4. Related to Figure 5: Sequence analysis of the two Hh attenuators MEGF8 and MGRN1. (A) Domain architectures of selected MEGF8 homologs across all major holozoan lineages. The proteins are represented by GenBank identifiers (GIs) followed by their species abbreviations (see Methods). Domain abbreviations: SP, signal peptide; TM, transmembrane region; CUB (complement C1r/C1s, Uegf Bmp1) domain; Kelch repeats; PSI repeats, cysteine rich repeats found in plexins, semaphorins and integrins; EGF, Epidermal growth factor. (B) Unrooted tree topologies showing evolutionary relationships of MGRN1 to its closest related families, depicted as in Figure S3. (C) Multiple sequence alignment of the E3 ubiquitin-protein ligase MGRN1. Predicted secondary structure is denoted above the alignment. MGRN1 contains a RING domain likely to interact with E2 ubiquitin conjugating enzymes and a previously unannotated but conserved N-terminal domain that is likely to bind substrates, perhaps microtubules. The proteins names are represented by GIs followed by their full species names (see Methods) and are colored per major lineages of holozoans.

Figure S5

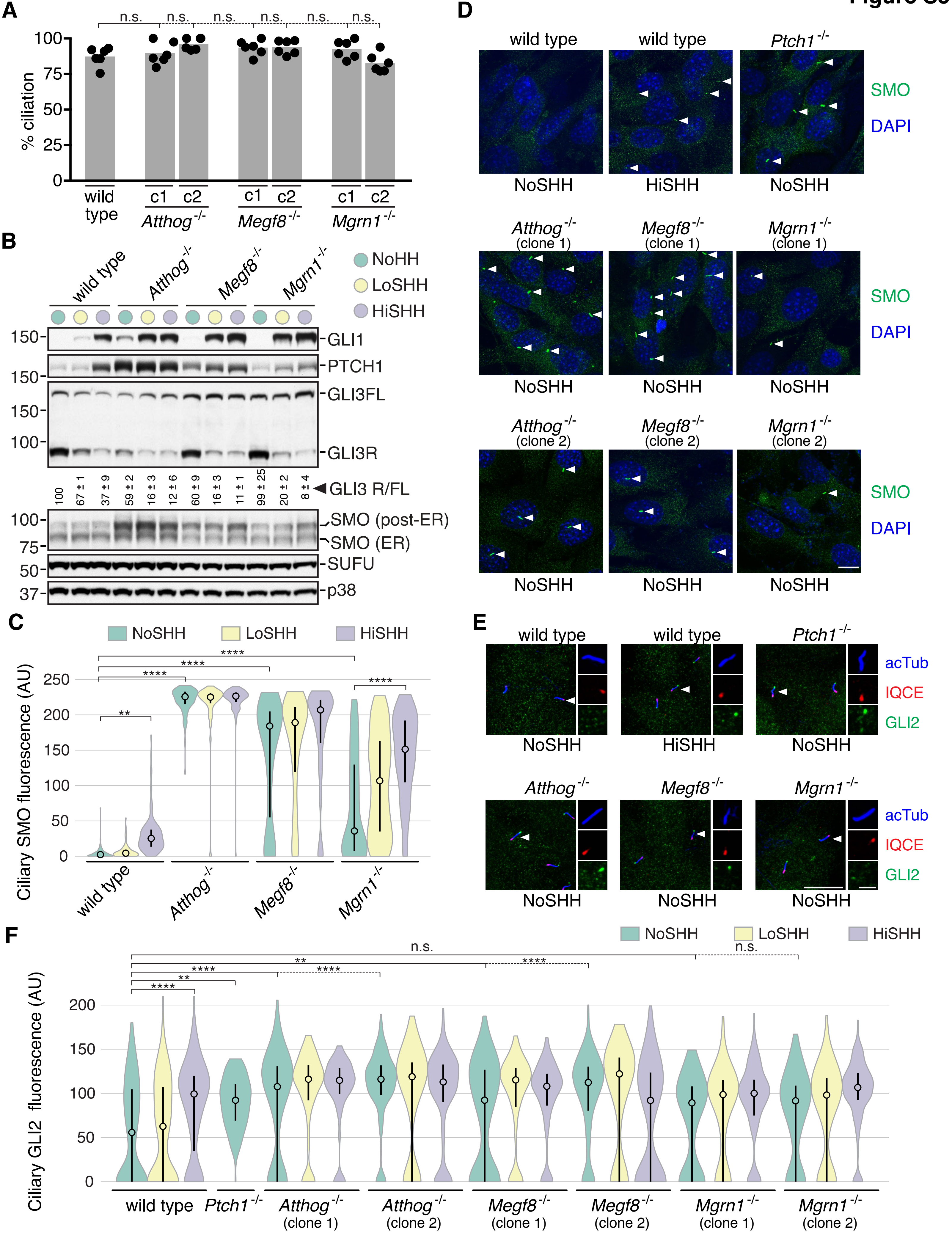
A
n.s.
% ciliation
100
75
50
25
0
wild type
c1
c2
Atthog -/-
Megf8 -/-
Mgrn1 -/-
B
wild type
Atthog -/-
Megf8 -/-
Mgrn1 -/-
NoHH
LoSHH
HiSHH
150
100
75
50
37
GLI1
PTCH1
GLI3FL
GLI3R
100
67 ± 1
37 ± 9
59 ± 2
16 ± 3
12 ± 6
60 ± 9
16 ± 3
11 ± 1
99 ± 25
20 ± 2
8 ± 4
GLI3 R/FL
SMO (post-ER)
SMO (ER)
SUFU
p38
C
NoSHH
LoSHH
HiSHH
Ciliary SMO fluorescence (AU)
****
**
D
wild type
Ptch1 -/-
SMO
DAPI
NoSHH
HiSHH
(clone 1)
(clone 2)
E
acTub
IQCE
GLI2
F
Ciliary GLI2 fluorescence (AU)
n.s.
****
**

Figure S5. Related to Figure 5: The deletion of *Atthog, Megf8,* and *Mgrn1* increases ciliary levels of SMO and GLI2. (A) Frequency of ciliation in wild type NIH/3T3 cells and two independent clonal *Atthog*$^{-/-}$, *Megf8*$^{-/-}$, and *Mgrn1*$^{-/-}$ cell lines. Bars represent the mean ciliation frequency derived from six separate fields, each shown by a black dot (n~200 cells). (B) Immunoblots were used to measure levels of the indicated Hh pathway proteins in extracts of *Atthog*$^{-/-}$, *Megf8*$^{-/-}$, and *Mgrn1*$^{-/-}$ cells treated with LoSHH or HiSHH. Analogous data from an independent set of clonal cell lines is shown in Figure 5A. (C) Violin plots showing fluorescence intensity distributions of SMO in primary cilia (n=100 cilia) of the indicated NIH/3T3 cell lines. Analogous data from an independent set of clonal cell lines is shown in Figure 5D. (D) Cell-surface SMO was detected by immunofluorescence in intact (non-permeabilized) NIH/3T3 cell lines using an antibody against an epitope on the extracellular region of SMO. Arrowheads show surface SMO enriched in primary cilia. (E and F) Representative micrographs (E) and corresponding violin plots (F) showing the accumulation of endogenous GLI2 at the tips of primary cilia in clonal NIH/3T3 cell lines. AcTub staining (blue) extends along the entire length of the ciliary axoneme, IQCE staining (red) marks the base of cilia and GLI2 staining (green) is seen at the tip of cilia, at the opposite end of the axoneme from IQCE staining. Statistical significance was determined by the Kruskal-Wallis test ($p < 0.01$ (**), $p < 0.0001$ (****), and $p > 0.05$ (ns)). Scale bars, 10 μm in merged panels and 2 μm in zoomed displays.

Figure S6

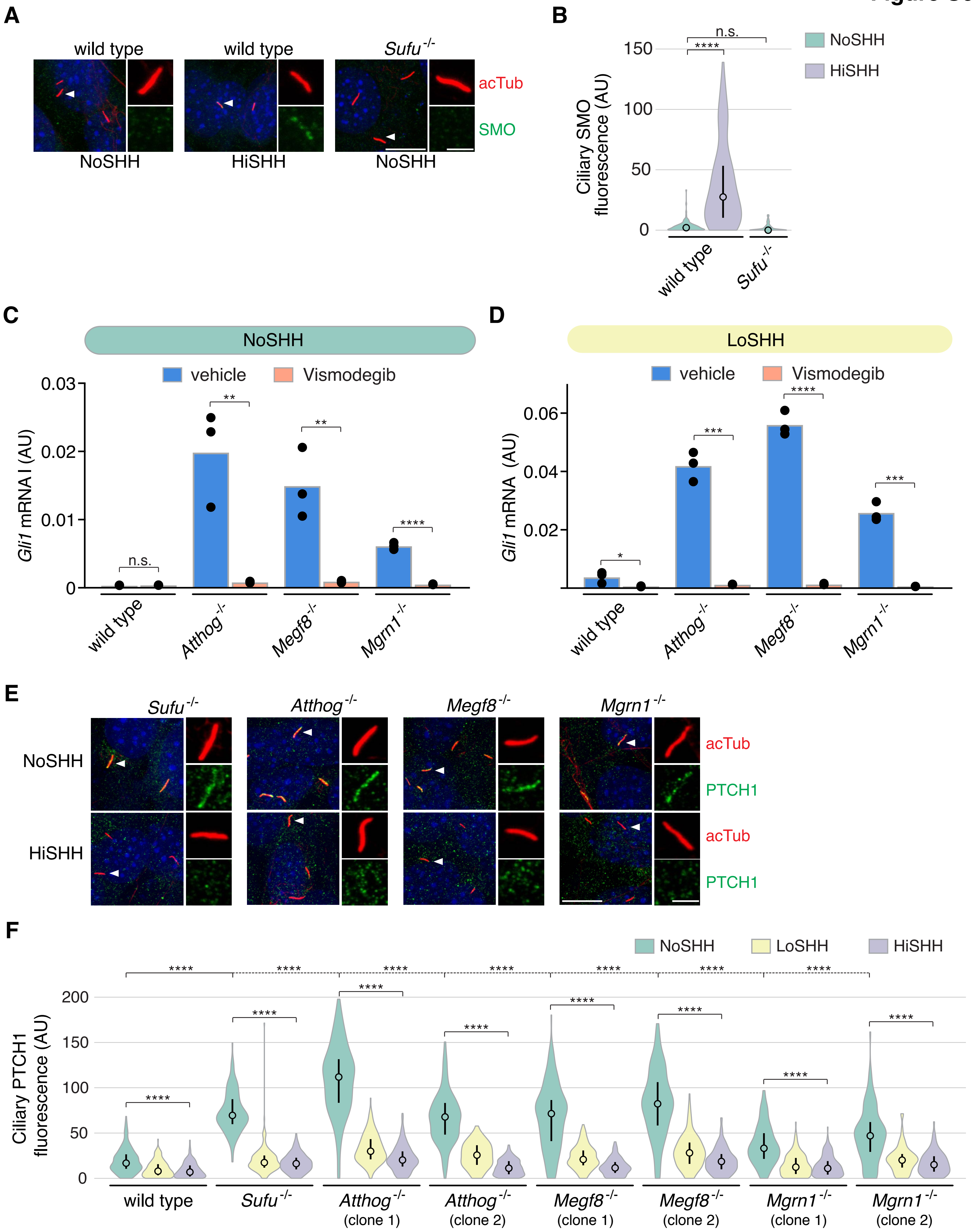
A
wild type
wild type
Sufu -/-
acTub
SMO
NoSHH
HiSHH
NoSHH
B
n.s.
****
NoSHH
HiSHH
Ciliary SMO fluorescence (AU)
150
100
50
0
wild type
Sufu -/-
C
NoSHH
vehicle
Vismodegib
Gli1 mRNA l (AU)
0.03
0.02
0.01
0
n.s.
**
**
****
wild type
Atthog -/-
Megf8 -/-
Mgrn1 -/-
D
LoSHH
vehicle
Vismodegib
Gli1 mRNA (AU)
0.06
0.04
0.02
*
***
****
***
wild type
Atthog -/-
Megf8 -/-
Mgrn1 -/-
E
Sufu -/-
Atthog -/-
Megf8 -/-
Mgrn1 -/-
NoSHH
HiSHH
acTub
PTCH1
acTub
PTCH1
F
NoSHH
LoSHH
HiSHH
Ciliary PTCH1 fluorescence (AU)
200
150
100
50
0
****
wild type
Sufu -/-
Atthog -/- (clone 1)
Atthog -/- (clone 2)
Megf8 -/- (clone 1)
Megf8 -/- (clone 2)
Mgrn1 -/- (clone 1)
Mgrn1 -/- (clone 2)

Figure S6. Related to Figure 5: ATTHOG, MEGF8, and MGRN1 influence the ciliary trafficking of SMO, but not of PTCH1. (A and B) Representative micrographs (A) and corresponding violin plots (B, n=100 cilia per condition) showing the abundance of endogenous SMO in primary cilia of wild type NIH/3T3 cells and *Sufu*$^{-/-}$ cells left untreated or treated with HiSHH. (C and D) Vismodegib blocks both basal (C) and LoSHH-induced (D) *Gli1* mRNA levels in *Atthog*$^{-/-}$, *Megf8*$^{-/-}$, and *Mgrn1*$^{-/-}$ cell lines. Analogous data from an independent set of clonal cell lines is shown in Figures 5E and 5F. Bars depict the mean *Gli1* mRNA level derived from three independent replicates, each denoted by a black dot. Statistical significance was determined by the unpaired student's *t*-test ($p < 0.05$ (*), $p < 0.01$ (**), $p < 0.001$ (***), $p < 0.0001$ (****), and $p > 0.05$ (ns)). (E and F) Representative micrographs (E) and corresponding violin plots (F, n=100 cilia per condition) showing levels of endogenous PTCH1 in primary cilia of NIH/3T3 cell lines left untreated or treated with the indicated doses of SHH. Statistical significance in (B) and (F) was determined by the Kruskal-Wallis test and depicted as $p < 0.0001$ (****) and $p > 0.05$ (ns). Scale bars, 10 μm in merged panels and 2 μm in zoomed displays.

Figure S7

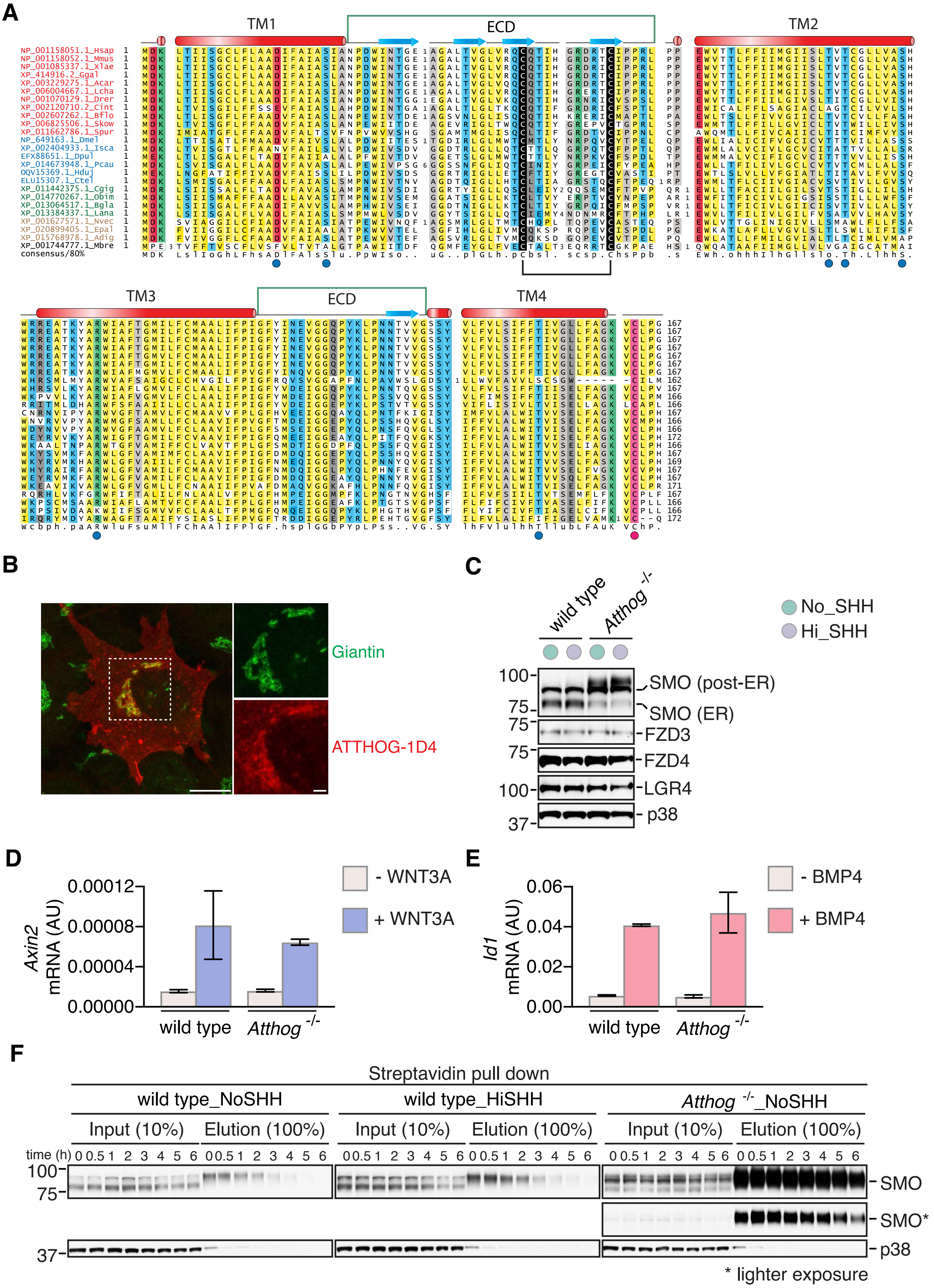

A
TM1
ECD
TM2
TM3
ECD
TM4
NP_001158051.1_Hsap
NP_001158052.1_Mmus
NP_001085337.1_Xlae
XP_414916.2_Ggal
XP_003229275.1_Acar
XP_006004667.1_Lcha
NP_001070129.1_Drer
XP_002120710.2_Cint
XP_002607262.1_Bflo
XP_006825506.1_Skow
XP_011662786.1_Spur
NP_649163.1_Dmel
XP_002404933.1_Isca
EFX88651.1_Dpul
XP_014673948.1_Pcau
OQV15369.1_Hduj
ELU15307.1_Ctel
XP_011442375.1_Cgig
XP_014770267.1_Obim
XP_013064517.1_Bgla
XP_013384337.1_Lana
XP_001627571.1_Nvec
XP_020899405.1_Epal
XP_015768978.1_Adig
XP_001744777.1_Mbre
consensus/80%
B
Giantin
ATTHOG-1D4
C
wild type
Atthog -/-
No_SHH
Hi_SHH
100
75
75
75
100
37
SMO (post-ER)
SMO (ER)
FZD3
FZD4
LGR4
p38
D
0.00012
0.00008
0.00004
0.00000
Axin2 mRNA (AU)
- WNT3A
+ WNT3A
wild type
Atthog -/-
E
0.06
0.04
0.02
0.00
Id1 mRNA (AU)
- BMP4
+ BMP4
wild type
Atthog -/-
F
Streptavidin pull down
wild type_NoSHH
wild type_HiSHH
Atthog -/-_NoSHH
Input (10%)
Elution (100%)
time (h) 0 0.5 1 2 3 4 5 6
100
75
37
SMO
SMO*
p38
* lighter exposure

Figure S7. Related to Figures 6 and 7: Characterization of ATTHOG function in Hedgehog signaling. (A) Sequence alignment of members of the ATTHOG family of claudin-like tetraspanin proteins. The proteins are named by their GenBank identifiers (GIs), followed by their species abbreviations (see Methods) and colored per major lineages of holozoans. Predicted secondary structure is shown on the top, including the putative disulfide bridge in black. Conserved polar and charged residues that spatially cluster within the TM bundle (Figure 6B) are marked with blue circles at the bottom. The terminal cysteine predicted to be palmitoylated is highlighted with a pink circle. The alignment is colored based on 80% consensus with the following scheme: h (hydrophobic), l (aliphatic), and a (aromatic) are shaded yellow; p (polar) are shaded blue; positively charged and negatively charged residues are shaded green and red respectively; s (small) and t (tiny) are shaded light grey; b (big) is shaded dark gray. (B) ATTHOG staining in NIH/3T3 cells overlaps with staining of a marker of a Golgi marker (Giantin). (C) Immunoblotting was used to assess the abundance of SMO or three other members of the GPCR family (FZD3, FZD4, and LGR4) in wild type and *Atthog*$^{-/-}$ cells. (D and E) Endogenous transcriptional responses to WNT3A (D) and BMP4 (E) were measured in wild type and *Atthog*$^{-/-}$ NIH/3T3 cells using qRT-PCR for *Axin2* (target gene for WNT3A) and *Id1* (target gene for BMP4) (F) Immunoblotting was used to measure the abundance of biotinylated SMO at various times after labeling the SMO pool present at the plasma

membrane using a cell-impermeable biotinylation reagent (see methods). Scale bars, 10 μm in merged panels and 2 μm in zoomed displays.
