# Supplementary material for: CRISPR Screens Uncover Genes that Regulate Target Cell Sensitivity to the Morphogen Sonic Hedgehog
Source: Dev Cell. 2018 Jan 8;44(1):113–129.e8. doi: 10.1016/j.devcel.2017.12.003 (PMC5792066; doi:10.1016/j.devcel.2017.12.003)
Supplement: Document S2. Article plus Supplemental Information [file mmc5.pdf]

Resource

Developmental Cell

CRISPR Screens Uncover Genes that Regulate Target Cell Sensitivity to the Morphogen Sonic Hedgehog

Graphical Abstract

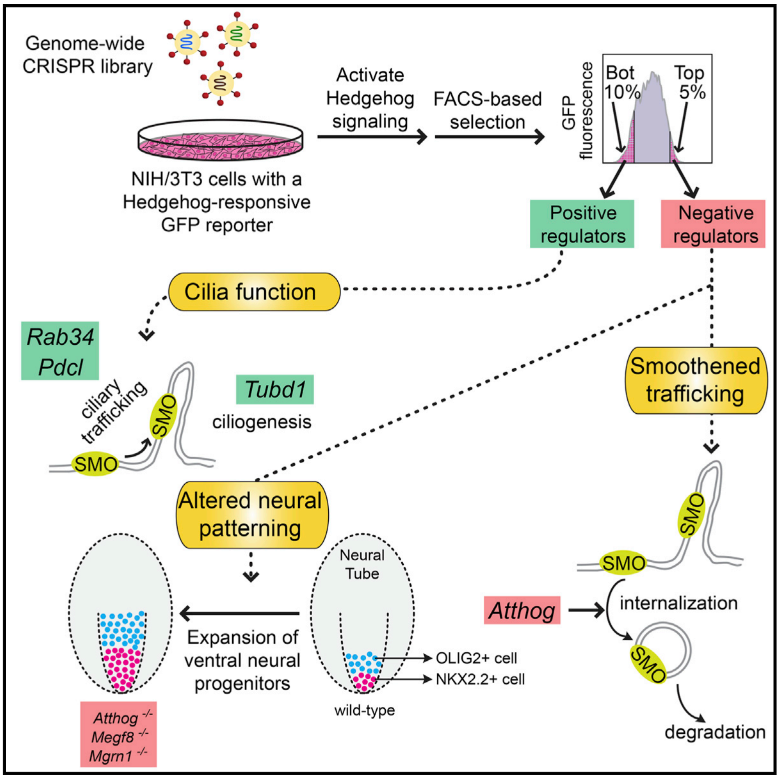

Highlights

- CRISPR screens identify positive and negative regulators of Hedgehog signaling
- Screens identified known and new genes linked to cilia and ciliopathies
- *Atthog*, *Megf8*, and *Mgrn1* attenuate target cell responses to Hedgehog signals
- Attenuators of signaling promote the endocytosis and degradation of Smoothened

Authors

Ganesh V. Pusapati, Jennifer H. Kong, Bhaven B. Patel, ..., James Briscoe, L. Aravind, Rajat Rohatgi

Correspondence

ganesh22@stanford.edu (G.V.P.), rrohatgi@stanford.edu (R.R.)

In Brief

Pusapati et al. use CRISPR-based screens in mammalian cells to identify genes that modify cellular responses to Hedgehog morphogens. The screens provide a comprehensive view of the regulatory structure of Hedgehog signaling, highlight its intimate connection to primary cilia and ciliopathies, and uncover a role for Smoothened trafficking in regulating signaling strength.

Pusapati et al., 2018, Developmental Cell *44*, 113–129
January 8, 2018 © 2018 The Authors. Published by Elsevier Inc.
https://doi.org/10.1016/j.devcel.2017.12.003

CellPress

CRISPR Screens Uncover Genes that Regulate Target Cell Sensitivity to the Morphogen Sonic Hedgehog

Ganesh V. Pusapati,[1,4,*] Jennifer H. Kong,[1,4] Bhaven B. Patel,[1,4] Arunkumar Krishnan,[2] Andreas Sagner,[3] Maia Kinnebrew,[1] James Briscoe,[3] L. Aravind,[2] and Rajat Rohatgi[1,5,*]
[1]Departments of Medicine and Biochemistry, Stanford University School of Medicine, Stanford, CA 94305, USA
[2]National Center for Biotechnology Information, National Library of Medicine, National Institutes of Health, Bethesda, MD 20894, USA
[3]The Francis Crick Institute, Midland Road, London NW1 1AT, UK
[4]These authors contributed equally
[5]Lead Contact
*Correspondence: ganesh22@stanford.edu (G.V.P.), rrohatgi@stanford.edu (R.R.)
https://doi.org/10.1016/j.devcel.2017.12.003

SUMMARY

To uncover regulatory mechanisms in Hedgehog (Hh) signaling, we conducted genome-wide screens to identify positive and negative pathway components and validated top hits using multiple signaling and differentiation assays in two different cell types. Most positive regulators identified in our screens, including *Rab34*, *Pdcl*, and *Tubd1*, were involved in ciliary functions, confirming the central role for primary cilia in Hh signaling. Negative regulators identified included *Megf8*, *Mgrn1*, and an unannotated gene encoding a tetraspan protein we named *Atthog*. The function of these negative regulators converged on Smoothened (SMO), an oncoprotein that transduces the Hh signal across the membrane. In the absence of *Atthog*, SMO was stabilized at the cell surface and concentrated in the ciliary membrane, boosting cell sensitivity to the ligand Sonic Hedgehog (SHH) and consequently altering SHH-guided neural cell-fate decisions. Thus, we uncovered genes that modify the interpretation of morphogen signals by regulating protein-trafficking events in target cells.

INTRODUCTION

The Hedgehog (Hh) signaling pathway is a system for cell-cell communication that coordinates multiple processes during animal development. A hallmark of Hh ligands is their function as morphogens, secreted factors that pattern tissues as diverse as the *Drosophila* wing disc and the vertebrate spinal cord. The mechanism by which Hh ligands inscribe a pattern on a population of precursor cells is based on their ability to guide the adoption of distinct cell fates in response to different levels of signaling. For example, in the vertebrate neural tube, a temporal and spatial gradient of the ligand Sonic Hedgehog (SHH) drives the patterning of spinal neural progenitor subtypes along the dorsal-ventral axis (Dessaud et al., 2008).

Genetics has played a central role in the discovery and mechanistic understanding of Hh signaling. Both the identities and regulatory relationships between many of the protein components in the Hh pathway were elucidated initially through genetic analyses in *Drosophila* (Nüsslein-Volhard and Wieschaus, 1980). Two decades later, forward genetic screens in the mouse led to the surprising discovery that vertebrate (but not *Drosophila*) Hh signaling depends on primary cilia, solitary membrane-enveloped projections present on the surfaces of most cells (Huangfu et al., 2003). These specialized organelles function as signaling centers during development in many tissues, demonstrated by the discovery of an ever-expanding class of human genetic disorders called ciliopathies (Reiter and Leroux, 2017). Many of the phenotypes seen in patients with ciliopathies are consistent with abnormalities in Hh signaling (Bangs and Anderson, 2016). Thus, genetic screens performed in different systems and under a variety of conditions can uncover unexpected layers of regulation in signaling pathways.

Recent methodological advances using CRISPR/Cas9-based methods or haploid human cells have facilitated the application of genome-wide, loss-of-function screens to probe signaling pathways in cultured cells (Lebensohn et al., 2016; Parnas et al., 2015). We systematically screened for positive, negative, and attenuating regulators of Hh signal reception using a fluorescence-based transcriptional reporter for phenotypic enrichment. In a set of four genome-wide screens, we identified most of the core, non-redundant components of vertebrate Hh signaling. Consistent with the body of work from human and mouse genetics, these screens confirmed the importance of cilia in Hh signaling, detecting ∼20% of known cilia genes and ∼30% of known ciliopathy genes as having a significant effect on Hh signaling. Our screens for negative and attenuating regulators uncovered a role for membrane trafficking events in modifying target cell responses to Hh ligands. Loss-of-function mutations in three of the principal hits sensitized cultured fibroblasts and neural progenitor cells to SHH, shifting the SHH dose-response curve and altering the relationship between the concentration of SHH and target cell fate. The combined results of these screens provide a comprehensive view of the regulatory structure of Hh signaling and its intimate connection to primary cilia and ciliopathies.

RESULTS

Genetic Screens in Mouse Fibroblasts to Identify Regulators of the Hh Pathway

We constructed and characterized a clonal NIH/3T3 cell line, hereafter called NIH/3T3-CG, that expressed (1) Cas9 and

This is an open access article under the CC BY license (http://creativecommons.org/licenses/by/4.0/).

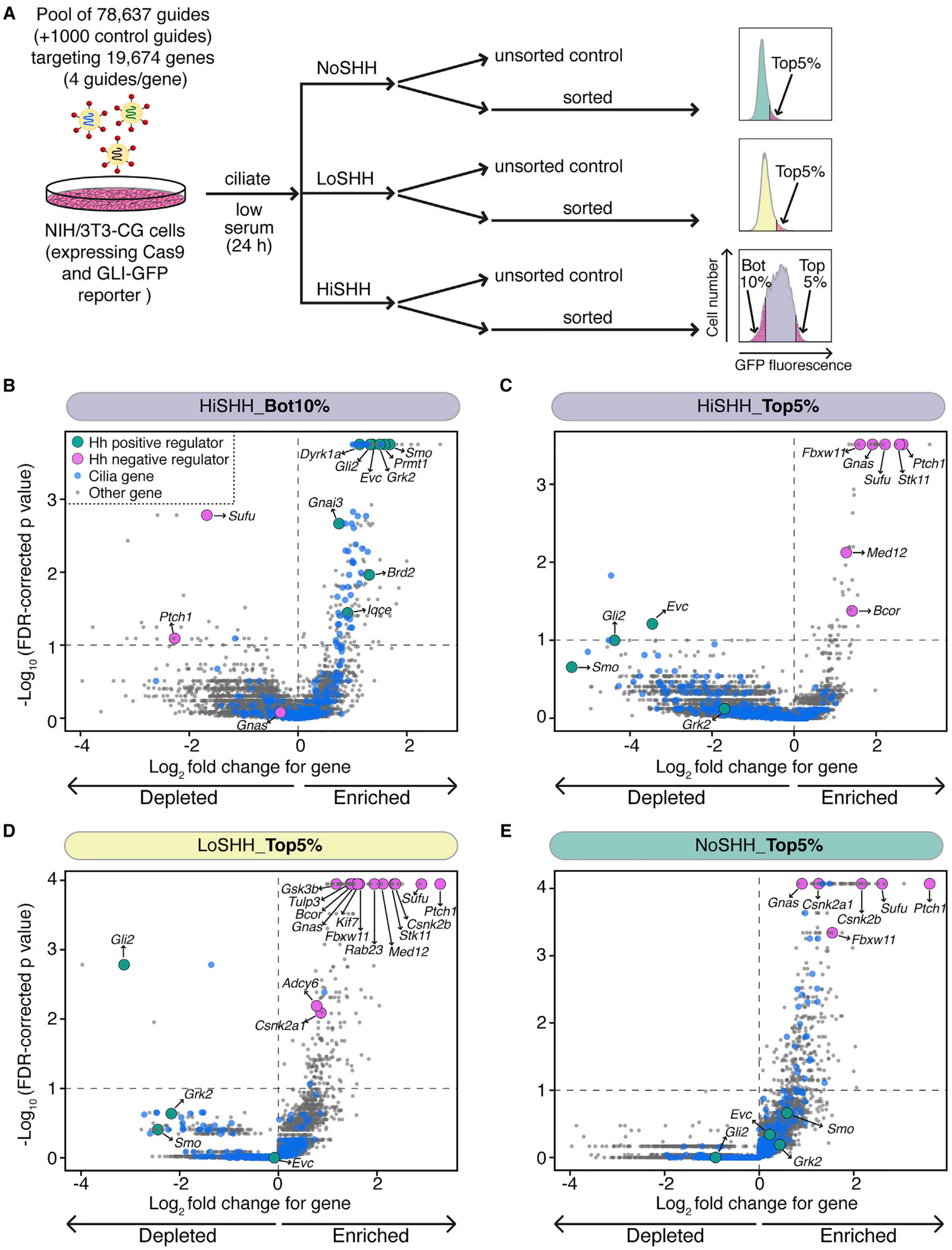

(legend on next page)

(2) GFP driven by a Hh-responsive promoter element containing eight binding sites for the GLI family of Hh transcription factors (GLI-GFP reporter) (Figure S1A). GFP fluorescence in NIH/3T3-CG cells increased in response to SHH in a dose-dependent, saturable fashion (Figure S1B). For the screens and the follow-up experiments described in this study, we left cells untreated (hereafter labeled "NoSHH"), exposed them to a low, sub-saturating concentration of SHH ("LoSHH") that increased reporter activity to <10% of maximum, or exposed them to a high, near-saturating concentration of SHH ("HiSHH") that increased reporter activity to >95% of maximum (Figure S1B). Compared with non-targeting controls, single guide RNAs (sgRNAs) targeting the positive regulators Smoothened (*Smo*) and G-protein-coupled receptor kinase 2 (*Adbrk1* or *Grk2*) reduced SHH-induced GFP fluorescence and sgRNAs targeting the negative regulators Patched 1 (*Ptch1*) and Suppressor of Fused (*Sufu*) induced a SHH-independent increase in GFP fluorescence (Figure S1C). These results show that GFP fluorescence in NIH/3T3-CG cells provided a quantitative readout of Hh signaling with a dynamic range that was sensitive to the perturbation of both positive and negative regulators and consequently could be used for a genome-wide pooled screen based on cell sorting.

We conducted four genome-wide screens in NIH/3T3-CG cells, each in duplicate, for a total of eight independent screens using the lentivirus-based Brie library (Figure 1A) (Doench et al., 2016). In the screen for positive regulators (hereafter referred to as the "HiSHH_Bot10%" screen), we treated NIH/3T3-CG cells with HiSHH and then isolated ~2 million with the lowest 10% of GFP fluorescence by fluorescence-activated cell sorting (FACS) (Figure 1B). In the three screens for negative regulators and attenuators, we isolated ~1 million cells with the highest 5% of GFP fluorescence after treatment with HiSHH (the "HiSHH_Top5%" screen), LoSHH (the "LowSHH_Top5%" screen), or NoSHH (the "NoSHH_Top5%" screen) (Figures 1C–1E). For each screen, sgRNA enrichment and depletion in the selected population was compared with the corresponding unsorted control population across two independent replicates using the MAGeCK algorithm (Li et al., 2014).

Screens Identified Most Known Regulators of Hh Signaling and Many Genes Linked to Cilia and Ciliopathies

The results of the screens were visualized using volcano plots (Figures 1B–1E; complete tabulated results provided in Table S1). The asymmetric nature of the plots showed that the statistical power to detect depletion in the sorted population was lower than the power to detect enrichment. Hence, all further analyses only considered genes that were significant based on their false discovery rate (FDR)-corrected p values for enrichment in the sorted population.

Taken together, the screens identified a majority of the components at all levels of Hh signaling from the cell surface to the nucleus (see Figure 2A for a comprehensive summary and Table S2 for a manually curated list of Hh genes). A top hit in all three negative regulator screens was the main receptor for SHH, PTCH1 (Figures 1C–1E). In the absence of Hh ligands, PTCH1 blocks the activity of the G-protein-coupled receptor (GPCR)-family protein SMO, which transmits the Hh signal across the membrane. As expected, SMO was a top hit in the positive regulator screen, along with genes encoding several proteins that have been previously implicated in SMO signaling, including GRK2 and the SMO-interacting proteins EVC, IQCE, and LZTFL1 (Figures 1B and 2A).

SMO antagonizes the effects of two major cytoplasmic negative regulators: Suppressor of Fused (SUFU) and Protein Kinase A (PKA) (Figure 2A). SUFU and PKA promote the proteolytic conversion of GLI3 into a transcriptional repressor (GLI3R) and also block the formation of the transcriptional activators GLI2A and GLI3A. SUFU was a top hit in all negative regulator screens (Figures 1C–1E). PKA was not identified, likely because its catalytic subunit can be encoded by two redundant genes (*Prkaca* and *Prkacb*). However, several proteins that regulate PKA activity and have previously been implicated in Hh signaling were prominent screen hits (Figure 2A). Two proteins that increase PKA activity and so function as negative regulators of Hh signaling, adenylate cyclase (ADCY6) and its activating heterotrimeric G-protein subunit $G\alpha_S$ (the product of *Gnas* gene), were identified in the negative regulator screens. Conversely, $G\alpha_{i3}$ (the product of *Gnai3* gene), a heterotrimeric G-protein subunit that inhibits adenylate cyclases and reduces PKA activity, was identified as a positive regulator (Figure 2A). GPR161, a $G\alpha_S$-coupled negative regulator of Hh signaling, was not targeted by the Brie library, but TULP3 and GRK2, implicated as positive and negative regulators of GPR161 function respectively, were identified in screens for attenuating regulators (LoSHH_Top5%) and positive regulators (HiSHH_Bot10%), respectively.

At the level of the Hh-responsive transcription factors (TFs), our screens for negative regulators identified proteins (GSK3β, FBWX11, KIF7, and RAB23) that promote the biogenesis of GLI3R and proteins (MED12 and BCOR) that promote the transcriptional repression of Hh target genes (Figure 2A). Conversely, the HiSHH_Bot10% screen for positive regulators identified components (DYRK1A, BRD2, and PRMT1) that promote activation of Hh target genes (Figure 2A). Taken together, these results demonstrated that our screening strategy based on cell sorting could identify many non-redundant positive, negative, and attenuating regulators of Hh signaling.

To provide a more unbiased view of the functional classes of genes identified by our screens, we performed gene set analyses on the 641 genes across all four screens that were enriched in the sorted populations with an FDR-corrected p value <0.1 (Table S1). With this list as the input, Gene Ontology (GO) analysis identified "cilium morphogenesis" as the most enriched term (FDR-corrected p value $\sim 10^{-19}$). Strikingly, nearly all of

Figure 1. CRISPR-Based Screens to Identify Genes that Influence Hh Signaling

(A) The screening strategy used to identify positive regulators, negative regulators, and attenuators of Hh signaling (see text for details). Bot, bottom.

(B–E) Volcano plots from the four screens. For each gene, the x axis shows its enrichment or depletion, calculated as the mean of all four sgRNAs targeting the gene, in the sorted population relative to the corresponding unsorted population, and the y axis shows statistical significance as measured by the false discovery rate (FDR)-corrected p value. The horizontal dashed line represents a p value threshold of 0.1. Positive and negative Hh pathway regulators are labeled as large teal and magenta dots, respectively; cilia genes as medium blue dots; all other genes as small gray dots. See also Figure S1 and Table S1.

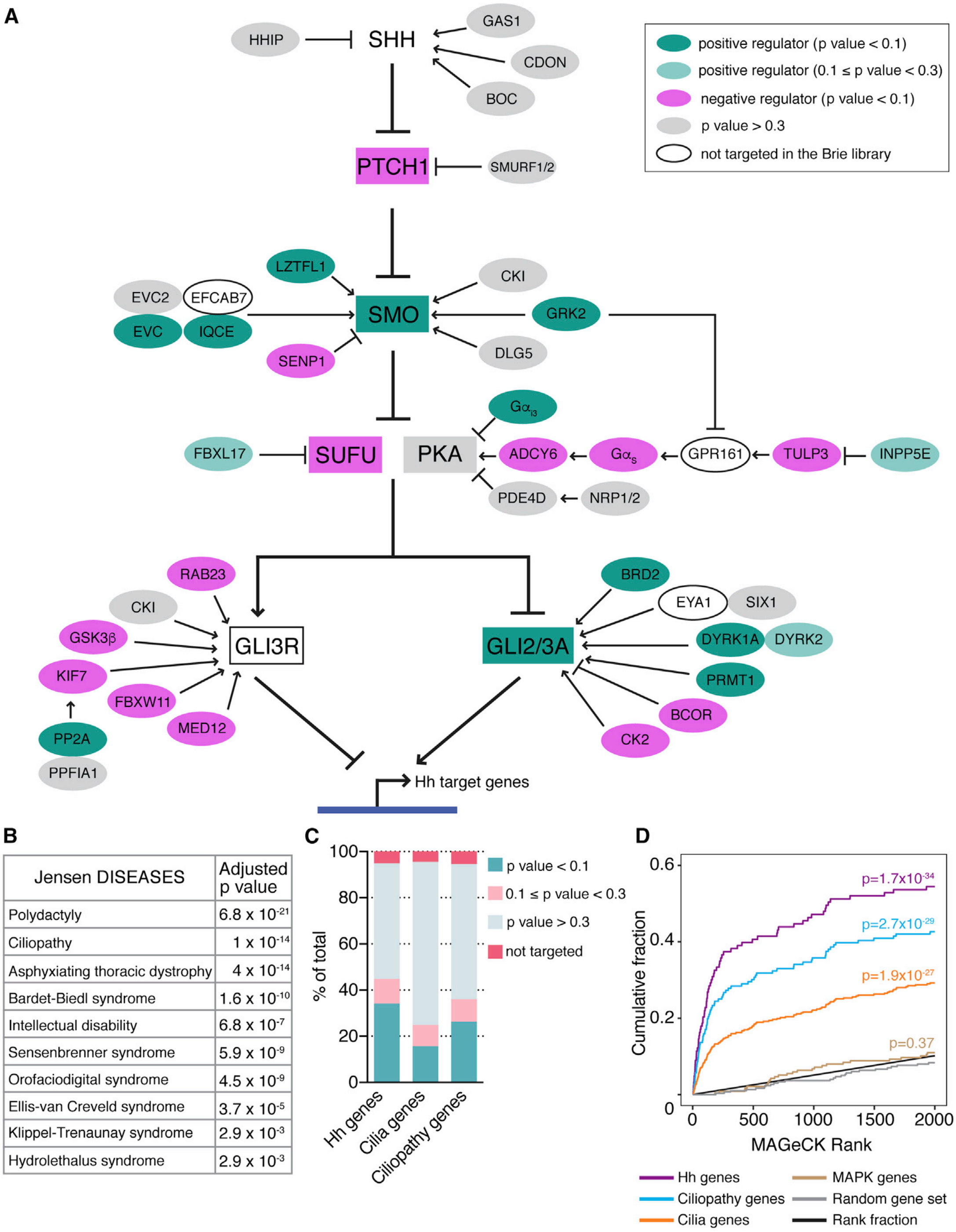

Figure 2. Statistically Significant Screen Hits Are Enriched in Known Hh and Cilia Genes

(A) Depiction of core (rectangle) and accessory (oval) Hh pathway components, colored according to the FDR-corrected p value for their enrichment in the selected cell populations.

(B) Results of enrichment analysis showing the most significant associations between hits from all screens (with an FDR-corrected p value <0.1) in the Jensen database of disease-gene associations (Pletscher-Frankild et al., 2015).

(C) Fractional enrichment of known Hh, cilia, and ciliopathy genes in all screens.

(D) Cumulative distribution function of HiSHH_Bot10% screen ranks for 123 Hh genes, 407 cilia genes, 176 ciliopathy genes, and two control gene lists (237 MAPK genes from the KEGG database and a random set of 300 genes). Statistical significance was calculated based on the top 2,000 genes using the hypergeometric test. See also Table S2.

the top disease associations found in this gene list were known ciliopathies or congenital anomalies associated with defects in cilia or Hh signaling (Figure 2B).

To evaluate the enrichment of cilia-related genes among our screen hits, we used three benchmark gene lists. The first was a manually curated list of 176 genes (hereafter referred to as the ''Hh genes'' list shown in Table S2), which included all genes linked to a Hh-related signaling defect or phenotype in the PubMed database. A large number of these Hh genes were cilia-related genes, since even subtle defects in cilia structure or function can impair Hh signal transduction (Bangs and Anderson, 2016). We also compared the list of screen hits with two recently published lists of (1) all known ciliary genes (n = 426) and (2) all known ciliopathy genes (n = 186) that build on the gold-standard SYSCILIA compendium (Reiter and Leroux, 2017; van Dam et al., 2013). Taken together, the four screens identified 40% of Hh genes, 20% of all cilia genes, and 30% of all ciliopathy genes as significant hits (Figure 2C). Most of the cilia-related genes were identified in the HiSHH_Bot10% screen for positive regulators or the NoSHH_Top5% screen (Figures 1B and 1E). Cilia genes were hits in the NoSHH_Top5% screen because ciliary defects prevent the formation of GLI3R and thus lead to the transcriptional de-repression of some Hh target genes (Huangfu and Anderson, 2005). A cumulative distribution function plot showed that the top 2,000 hits from the HiSHH_Bot10% screen were highly enriched for cilia- and ciliopathy-related genes from each of these three benchmark lists (Figure 2D). Since cilia are essential for the transduction of high-level Hh signals, very few ciliary genes emerged as hits in the HiSHH_Top5% and LoSHH_Top5% screens (Figures 1C and 1D).

Our results are consistent with studies from mouse and human genetics that indicate the close and complex relationship between Hh signaling and primary cilia at multiple levels of the pathway (Bangs and Anderson, 2016). Over the past decade, these studies have found that the Hh pathway is exquisitely sensitive to even subtle changes in cilia structure or function. Conversely, abnormalities in Hh signaling, read-out through phenotypes in mice or humans, have served as sensitive indicators of ciliary defects (Huangfu et al., 2003). CRISPR-based screens in cultured cells using fluorescent transcriptional reporters to select for cells with altered levels of Hh signaling can provide an orthogonal strategy for the functional identification of ciliary genes.

Validation of Candidate Genes in Two Different Cell Types

We used a multi-step strategy (summarized in Figure S2A) to select genes likely to have the strongest and most general effects on Hh signaling from the list of top-ranked hits from all four screens. Of the 69 genes that were tested in pooled cell lines during phase I of our validation strategy, genes targeted by at least one high-quality guide that mapped to a unique position in the mouse genome and either (1) decreased GFP fluorescence by $\geq$ 2-fold (for positive regulators) or (2) increased GFP fluorescence by $\geq$ 1.5-fold (for negative regulators) were selected for phase II of analysis (Figure S2). The five genes that encoded putative positive regulators were *Cep350*, *Pdcl*, *Rab34*, *Fkbp10*, and *Tubd1* (Figure S2B). Candidate negative regulators included two genes linked to heterotaxy and congenital heart defects (*Mgrn1* and *Megf8*), two genes linked to hereditary cavernous malformations (*Ccm2 and Pdcd10*), *Mesdc1*, and an unstudied open reading frame annotated as BC030336 in mouse and *C16orf52* in humans (Figures S2C–S2E). We named this gene *Atthog* for Attenuator of Hedgehog.

For each of these 11 genes, we generated 2–3 independent clonal NIH/3T3 cell lines with loss-of-function mutations. To exclude off-target effects of genome editing, we used sgRNAs that were different from the Brie library guides used in the initial screens and the first phase of validation (sgRNA sequences, sites targeted by the sgRNAs within each gene, and gels showing successful editing are shown in Table S3). To exclude genes that only influenced the synthetic GLI-GFP reporter, we evaluated Hh signaling in these clonal cell lines by measuring the induction of *Gli1*, an endogenous direct Hh target gene commonly used to measure signaling strength. Among cell lines lacking the putative positive regulators, *Rab34*$^{-/-}$, *Tubd1*$^{-/-}$, and *Pdcl1*$^{-/-}$ NIH/3T3 cells showed the strongest defects in SHH-induced *Gli1* expression (Figure 3A). Among cell lines lacking negative regulators, *Atthog*$^{-/-}$, *Megf8*$^{-/-}$, and *Mgrn1*$^{-/-}$ NIH/3T3 cells showed constitutive, SHH-independent induction of *Gli1* (Figure 3B). Interestingly, exposure of cells to LoSHH, which barely increased *Gli1* transcription in wild-type (WT) cells, produced maximal induction of *Gli1* in mutant cell lines, showing that loss of these genes sensitized cells to SHH (Figure 3B).

Cultured spinal neural progenitor cells (NPCs) function as an excellent model system for studying Hh signaling. When NPCs are induced to differentiate, their adopted identity is a direct product of Hh signaling strength (Cohen et al., 2013; Gouti et al., 2014; Jessell, 2000). To test if our top candidates modulated Hh signaling in this physiologically relevant cell system, we individually knocked out the ten genes in mouse embryonic stem cells (mESCs), differentiated these cells toward spinal NPCs, and then exposed them to varying SHH concentrations. To facilitate this analysis, we used a recently described mESC line stably carrying dual fluorescent Hh signaling reporters: a GLI-Venus synthetic transcriptional reporter, analogous to the GLI-GFP reporter used in NIH/3T3 cells, and an OLIG2-mKate reporter in which the far-red fluorescent protein mKate was fused to the C terminus of endogenous *Olig2* via a self-cleaving peptide (https://doi.org/10.1101/104307). OLIG2-mKate fluorescence reports on the SHH-induced differentiation of mESCs into spinal motor neuron progenitors.

mESCs harboring this dual Hh signaling reporter system were differentiated into NPCs and FACS was used to simultaneously measure SHH-induced increases in Venus (Figure 3C) and mKate (Figure 3D) fluorescence. As in NIH/3T3 cells, loss-of-function mutations in *Rab34*, *Pdcl*, and *Tubd1* led to the strongest decreases in signaling. The loss of *Mgrn1*, *Atthog*, and *Megf8* function had minimal effects on the basal levels of Venus and mKate fluorescence (Figures 3C and 3D). However, these mutant NPCs were hyper-sensitive to SHH. Low concentrations of SHH, which increased Venus and mKate fluorescence in WT cells by less than 2-fold, produced a 4–5-fold increase in *Mgrn1*$^{-/-}$ cells and induced maximum reporter fluorescence in *Atthog*$^{-/-}$ and *Megf8*$^{-/-}$ cells. Hence, *Mgrn1*, *Megf8*, and *Atthog* are attenuating modifiers of Hh signaling in NPCs: their loss enhances the sensitivity of cells to SHH.

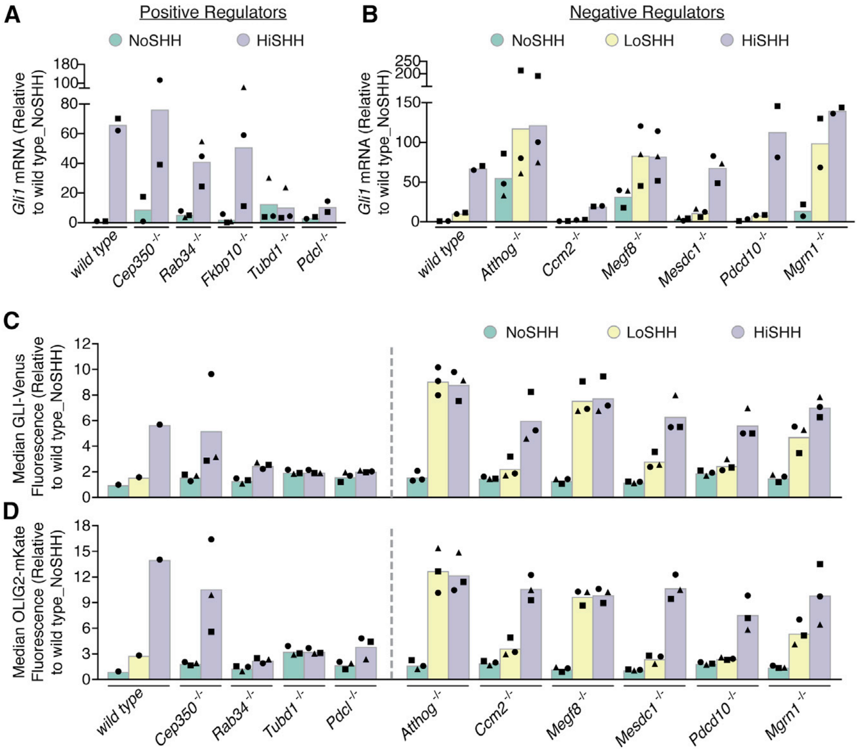

Figure 3. Clonal Lines Carrying Deletions in Top Hits Identify Regulators of Hh Signaling

(A and B) Hh signaling strength was assessed in clonal, mutant NIH/3T3 cells by measuring *Gli1* mRNA by qRT-PCR after LoSHH or HiSHH treatment.

(C and D) Hh signaling was assessed in clonal, mutant NPCs exposed to LoSHH or HiSHH using either a fluorescent reporter of target gene induction (GLI-Venus), (C) or a fluorescent reporter of motor neuron differentiation (OLIG2-mKate), (D). Bars represent the mean *Gli1* mRNA level (A and B) or mean reporter fluorescence (C and D) from 2 to 3 independent clonal lines. Each data point, derived from a separate clonal cell line, represents either the mean *Gli1* mRNA level from two technical replicates (A and B) or the median reporter fluorescence (10,000 cells) from two independent experiments (C and D). See also Figure S2 and Table S3.

In summary, data from clonal derivatives of two distinct cell types using both target gene expression assays and differentiation assays identified six genes for further analysis: the positive regulators *Rab34*, *Tubd1*, and *Pdcl*, and the negative regulators *Mgrn1*, *Megf8*, and *Atthog*.

Positive Regulators of Hh Signaling Are Required for Primary Cilia Functions

Two assays were used to decipher how the six selected genes influenced Hh signaling. First, we used immunoblotting to measure protein levels of key Hh pathway components in clonal NIH/3T3 cell lines carrying loss-of-function mutations in each gene. GLI1 and PTCH1 are encoded by direct Hh target genes and so their abundance provides a transcriptional readout of signaling. GLI3R abundance provides a non-transcriptional readout for signaling, since its biogenesis is negatively regulated by Hh signals. Importantly, GLI3R levels in the absence of SHH report on the integrity of primary cilia: loss of cilia leads to a reduction in GLI3R and the consequent de-repression of some Hh target genes (Huangfu and Anderson, 2005).

Second, we measured the dynamic, SHH-regulated localization of endogenous Hh pathway components at primary cilia by immunofluorescence (IF) (Briscoe and Thérond, 2013). In the absence of SHH, PTCH1 is concentrated in and around the ciliary membrane. SHH binding to PTCH1 leads to its clearance from the cilium and cell surface, allowing SMO to accumulate in the ciliary membrane and adopt an active conformation. SMO activation is correlated with increased accumulation of GLI2/3 at the tips of cilia. Trafficking of the SUFU-GLI2/3 complex through cilia is required for the conversion of GLI2 and GLI3 into potent transcriptional activators. Thus, ciliary levels of PTCH1, SMO, and GLI2 can be used to evaluate the status of signal propagation at multiple levels in the pathway (Figure 4A).

We emphasize that all assays presented hereafter for the analysis of both positive and negative regulators were performed in two independent clonal cell lines for each genotype. In some cases, analysis of only one clonal cell line is shown in the main figure, with data from the second clonal cell line presented in a supplemental figure panel noted in the figure legend.

Tubd1 encodes δ-Tubulin, a divergent tubulin conserved across eukaryotes, whose presence in genomes is correlated with the presence of cilia (Figures S3A and S3B). Mutations in the *Chlamydomonas* ortholog of *Tubd1*, encoded by the *UNI3* gene, cause defects in the assembly of flagella, which are analogous to vertebrate motile cilia (Dutcher and Trabuco, 1998). Centrioles in human cells lacking TUBD1 are unstable and fail to undergo maturation (Wang et al., 2017). GLI1 induction was abolished in *Tubd1*$^{-/-}$ NIH/3T3 cells (Figures 4B and S3E) because nearly 100% of these cells lacked primary cilia (Figure 4C).

RAB34 is a Golgi-associated small GTPase implicated in regulating the sub-cellular distribution of lysosomes (Wang and Hong, 2002). RAB34 is confined to metazoans, but it is related to the RAB8 proteins that are widely present across most eukaryotic lineages and involved in ciliogenesis (Figures S3A and S3C). Interestingly, the genomic region upstream of *Rab34* has a functional binding site for GLI proteins and *Rab34* was shown to be a Hh target gene in the mouse limb bud (Vokes et al., 2007). The phenotypes of *Rab34*$^{-/-}$ mouse embryos, which include polydactyly and exencephaly, are suggestive of defects in Hh signaling and cilia (Dickinson et al., 2016). While SHH-induced GLI1 protein levels were significantly reduced in *Rab34*$^{-/-}$ cells, these cells could transduce low-level Hh signals: Gli3R levels declined when SHH was added (Figures 4B and S3E). Immunofluorescence studies revealed that *Rab34*$^{-/-}$ cells had a reduced frequency of primary cilia compared with WT cells, although the cilia that did form had normal lengths (Figures 4D and S3F). SHH-triggered SMO accumulation was impaired in the residual cilia of

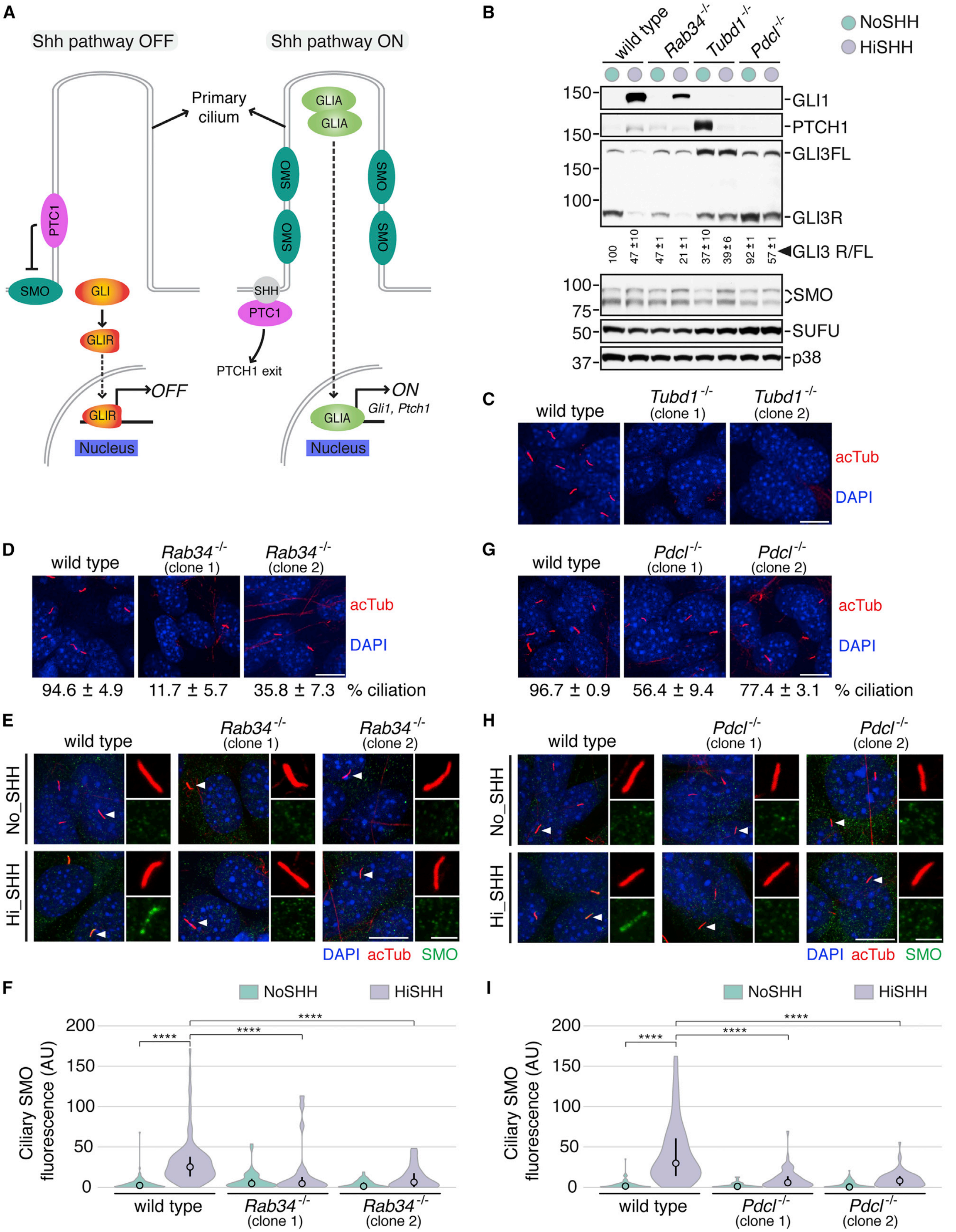

(legend on next page)

$Rab34^{-/-}$ cells (Figures 4E and 4F), suggesting a defect in ciliary trafficking. The partial phenotypes observed in $Rab34^{-/-}$ cells may be related to redundancy with the vertebrate-specific paralogs RAB36 or RAB34b (Figure S3C).

PDCL, a member of the phosducin-like family of proteins, contains an N-terminal α-helical domain and a C-terminal domain of the thioredoxin superfamily. PDCL positively regulates heterotrimeric G-protein signaling by functioning as a chaperone for the assembly of G-protein $\beta\gamma$ dimers (Lukov et al., 2005). In many systems, disruption of the *Pdcl* gene leads to a decline in $G\beta\gamma$ protein levels and failure of G-protein signaling. Phylogenetic analysis of the phosducin-like clade hints at a cilium-associated function for *Pdcl*, in addition to a role in heterotrimeric G-protein signaling (Figures S3A and S3D). SHH was unable to induce the expression of the direct target genes *Gli1* or *Ptch1* in $Pdcl^{-/-}$ cells, although it could induce a decrease in GLI3R, consistent with low-level Hh signaling (Figures 4B and S3E). The frequency of primary cilia was modestly reduced in $Pdcl^{-/-}$ cells, without significant change in ciliary length (Figures 4G and S3F). However, there was a severe defect in SHH-induced accumulation of SMO in the ciliary membrane (Figures 4H and 4I). We note that PDCL could also influence Hh signaling by impairing the activity of heterotrimeric G proteins, such as the screen hit $G\alpha_{i3}$ (Figure 1B), that antagonize PKA activity.

In conclusion, all three genes that emerged as the top hits among positive regulators of Hh signaling regulate ciliary functions. While further work will be required to elucidate the precise mechanisms, two of these genes, *Rab34* and *Pdcl*, have never been linked to cilia. Their involvement suggests cilium-associated roles for Rab-regulated vesicle trafficking and heterotrimeric G-protein signaling. More generally, our analysis of positive regulators reaffirms the concept that genetic screens based on Hh signaling phenotypes can be used to discover genes that regulate the function of cilia.

Negative Regulators of Hh Signaling Suppress SMO Accumulation in Primary Cilia

We undertook a more in-depth analysis of the signaling attenuators *Megf8*, *Mgrn1*, and *Atthog*, because mutations that increase signaling activity are more likely to be in genes that encode (or directly regulate) core signaling components and less likely to cause non-specific or indirect effects.

Megf8 encodes a single-pass, type I transmembrane (TM) protein containing a short cytoplasmic tail and a large multi-domain extracellular region containing modules associated with cell adhesion (CUB and EGF domains) and binding to sugars (the β-propeller forming kelch repeats) (Figure S4A). It is conserved across metazoans and their closest sister group, the choanoflagellates, suggesting an origin pre-dating Hh signaling (Figure S3A). *Mgrn1* (or *Rnf156*) is conserved throughout eukaryotes, regardless of the presence of Hh signaling or cilia, and encodes a ubiquitin E3-ligase containing a putative substrate-binding domain at the N terminus and a RING-finger domain (Figures S3A, S4B, and S4C).

In contrast with positive regulators, the frequency of primary cilia was unaltered in cells carrying loss-of-function mutations in *Megf8*, *Mgrn1*, or *Atthog* (Figure S5A). Consistent with assays based on *Gli1* mRNA levels (Figure 3B), measurement of GLI1, PTCH1, and GLI3R protein levels showed that $Atthog^{-/-}$ and $Megf8^{-/-}$ NIH/3T3 cells demonstrated partial signaling activity even in the absence of Hh ligands (Figures 5A and S5B). In addition, all three mutant cell lines were hyper-responsive to SHH, with LoSHH leading to full activation of signaling. Interestingly, SMO levels were significantly higher in $Atthog^{-/-}$ and $Megf8^{-/-}$ cells compared with WT or $Mgrn1^{-/-}$ cells. The increase in SMO protein in $Atthog^{-/-}$ and $Megf8^{-/-}$ cells was selectively observed in the population that has traversed the endoplasmic reticulum (ER) (post-ER SMO) and thus migrates more slowly on an SDS-PAGE gel due to changes in glycosylation catalyzed by enzymes found in the Golgi (Figure 5A). Biotinylation experiments using a non-cell-permeable probe confirmed that cell-surface levels of SMO were markedly elevated in $Atthog^{-/-}$ and $Megf8^{-/-}$ cells but not in $Mgrn1^{-/-}$ cells (Figure 5B).

Visualization of the sub-cellular localization of SMO by confocal fluorescence microscopy revealed that it accumulated to very high levels in the ciliary membrane of $Atthog^{-/-}$ and $Megf8^{-/-}$ cells, even in the absence of SHH (Figures 5C, 5D, and S5C). The SMO in cilia could be detected in non-permeabilized cells by an antibody against the extracellular region of SMO, demonstrating that it was localized at the cell surface (Figure S5D). Ciliary SMO levels in $Atthog^{-/-}$ and $Megf8^{-/-}$ cells were much higher than WT cells treated with saturating SHH and even higher than $Ptch1^{-/-}$ cells, which show fully activated, ligand-independent signaling. Ciliary SMO levels in $Mgrn1^{-/-}$ cells were more variable but nonetheless significantly elevated compared with WT cells treated with saturating SHH, even though total SMO levels were unaffected by the loss of *Mgrn1* (Figures 5C, 5D, and S5C).

The high levels of ciliary SMO in $Atthog^{-/-}$ and $Megf8^{-/-}$ made it difficult to assess whether SHH could further increase SMO in cilia, although images taken at low gain settings revealed responsiveness to HiSHH (Figures 5D and S5C). In $Mgrn1^{-/-}$ cells, SHH did clearly increase SMO accumulation in cilia. The lower baseline ciliary SMO levels in $Mgrn1^{-/-}$ cells correlated with the consistently lower degree of Hh signal potentiation

Figure 4. Ciliary Integrity Is Impaired in NIH/3T3 Cells Lacking TUBD1, RAB34, and PDCL

(A) Ciliary localization of Hh pathway components in the presence and absence of SHH.

(B) Immunoblots showing the abundance of Hh pathway proteins and a loading control (p38) in extracts of $Rab34^{-/-}$, $Tubd1^{-/-}$, or $Pdcl^{-/-}$ NIH/3T3 cells. Data from an independent set of cell lines are shown in Figure S3E.

(C, D, and G) Acetylated tubulin (acTub, red) immunostaining was used to visualize primary cilia and determine the frequency of ciliated cells in wild-type NIH/3T3 cells and two independent $Tubd1^{-/-}$ (C), $Rab34^{-/-}$ (D), or $Pdcl^{-/-}$ (G) clonal cell lines. DAPI (blue) marks nuclei.

(E and H) HiSHH-induced ciliary SMO (green) in $Rab34^{-/-}$ (E) and $Pdcl^{-/-}$ (H) cells. Arrowheads identify magnified cilia shown to the right of each panel.

(F and I) The distribution of SMO fluorescence intensity (n ~100 cilia/condition) is shown on a violin plot (see STAR Methods).

Statistical significance was determined by the Kruskal-Wallis test; ****$p < 0.0001$. Scale bars, 10 μm in merged panels and 2 μm in zoomed displays. See also Figure S3.

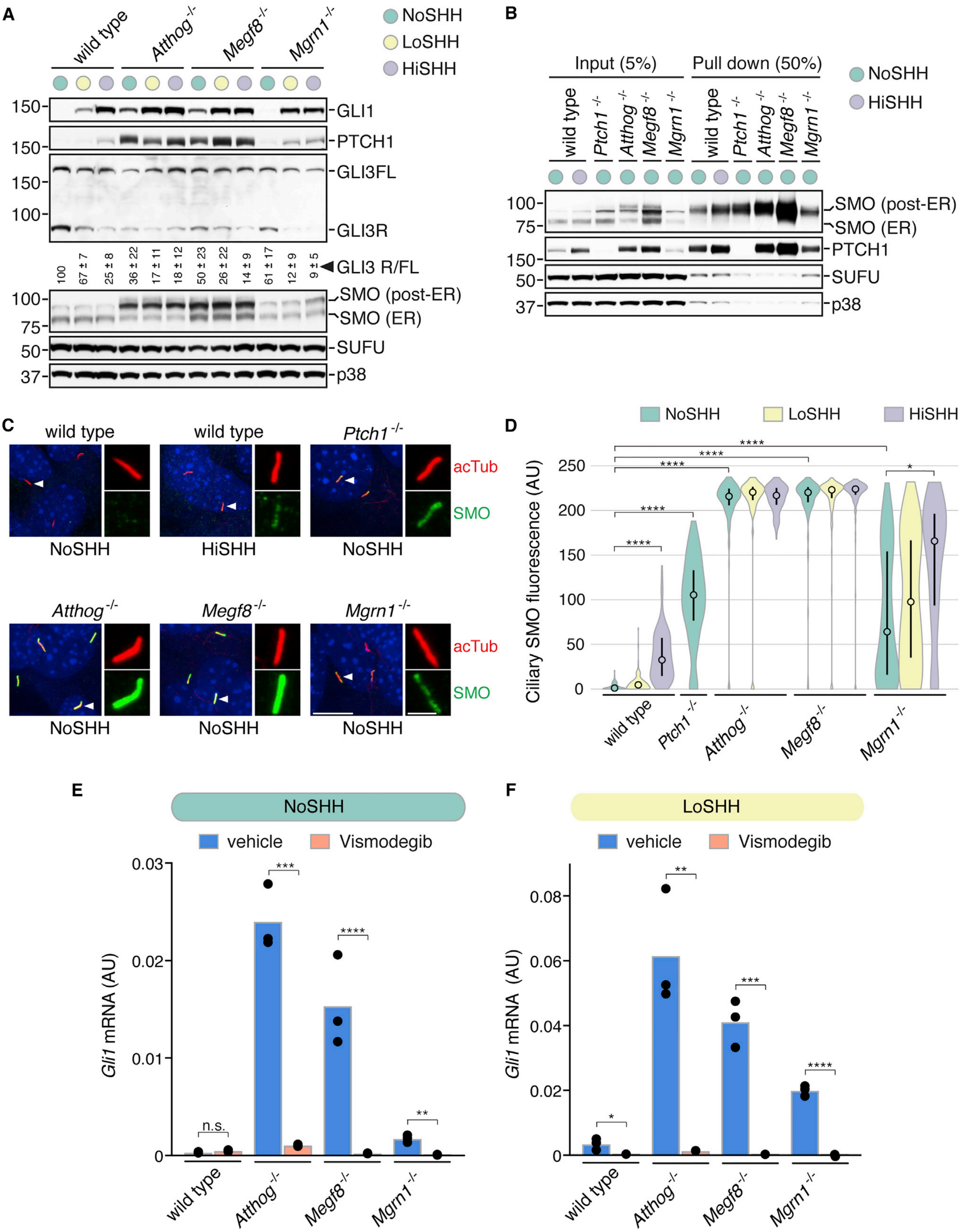

(legend on next page)

seen in *Mgrn1*$^{-/-}$ cells compared with *Atthog*$^{-/-}$ and *Megf8*$^{-/-}$ cells (Figures 3B–3D). The function of *Mgrn1* may be partially redundant with the vertebrate-specific paralog *Rnf157*, as proteins encoded by these genes display ~50% identity (Figure S4B).

When SMO ciliary accumulation is accompanied by SMO activation, GLI2 protein levels at the tips of cilia increase, as seen when PTCH1 is inactivated by SHH or genetically disrupted in *Ptch1*$^{-/-}$ cells (Figures S5E and S5F). GLI2 levels were elevated at the tips of cilia in *Atthog*$^{-/-}$ and *Megf8*$^{-/-}$ cell lines and (even in the absence of SHH) were comparable with levels seen in activated WT cells or *Ptch1*$^{-/-}$ cells, supporting the presence of increased SMO activity (Figures S5E and S5F).

Taken together, data from target gene expression studies and ciliary protein localization studies demonstrated that the loss of *Atthog*, *Megf8*, and *Mgrn1* led to both constitutive, SHH-independent signaling and potentiation of SHH-driven signaling. The striking increase in ciliary SMO pointed to an effect on SMO itself or a step upstream of SMO in the pathway. Activation of signaling downstream of SMO, for example by the loss of SUFU, does not cause the increased accumulation of SMO in primary cilia (Figures S6A and S6B). To conclusively address this point, we treated all three mutant cell lines with the direct SMO antagonist vismodegib. Vismodegib completely abolished signaling, both in the absence and the presence of SHH, proving that these negative regulators function at the level of SMO or a step upstream of SMO, such as SHH reception or PTCH1 function (Figures 5E, 5F, S6C, and S6D).

These negative regulators are unlikely to regulate ligand reception because signaling is increased in cell lines lacking these proteins even in the complete absence of SHH (Figure 3B). To address the issue of PTCH1 function, we assessed the subcellular localization of PTCH1 in *Atthog*$^{-/-}$, *Megf8*$^{-/-}$, and *Mgrn1*$^{-/-}$ cells and compared it with PTCH1 localization in cells lacking the downstream negative regulator SUFU (*Sufu*$^{-/-}$ cells). Increased signaling in all of these cell lines drives elevated levels of *Ptch1* transcription and hence PTCH1 protein abundance (Figures 5A and S5B). As in WT and *Sufu*$^{-/-}$ cells, PTCH1 was localized in a punctate pattern in and around primary cilia in all three mutant cell lines in the absence of SHH (Figures S6E and S6F). Moreover, PTCH1 was cleared from cilia upon SHH addition, showing that SHH could still bind and downregulate PTCH1 normally in the absence of these genes (Figure S6F). Thus, increased SMO activity and SMO ciliary levels cannot be due to the faulty trafficking of PTCH1. Despite normal PTCH1 localization, PTCH1 inhibitory activity toward SMO could be compromised. This scenario is unlikely for two reasons. First, despite the high levels of SMO at cilia in *Atthog*$^{-/-}$ and *Megf8*$^{-/-}$ cells, they remained responsive to SHH, suggesting that PTCH1 is still able to partially restrain SMO activity (Figures 3B–3D). Second, SMO levels in cilia of *Atthog*$^{-/-}$ and *Megf8*$^{-/-}$ cells were considerably higher than in cilia of *Ptch1*$^{-/-}$ cells, implying that the loss of these genes must be performing a role other than just reducing PTCH1 activity (Figure 5D).

In summary, the three negative regulators that emerged as top hits in our screens converged on the same step in signaling, regulation of SMO ciliary localization or activity, suggesting that they may be involved in a common pathway.

ATTHOG Is a Tetraspan Protein that Regulates SMO Stability

We analyzed the function of *Atthog* in more detail. ATTHOG is an integral membrane protein that belongs to the large tetraspan superfamily. Based on phylogeny, we found ATTHOG to be nested within the radiation of a clade of tetraspans, which includes the claudin-like proteins, best known as components of inter-cellular tight junctions (Figure 6A). Therein ATTHOG is most closely related to three paralogous proteins, including one (TMHS) that has been implicated in human deafness as a key component of the mechanotransduction machinery of cochlear hair cells (Xiong et al., 2012). Sequence alignments of *Atthog* orthologs (Figure S7A) predict that it has four transmembrane helices without a cleaved signal sequence, a disulfide-linked extracellular domain, and a conserved cysteine in the cytoplasmic tail that is predicted to be palmitoylated (Figure 6B). We also noted an unusual conservation of charged and polar residues in the TM helices that could form an aqueous channel that allows permeation of a hydrophilic solute or could mediate protein interactions within the plane of the membrane (Figure 6B).

The loss of ATTHOG led to an increase in the post-ER, cell-surface pool of SMO (Figure 5B). When *Atthog*$^{-/-}$ cells were rescued by the re-expression of *Atthog*, steady-state SMO levels were reduced to those seen in WT cells (Figure 6C) and SMO was cleared from primary cilia (Figures 6D and 6E). ATTHOG itself was localized to the ciliary membrane, plasma membrane, and Golgi (Figures 6D and S7B). In control experiments, the loss of ATTHOG did not alter levels of Frizzled receptors (Figure S7C), the closest relatives of SMO in the GPCR family, and did not change signaling responses to either WNT or BMP ligands in NIH/3T3 cells (Figures S7D and S7E).

ATTHOG could function by suppressing the accumulation of SMO at the cell surface or by suppressing the active conformation of SMO. Vismodegib, which shifts the SMO conformational equilibrium toward the inactive state, extinguished signaling in *Atthog*$^{-/-}$ cells (Figure 5E) but failed to reduce SMO levels at

Figure 5. ATTHOG, MEGF8, and MGRN1 Regulate Smoothened Signaling

(A) Immunoblots showing the abundance of Hh pathway proteins in extracts of *Atthog*$^{-/-}$, *Megf8*$^{-/-}$, and *Mgrn1*$^{-/-}$ cells. Data from an independent set of cell lines is shown in Figure S5B.

(B) Cell-surface biotinylation to assess levels of SMO and PTCH1 at the plasma membrane of indicated NIH/3T3 cell lines.

(C and D) Representative micrographs (C) and corresponding violin plots (D, n ~ 100 cilia/condition) showing levels of endogenous SMO at primary cilia. Arrowheads point to selected cilia used for zoomed displays shown to the right of each panel. Statistical significance was determined by the Kruskal-Wallis test; *$p < 0.05$ and ****$p < 0.0001$. Data from an independent set of clonal cell lines are shown in Figure S5C.

(E and F) Basal (E) and LoSHH-induced *Gli1* mRNA (F) in *Atthog*$^{-/-}$, *Megf8*$^{-/-}$, and *Mgrn1*$^{-/-}$ cell lines after treatment with vismodegib. The mean (bars) of three independent replicates (black dots) is shown with significance tested using the unpaired Student's t test; *$p < 0.05$, **$p < 0.01$, ***$p < 0.001$, ****$p < 0.0001$, ns, non-significant ($p > 0.05$).

Scale bars represent 10 μm in merged panels and 2 μm in zoomed displays. See also Figures S4–S6.

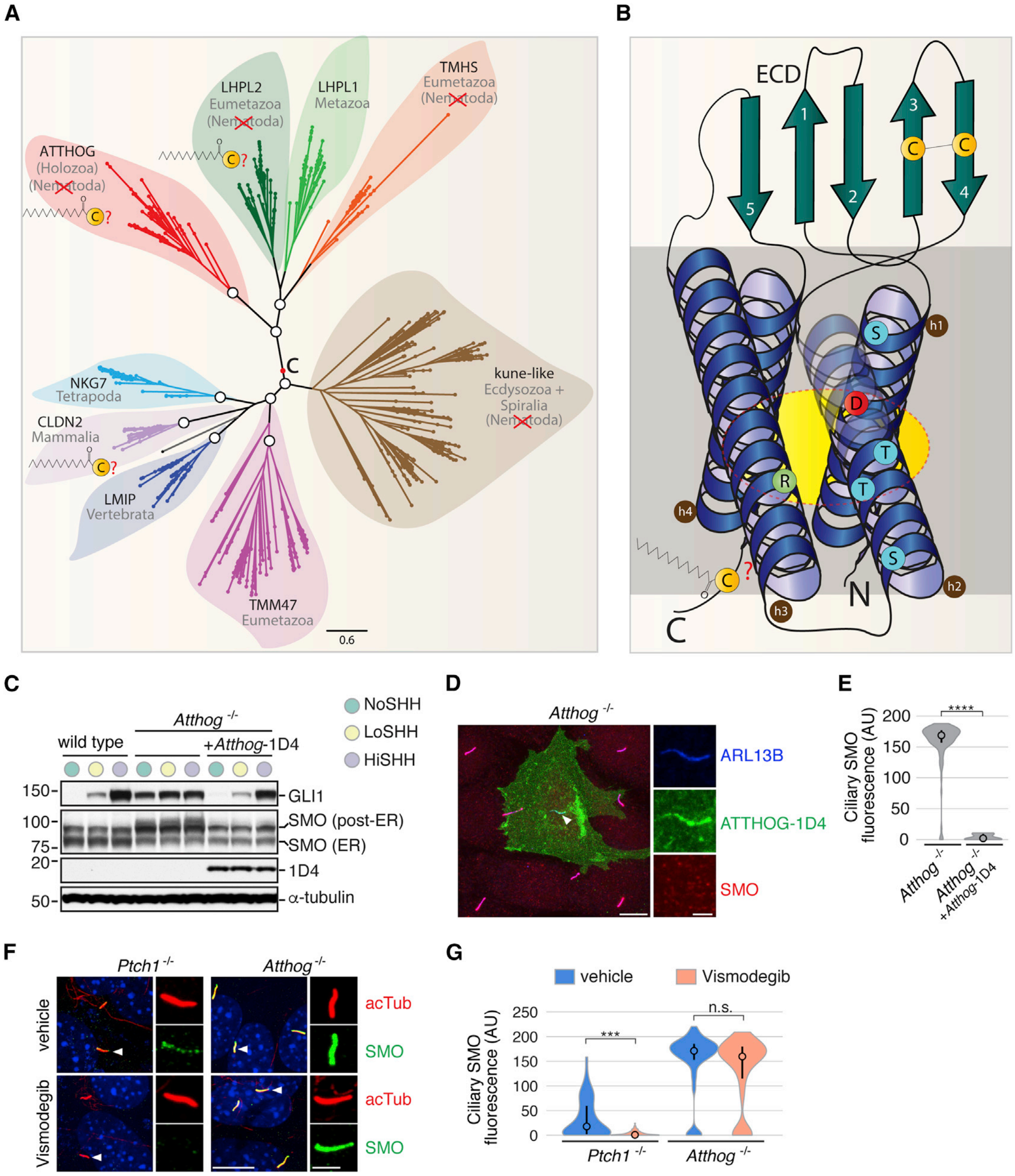

Figure 6. ATTHOG Is Related to the Claudins and Suppresses SMO Levels at Primary Cilia

(A) Phylogenetic relationships of ATTHOG and its relatives within the tetraspan superfamily (see Figure S7A for an alignment). Families forming monophyletic clades are highlighted in distinct colors. The evolutionary provenance of each family is indicated below the gene name, with potential losses in nematodes indicated by a red cross. Families with cysteines predicted to be palmitoylated are marked (filled yellow circles). White circles with black outlines on the nodes denote a support of ≥ 0.9 using the Shimodaira-Hasegawa test on 1,000 resamples. The position of the computed ancestral sequence for rooting is shown with a red circle.

(B) Predicted topology of the four TM helices of ATTHOG. Highlighted features include the disulfide bridge in the extracellular domain (ECD), polar and charged residues in the TM bundle, and a cysteine in the cytoplasmic tail predicted to be palmitoylated. Clustering of hydrophilic residues within the TM bundle is highlighted with a yellow oval.

(C) Immunoblotting showing abundances of the indicated proteins in extracts of WT, *Atthog*$^{-/-}$, and *Atthog*$^{-/-}$ cells stably re-expressing ATTHOG-1D4.

(D) Ciliary localization of SMO and ATTHOG in *Atthog*$^{-/-}$ NIH/3T3 cells transiently transfected with *Atthog*-1D4 (arrowhead). The imaging field shows one *Atthog*-1D4 transfected cell (green, its cilium identified with an arrowhead and marked by ARL13B) surrounded by untransfected cells. Ciliary SMO (red) is lost only in the *Atthog*-transfected cell.

(E) Violin plots showing the abundance of endogenous SMO at cilia of *Atthog*$^{-/-}$ (n = 100) and *Atthog*$^{-/-}$ cells transfected with *Atthog*-1D4 (n = 10).

(F and G) Representative micrographs (F) and corresponding violin plots (G, n = 100 cilia per condition) showing levels of endogenous SMO at primary cilia of *Ptch1*$^{-/-}$ and *Atthog*$^{-/-}$ cells left untreated or treated with vismodegib. Arrowheads point to selected cilia used for zoomed displays shown to the right of each panel. Statistical significance was determined by the Mann-Whitney test (E) or the Kruskal-Wallis test (G); ****$p < 0.0001$, ***$p < 0.001$, ns, non-significant ($p > 0.05$). Scale bars denote 10 μm in merged panels and 2 μm in zoomed displays.

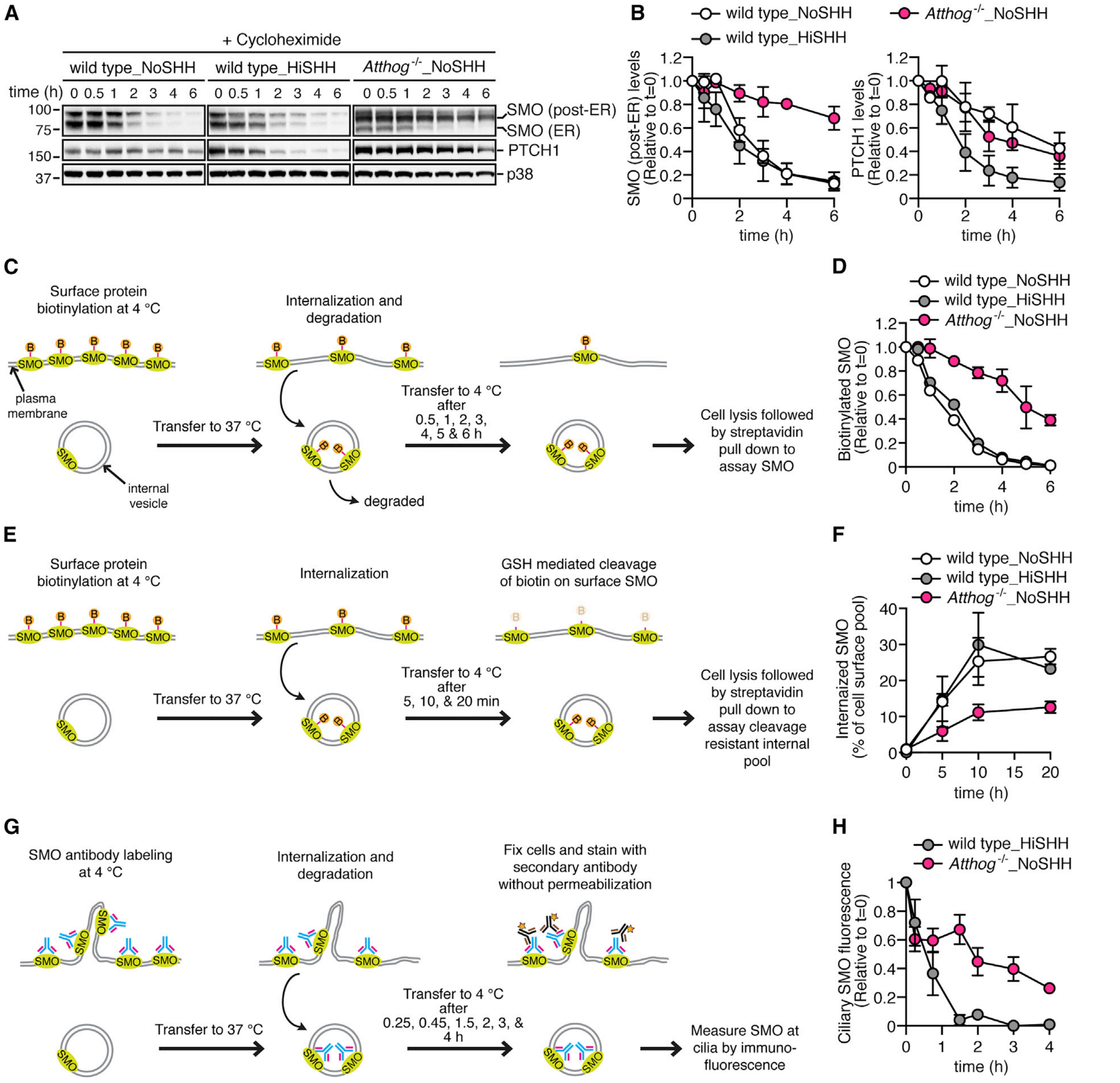

Figure 7. ATTHOG Promotes the Internalization and Degradation of SMO at the Cell Surface

(A and B) After blocking new protein synthesis with cycloheximide, immunoblotting was used to measure the abundances of SMO, PTCH1, and p38 (a control) in WT and *Atthog*$^{-/-}$ cells. Levels of post-ER SMO and PTCH1 at various times after cycloheximide addition were plotted relative to their initial level (set to 1) in (B).

(C and D) Experimental scheme used to monitor the degradation of cell-surface SMO. The fraction of biotinylated SMO remaining at various times after cell-surface labeling is plotted in (D) and shown in Figure S7F.

(E and F) After labeling cell-surface SMO with a thiol-cleavable biotinylation reagent, internalization was monitored by measuring the amount of biotinylated SMO protected from the cell-impermeable reducing agent glutathione (GSH).

(G and H) After labeling live cells with a primary antibody against the extracellular domain of SMO, its levels at cilia were measured at various times after labeling by staining fixed, non-permeabilized cells with a cognate secondary antibody. The fraction of SMO remaining at primary cilia at various times after labeling is shown in (H). Each data point represents a mean ± SD derived from two independent experiments.

primary cilia (Figures 6F and 6G). In contrast, Vismodegib blocked both signaling and SMO ciliary accumulation in *Ptch1*$^{-/-}$ cells (Figures 6F and 6G). Thus, SMO accumulated in cilia of *Atthog*$^{-/-}$ cells regardless of whether it was in an active or inactive conformation, suggesting that ATTHOG primarily influences SMO trafficking rather than SMO activation.

We next sought to understand how ATTHOG reduced the steady-state levels of SMO (Figure 6C). While the abundance of SMO mRNA was unaffected by the loss of ATTHOG (data not shown), the stability of post-ER SMO was markedly greater in *Atthog*$^{-/-}$ cells ($t_{1/2}$ = 10 hr) compared with WT cells ($t_{1/2}$ = 2 hr) (Figures 7A and 7B). In contrast, the half-life

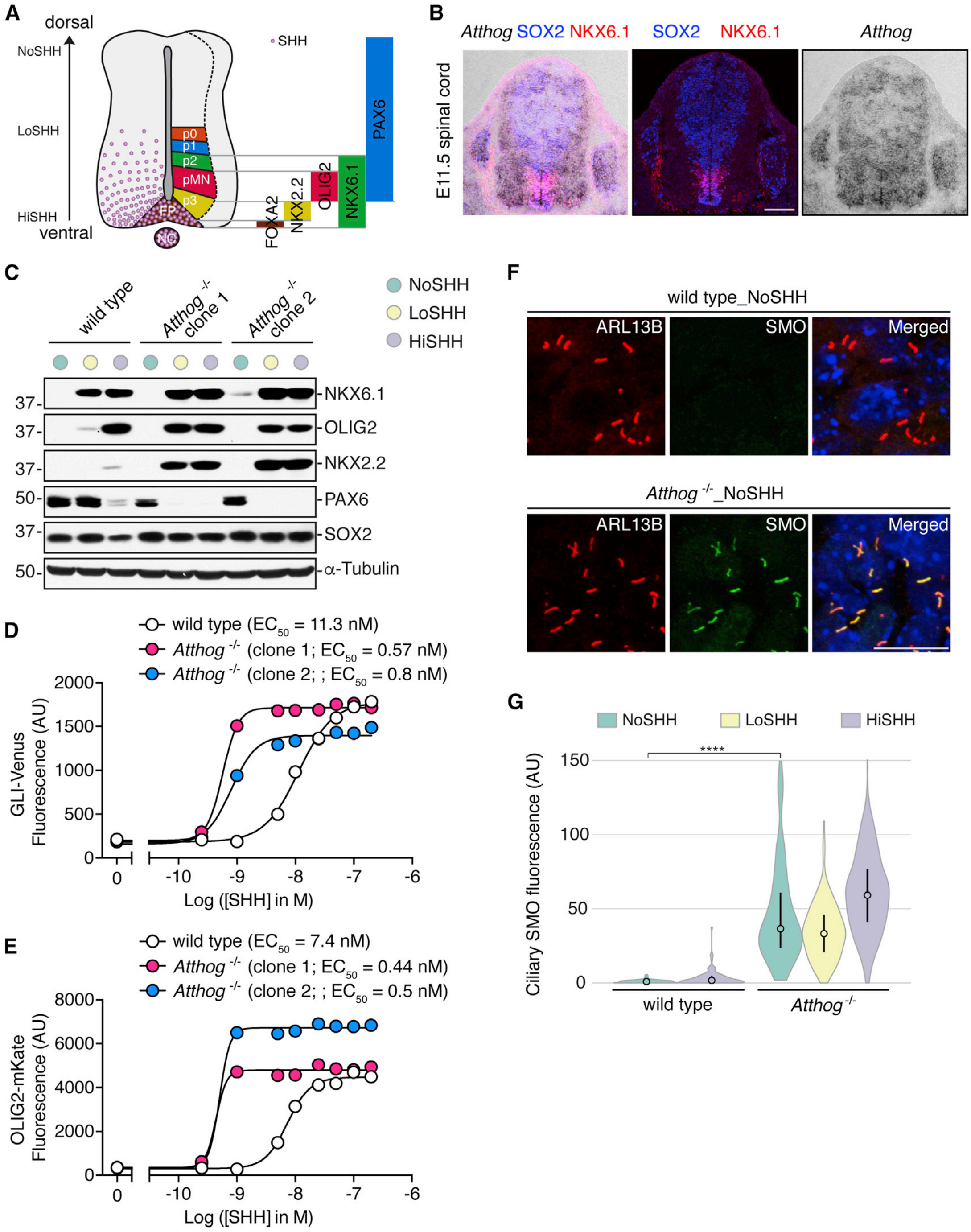

Figure 8. ATTHOG Attenuates SHH-Induced Neural Differentiation Programs

(A) Progenitor domains within the embryonic spinal cord. NC, notochord; FP, floor plate; pMN, motor neuron progenitors; p0, p1, p2, and p3, ventral interneuron progenitors. A SHH gradient (purple circles) along the ventral to dorsal axis establishes progenitor domains that are each defined by the expression of a set of transcription factors (shown on the right).

(B) Distribution of *Atthog* mRNA (by *in situ* hybridization) in a transverse section of E11.5 mouse spinal cord tissue relative to the distribution (by IF) of the Hh-responsive transcription factor NKX6.1 (see A) and the neural progenitor marker SOX2.

(C) Immunoblots to assess the abundance of transcription factors that define progenitor identity after treatment of NPCs with LoSHH or HiSHH. Induction of NKX6.1, OLIG2, and NKX2.2 requires progressively higher doses of SHH, consistent with their expression at increasingly ventral positions in the neural tube; see (A).

(legend continued on next page)

of PTCH1 was unchanged (even though the abundance of PTCH1 was higher due to increased transcription driven by constitutive signaling). These data show that ATTHOG likely promotes SMO degradation after its exit from the ER. The post-ER population includes SMO in the plasma membrane or in various intracellular membranes. To analyze the trafficking of SMO present at the cell surface, we selectively labeled this pool using a cell-impermeable biotinylation reagent (Figure 7C). The stability of cell-surface SMO was much greater in *Atthog*$^{-/-}$ cells (Figures 7D and S7F), likely because of a defect in SMO internalization (Figures 7E and 7F). We conclude that ATTHOG promotes the internalization and subsequent degradation of SMO present at the cell surface. The increased levels of SMO in the ciliary membrane of *Atthog*$^{-/-}$ cells (Figure 5C) are likely to be a consequence of an overall increase in plasma membrane SMO. A microscopy-based assay revealed that the turnover of ciliary SMO was much slower in *Atthog*$^{-/-}$ cells compared with WT cells (Figures 7G and 7H).

Loss of ATTHOG Modifies SHH-Guided Neural Patterning

An intensively studied role of Hh signaling in vertebrates is in patterning of the developing spinal cord, where ventral neural identity is determined by a gradient of SHH secreted by the notochord and floor plate (Cohen et al., 2013; Jessell, 2000) (Figure 8A). This morphogenetic activity of Hh ligands can be recapitulated in spinal cord NPCs. Compared with NIH/3T3 cells, NPCs afford a more physiological readout of Hh signaling strength: the adoption of different cell fates assayed by the expression of TFs that define progenitor identity (Cohen et al., 2013; Gouti et al., 2014). These TFs can be divided into two groups: class I TFs that are expressed in the absence of SHH (PAX6) and class II TFs driven by increasing concentrations of SHH (low, NKX6.1; medium, OLIG2; and high, NKX2.2).

Atthog was expressed in the ventral neural tube of E11.5 mouse embryos, a stage when SHH-induced patterning is operative (Figure 8B). Thus, we asked if ATTHOG could modify the important relationship between the SHH concentration and neural differentiation, a quantitative dose-response relationship that is essential to the proper patterning of the neural tube. In WT NPCs, low concentrations of SHH induced the full expression of NKX6.1 and the low-level expression of OLIG2, which depend on low- and medium-strength signaling respectively (Figure 8C). However, high concentrations of SHH were required to trigger NKX2.2 and to suppress PAX6. In *Atthog*$^{-/-}$ cells, however, low concentrations of SHH were sufficient to drive full expression of NKX2.2 and full suppression of PAX6 (Figure 8C). Thus, the loss of ATTHOG resulted in altered SHH-guided patterning of NPCs: neural progenitor subtypes specified at low concentrations of SHH changed from predominantly NKX6.1^{+} p2 progenitors to NKX2.2^{+} p3 progenitors and OLIG2^{+} motor neuron progenitors. Importantly, the loss of ATTHOG did not change the expression of SOX2, a marker of neural progenitors, showing that the multi-step process of mESC differentiation into NPCs was not influenced by ATTHOG function (Figure 8C).

To measure complete SHH dose-response curves in WT and *Atthog*$^{-/-}$ NPCs, we took advantage of the previously described GLI-Venus and OLIG2-mKate dual-reporter cells (Figures 3C and 3D). The induction of both GLI-Venus and OLIG2-mKate fluorescence was 10-fold more sensitive to SHH in *Atthog*$^{-/-}$ cells compared with WT cells. The SHH dose-response curve was shifted to the left in *Atthog*$^{-/-}$ cells, with the EC_{50} (the concentration required to attain half-maximal levels of GLI-Venus or OLIG2-mKate fluorescence) of SHH decreasing from ~10 nM in WT cells to ~1 nM in *Atthog*$^{-/-}$ cells (Figures 8D and 8E). As we found in NIH/3T3 cells, the loss of ATTHOG led to the high-level, constitutive accumulation of SMO in the primary cilia of NPCs, suggesting that the mechanism behind the increase in target cell sensitivity is likely to be similar in both cell types (Figures 8F and 8G).

In summary, the loss of ATTHOG sensitized both NIH/3T3s and NPCs to Hh ligands, altering both target gene expression and differentiation outcomes.

DISCUSSION

We describe a set of four genome-wide screens that comprehensively identified positive, negative, and attenuating regulators of the Hh pathway. While we only characterized a handful of genes that met extremely stringent selection criteria (Figure S2A), our screens identified many other genes that have statistically significant, quantitative effects on Hh signaling (Table S1). The dataset generated from these screens will serve as a valuable resource for the discovery of genes relevant both to primary cilia and Hh signaling, and consequently to associated diseases such as ciliopathies and other developmental disorders.

This screening strategy, also used in our previous genetic dissection of the WNT pathway (Lebensohn et al., 2016), identified all classes of regulators by using a fluorescent transcriptional reporter that provides a continuous readout of Hh signaling strength. This choice should be contrasted with alternative digital selection schemes based on cell viability, colloquially known as "live/dead" screens. Reporters with continuous readouts, such as fluorescence intensity, allow full control over the stringency of selection applied to the mutant cell library. This flexibility is particularly important when conducting enhancer or suppressor screens in sensitized backgrounds, a powerful and time-honored genetic strategy to elucidate signaling pathways. Indeed, our screen for genes that enhanced signaling in the presence of a low, sub-saturating dose of SHH (Figure 1D) was the most successful in identifying known negative regulators and attenuators compared with screens performed either in the absence of SHH (Figure 1E) or in the presence of saturating SHH (Figure 1C). Similarly, our previous WNT pathway screens identified the attenuator ZNRF3 only when signaling was

(D and E) Activation of the GLI-Venus (D) or OLIG2-mKate (E) reporter in wild-type NPCs and two independent clonal *Atthog*$^{-/-}$ NPC lines treated with increasing concentrations of SHH. Each point represents the median fluorescence from ~10,000 cells. A representative dose curve from three independent experiments is shown.

(F and G) Representative micrographs (F) and corresponding violin plots (G, n ~ 100 cilia per condition) showing levels of endogenous SMO at cilia of WT and *Atthog*$^{-/-}$ NPCs. Scale bars, 100 μm in (B) or 10 μm in (F). Significance was determined by the Kruskal-Wallis test; ****$p < 0.0001$.

activated with low ligand concentrations (Lebensohn et al., 2016). We propose that varying the ligand concentration provides an easy but powerful method to sensitize screens in cultured cells and thereby reveal hidden layers of regulation that tune signaling strength. This may be particularly relevant for graded signaling pathways initiated by morphogens such as WNT and Hh that can engage distinct differentiation programs at varying concentrations. The screening platform described here should be broadly applicable to uncover genes regulating any cellular process that can be monitored using a fluorescence-based reporter that allows for FACS-based enrichment of cells with the desired phenotype.

In addition to the identification of most components of the Hh pathway and the identification of many genes involved in cilia and ciliopathies, our genetic analysis uncovered a hidden layer of regulation in vertebrate Hh signaling. Three of the top signaling attenuators, *Megf8*, *Mgrn1*, and *Atthog*, all suppressed SMO protein levels at the cell surface and cilium, likely by regulating its endocytosis from the plasma membrane. The loss of these genes led to a ~10-fold increase in sensitivity to SHH and, consequently, alterations in SHH-induced NPC differentiation outcomes. The most straightforward explanation for this potentiation effect is that high ciliary SMO protein levels overwhelm the inhibitory capacity of PTCH1.

While *Atthog* was a formerly uncharacterized gene, both *Megf8* and *Mgrn1* have been studied previously and phenotypes of animals carrying mutations in these genes have been reported (Cota et al., 2006; Engelhard et al., 2013; He et al., 2003; Phan et al., 2002; Zhang et al., 2009). Does this prior analysis support a role for these genes in the Hh pathway as suggested by our screens? Forward genetic screens in mice demonstrated that *Megf8* mutations cause embryonic lethality with multi-organ defects (Engelhard et al., 2013; Zhang et al., 2009). *Megf8* was proposed to be a modifier of BMP signaling due to phenotypic similarities with BMP loss-of-function mice, but this correlation was not tested using direct signaling assays (Engelhard et al., 2013). Many phenotypes seen in *Megf8*$^{-/-}$ mice, such as polydactyly, cardiac development defects, exencephaly, and heterotaxy, are also associated with Hh signaling defects.

Human genetics supports a role of *MEGF8* as a Hh pathway modifier. Mutations in *MEGF8* cause a subtype of Carpenter's syndrome associated with heterotaxy, congenital cardiac defects, pre-axial polydactyly, and skeletal and craniofacial defects (Twigg et al., 2012). Some of the skeletal phenotypes seen in these patients, such as hypertelorism and polydactyly, are associated with increased Hh signaling. Strikingly, most individuals with Carpenter's syndrome have mutations in *RAB23* (Jenkins et al., 2007), which encodes a previously described negative regulator of Hh signaling that was also identified as a top hit in our LoSHH_Top5% screen (Figure 1D; Eggenschwiler et al., 2001). Thus, consistent with our results, the loss of MEGF8 resembles the loss of other known negative regulators of the Hh pathway in both mice and humans.

Mice carrying mutations in *Mgrn1* display pigmentation defects and progressive spongiform degeneration, phenotypes not typically associated with Hh signaling (He et al., 2003; Phan et al., 2002). However, *Mgrn1*$^{-/-}$ embryos have a 40%–60% incidence of embryonic lethality, which has only been partially characterized. A subset of these embryos (~25%) display heterotaxy and complex cardiac anomalies, phenotypes that overlap with *Megf8*$^{-/-}$ mice (Cota et al., 2006). Both phenotypes can be associated with impaired ciliary function or Hh signaling. The lack of a strong embryonic Hh phenotype in these mice may be because *Mgrn1* is redundant with *Rnf157* in vertebrates (Figure S4B).

The common influence on SMO stability and the common signaling phenotype points to the possibility that *Megf8*, *Mgrn1*, and *Atthog* function in the same pathway. In fact, MGRN1 and MEGF8 are annotated as interaction partners in the high-quality BioPlex protein interaction database (http://bioplex.hms.harvard.edu/). MGRN1 has been shown to function with ATTRACTIN (ATRN), a paralog of MEGF8, to downregulate the MC4R melanocortin receptor (Overton and Leibel, 2011). Loss-of-function mutations in either *Mgrn1* or *Atrn* lead to elevated cell-surface levels of MC4R, analogous to our observation that disruption of *Mgrn1* or *Megf8* lead to increased cell-surface and ciliary levels of SMO. Hence, the common biochemical function of MEGF8, ATRN, and other related proteins may be to target selected GPCRs for ubiquitination and downregulation by MGRN1 and related RING-finger cytoplasmic E3 ligases.

We end by noting that the post-translational mechanism of SMO regulation uncovered by our screens is conceptually analogous to the intensively studied mechanism that attenuates WNT signaling by decreasing the cell-surface levels of Frizzled (FZD) proteins, receptors for WNT ligands that are the closest relatives of SMO in the GPCR superfamily. In vertebrates, two transmembrane E3 ubiquitin ligases, ZNRF3 and RNF43, dampen sensitivity to WNT ligands by reducing cell-surface levels of FZD receptors (reviewed in de Lau et al., 2014). Ligands of the R-Spondin family bind and neutralize ZNRF3/RNF43 with the assistance of LGR co-receptors, allowing FZD levels and WNT sensitivity to dramatically increase in specific tissues during development and in specific stem cell compartments in adults. Future work will investigate whether *Megf8*, *Mgrn1*, or *Atthog* are regulated either by Hh ligands themselves or by other signals to potentiate or attenuate Hh signaling.

STAR★METHODS

Detailed methods are provided in the online version of this paper and include the following:

- KEY RESOURCES TABLE
- CONTACT FOR REAGENT AND RESOURCE SHARING
- EXPERIMENTAL MODEL AND SUBJECT DETAILS
  - Cell Culture
  - Neural Progenitor Differentiation Assays
- METHOD DETAILS
  - Constructs
  - Reagents and Antibodies
  - Ligand and Small Molecule Inhibitor Treatment Protocols
  - Pooled Genome-wide CRISPR/Cas9 Screens
  - Knockout of Candidate Genes in Pooled Cell Lines
  - Clonal Cell Lines Carrying Deletions in Candidate Genes
  - Real-Time Quantitative Reverse Transcription PCR (Real-Time qRT-PCR)

- ○ Western Blotting
- ○ SMO Trafficking Assays
- ○ *In Situ* Hybridization
- ○ Analysis of Cilia Protein Localization by Immunofluorescence
- ○ Protein Sequence Analysis, Domain Identification, and Phylogenetic Analysis
- ○ Gene Ontology (GO) Analysis
- QUANTIFICATION AND STATISTICAL ANALYSIS
- DATA AND SOFTWARE AVAILABILITY

SUPPLEMENTAL INFORMATION

Supplemental Information includes seven figures and three tables and can be found with this article online at https://doi.org/10.1016/j.devcel.2017.12.003.

ACKNOWLEDGMENTS

We thank Deborah Keller and Mina Gouti for providing the GBS-Venus mouse ESC line, Ramin Dubey for providing pSpCas9(BB)-2A-mCherry plasmid, and all Rohatgi lab members for input on the project. We are grateful to Andreas Puschnik and Wipapat Kladwang for help with NGS. Work in R.R.'s laboratory was funded by NIH grants GM105448, GM118082, and GM112988. A.K. and L.A. were supported by the Intramural Research Program of the National Library of Medicine, NIH, USA. J.B.'s lab was supported by the Francis Crick Institute, which receives its funding from Cancer Research UK (FC001051), the UK Medical Research Council (FC001051), and the Wellcome Trust (FC001051; WT098326MA). G.V.P. was supported by a postdoctoral fellowship from the American Heart Association; J.H.K. by the Stanford School of Medicine Dean's postdoctoral fellowship and Jump Start Award; M.K. by the NSF Graduate Research Fellowship Program; and A.S. by the EMBO LTF (1438-2013), HFSP LTF (LT000401/2014-L), and the People Program (Marie Curie Actions) of the European Union's Seventh Framework Program FP7-2013 under REA grant agreement no. 624973.

AUTHOR CONTRIBUTIONS

R.R. and G.V.P. conceptualized the project; G.V.P., J.H.K., and B.B.P. designed, performed, and analyzed all the experiments; M.K. and B.B.P. developed scripts for image analysis; A.S. and J.B. contributed the dual-reporter NPC cell line and NPC methods; A.K. and L.A. performed the sequence and phylogenetic analyses; all authors contributed to the writing and editing of the manuscript.

DECLARATION OF INTERESTS

The authors declare no competing interests.

Received: October 2, 2017
Revised: November 20, 2017
Accepted: December 1, 2017
Published: December 28, 2017

REFERENCES

Altschul, S.F., Madden, T.L., Schäffer, A.A., Zhang, J., Zhang, Z., Miller, W., and Lipman, D.J. (1997). Gapped BLAST and PSI-BLAST: a new generation of protein database search programs. Nucleic Acids Res. *25*, 3389–3402.

Bangs, F., and Anderson, K.V. (2016). Primary cilia and mammalian hedgehog signaling. Cold Spring Harb Perspect. Biol. *9*, a028175.

Bishop, B., Aricescu, A.R., Harlos, K., O'Callaghan, C.A., Jones, E.Y., and Siebold, C. (2009). Structural insights into hedgehog ligand sequestration by the human hedgehog-interacting protein HHIP. Nat. Struct. Mol. Biol. *16*, 698–703.

Briscoe, J., and Thérond, P.P. (2013). The mechanisms of Hedgehog signalling and its roles in development and disease. Nat. Rev. Mol. Cell Biol. *14*, 416–429.

Cohen, M., Briscoe, J., and Blassberg, R. (2013). Morphogen interpretation: the transcriptional logic of neural tube patterning. Curr. Opin. Genet. Dev. *23*, 423–428.

Cota, C.D., Bagher, P., Pelc, P., Smith, C.O., Bodner, C.R., and Gunn, T.M. (2006). Mice with mutations in Mahogunin ring finger-1 (Mgrn1) exhibit abnormal patterning of the left-right axis. Dev. Dyn. *235*, 3438–3447.

Cuff, J.A., Clamp, M.E., Siddiqui, A.S., Finlay, M., and Barton, G.J. (1998). JPred: a consensus secondary structure prediction server. Bioinformatics *14*, 892–893.

de Lau, W., Peng, W.C., Gros, P., and Clevers, H. (2014). The R-spondin/Lgr5/Rnf43 module: regulator of Wnt signal strength. Genes Dev. *28*, 305–316.

Dessaud, E., McMahon, A.P., and Briscoe, J. (2008). Pattern formation in the vertebrate neural tube: a sonic hedgehog morphogen-regulated transcriptional network. Development *135*, 2489–2503.

Dickinson, M.E., Flenniken, A.M., Ji, X., Teboul, L., Wong, M.D., White, J.K., Meehan, T.F., Weninger, W.J., Westerberg, H., Adissu, H., et al. (2016). High-throughput discovery of novel developmental phenotypes. Nature *537*, 508–514.

Doench, J.G., Fusi, N., Sullender, M., Hegde, M., Vaimberg, E.W., Donovan, K.F., Smith, I., Tothova, Z., Wilen, C., Orchard, R., et al. (2016). Optimized sgRNA design to maximize activity and minimize off-target effects of CRISPR-Cas9. Nat. Biotechnol. *34*, 184–191.

Dorn, K.V., Hughes, C.E., and Rohatgi, R. (2012). A smoothened-Evc2 complex transduces the hedgehog signal at primary cilia. Dev. Cell *23*, 823–835.

Dutcher, S.K., and Trabuco, E.C. (1998). The UNI3 gene is required for assembly of basal bodies of *Chlamydomonas* and encodes delta-tubulin, a new member of the tubulin superfamily. Mol. Biol. Cell *9*, 1293–1308.

Eggenschwiler, J.T., Espinoza, E., and Anderson, K.V. (2001). Rab23 is an essential negative regulator of the mouse Sonic hedgehog signalling pathway. Nature *412*, 194–198.

Engelhard, C., Sarsfield, S., Merte, J., Wang, Q., Li, P., Beppu, H., Kolodkin, A.L., Sucov, H.M., and Ginty, D.D. (2013). MEGF8 is a modifier of BMP signaling in trigeminal sensory neurons. Elife *2*, e01160.

Gouti, M., Tsakiridis, A., Wymeersch, F.J., Huang, Y., Kleinjung, J., Wilson, V., and Briscoe, J. (2014). In vitro generation of neuromesodermal progenitors reveals distinct roles for Wnt signalling in the specification of spinal cord and paraxial mesoderm identity. PLoS Biol. *12*, e1001937.

He, L., Lu, X.-Y., Jolly, A.F., Eldridge, A.G., Watson, S.J., Jackson, P.K., Barsh, G.S., and Gunn, T.M. (2003). Spongiform degeneration in mahoganoid mutant mice. Science *299*, 710–712.

Huang, D.W., Sherman, B.T., and Lempicki, R.A. (2008). Systematic and integrative analysis of large gene lists using DAVID bioinformatics resources. Nat. Protoc. *4*, 44–57.

Huangfu, D., and Anderson, K.V. (2005). Cilia and Hedgehog responsiveness in the mouse. Proc. Natl. Acad. Sci. USA *102*, 11325–11330.

Huangfu, D., Liu, A., Rakeman, A.S., Murcia, N.S., Niswander, L., and Anderson, K.V. (2003). Hedgehog signalling in the mouse requires intraflagellar transport proteins. Nature *426*, 83–87.

Humke, E.W., Dorn, K.V., Milenkovic, L., Scott, M.P., and Rohatgi, R. (2010). The output of Hedgehog signaling is controlled by the dynamic association between Suppressor of Fused and the Gli proteins. Genes Dev. *24*, 670–682.

Jenkins, D., Seelow, D., Jehee, F.S., Perlyn, C.A., Alonso, L.G., Bueno, D.F., Donnai, D., Josifiova, D., Mathijssen, I.M.J., Morton, J.E.V., et al. (2007). RAB23 mutations in carpenter syndrome imply an unexpected role for hedgehog signaling in cranial-suture development and obesity. Am. J. Hum. Genet. *80*, 1162–1170.

Jessell, T.M. (2000). Neuronal specification in the spinal cord: inductive signals and transcriptional codes. Nat. Rev. Genet. *1*, 20–29.

Joung, J., Konermann, S., Gootenberg, J.S., Abudayyeh, O.O., Platt, R.J., Brigham, M.D., Sanjana, N.E., and Zhang, F. (2017). Genome-scale CRISPR-Cas9 knockout and transcriptional activation screening. Nat. Protoc. *12*, 828–863.

Kuleshov, M.V., Jones, M.R., Rouillard, A.D., Fernandez, N.F., Duan, Q., Wang, Z., Koplev, S., Jenkins, S.L., Jagodnik, K.M., Lachmann, A., et al.

(2016). Enrichr: a comprehensive gene set enrichment analysis web server 2016 update. Nucl. Acids Res. *44*, W90–W97.

Lassmann, T., Frings, O., and Sonnhammer, E.L.L. (2009). Kalign2: high-performance multiple alignment of protein and nucleotide sequences allowing external features. Nucl. Acids Res. *37*, 858–865.

Lebensohn, A.M., Dubey, R., Neitzel, L.R., Tacchelly-Benites, O., Yang, E., Marceau, C.D., Davis, E.M., Patel, B.B., Bahrami-Nejad, Z., Travaglini, K.J., et al. (2016). Comparative genetic screens in human cells reveal new regulatory mechanisms in WNT signaling. Elife *5*, 410.

Li, W., Xu, H., Xiao, T., Cong, L., Love, M.I., Zhang, F., Irizarry, R.A., Liu, J.S., Brown, M., and Liu, X.S. (2014). MAGeCK enables robust identification of essential genes from genome-scale CRISPR/Cas9 knockout screens. Genome Biol. *15*, 554.

Lukov, G.L., Hu, T., McLaughlin, J.N., Hamm, H.E., and Willardson, B.M. (2005). Phosducin-like protein acts as a molecular chaperone for G protein $\beta\gamma$ dimer assembly. EMBO J. *24*, 1965–1975.

Milenkovic, L., Scott, M.P., and Rohatgi, R. (2009). Lateral transport of Smoothened from the plasma membrane to the membrane of the cilium. J. Cell Biol. *187*, 365–374.

Neuwald, A.F., and Altschul, S.F. (2016). Bayesian top-down protein sequence alignment with inferred position-specific gap penalties. PLOS Comput. Biol. *12*, e1004936.

Niewiadomski, P., Kong, J.H., Ahrends, R., Ma, Y., Humke, E.W., Khan, S., Teruel, M.N., Novitch, B.G., and Rohatgi, R. (2014). Gli protein activity is controlled by multisite phosphorylation in vertebrate hedgehog signaling. Cell Rep. *6*, 168–181.

Novitch, B.G., Wichterle, H., Jessell, T.M., and Sockanathan, S. (2003). A requirement for retinoic acid-mediated transcriptional activation in ventral neural patterning and motor neuron specification. Neuron *40*, 81–95.

Nüsslein-Volhard, C., and Wieschaus, E. (1980). Mutations affecting segment number and polarity in *Drosophila*. Nature *287*, 795–801.

Overton, J.D., and Leibel, R.L. (2011). Mahoganoid and mahogany mutations rectify the obesity of the yellow mouse by effects on endosomal traffic of MC4R protein. J. Biol. Chem. *286*, 18914–18929.

Parnas, O., Jovanovic, M., Eisenhaure, T.M., Herbst, R.H., Dixit, A., Ye, C.J., Przybylski, D., Platt, R.J., Tirosh, I., Sanjana, N.E., et al. (2015). A genome-wide CRISPR screen in primary immune cells to dissect regulatory networks. Cell *162*, 675–686.

Phan, L.K., Lin, F., LeDuc, C.A., Chung, W.K., and Leibel, R.L. (2002). The mouse mahoganoid coat color mutation disrupts a novel C3HC4 RING domain protein. J. Clin. Invest *110*, 1449–1459.

Phua, S.C., Chiba, S., Suzuki, M., Su, E., Roberson, E.C., Pusapati, G.V., Setou, M., Rohatgi, R., Reiter, J.F., Ikegami, K., et al. (2017). Dynamic remodeling of membrane composition drives cell cycle through primary cilia excision. Cell *168*, 264–279.e15.

Pletscher-Frankild, S., Pallejà, A., Tsafou, K., Binder, J.X., and Jensen, L.J. (2015). DISEASES: text mining and data integration of disease–gene associations. Methods *74*, 83–89.

Price, M.N., Dehal, P.S., and Arkin, A.P. (2009). FastTree: computing large minimum evolution trees with profiles instead of a distance matrix. Mol. Biol. Evol. *26*, 1641–1650.

Punta, M., Coggill, P.C., Eberhardt, R.Y., Mistry, J., Tate, J., Boursnell, C., Pang, N., Forslund, K., Ceric, G., Clements, J., et al. (2012). The Pfam protein families database. Nucl. Acids Res. *40*, D290–D301.

Pusapati, G.V., Hughes, C.E., Dorn, K.V., Zhang, D., Sugianto, P., Aravind, L., and Rohatgi, R. (2014). EFCAB7 and IQCE regulate hedgehog signaling by tethering the EVC-EVC2 complex to the base of primary cilia. Dev. Cell *28*, 483–496.

Ran, F.A., Hsu, P.D., Wright, J., Agarwala, V., Scott, D.A., and Zhang, F. (2013). Genome engineering using the CRISPR-Cas9 system. Nat. Protoc. *8*, 2281–2308.

Reiter, J.F., and Leroux, M.R. (2017). Genes and molecular pathways underpinning ciliopathies. Nat. Rev. Mol. Cell Biol. *18*, 533–547.

Rohatgi, R., Milenkovic, L., and Scott, M.P. (2007). Patched1 regulates hedgehog signaling at the primary cilium. Science *317*, 372–376.

Sanjana, N.E., Shalem, O., and Zhang, F. (2014). Improved vectors and genome-wide libraries for CRISPR screening. Nat. Methods *11*, 783–784.

Schindelin, J., Arganda-Carreras, I., Frise, E., Kaynig, V., Longair, M., Pietzsch, T., Preibisch, S., Rueden, C., Saalfeld, S., Schmid, B., et al. (2012). Fiji: an open-source platform for biological-image analysis. Nat. Methods *9*, 676–682.

Twigg, S.R.F., Lloyd, D., Jenkins, D., Elçioglu, N.E., Cooper, C.D.O., Al-Sannaa, N., Annagür, A., Gillessen-Kaesbach, G., Hüning, I., Knight, S.J.L., et al. (2012). Mutations in multidomain protein MEGF8 identify a carpenter syndrome subtype associated with defective lateralization. Am. J. Hum. Genet. *91*, 897–905.

van Dam, T.J., Wheway, G., Slaats, G.G., Huynen, M.A., and Giles, R.H. (2013). The SYSCILIA gold standard (SCGSv1) of known ciliary components and its applications within a systems biology consortium. Cilia *2*, 7.

Vokes, S.A., Ji, H., McCuine, S., Tenzen, T., Giles, S., Zhong, S., Longabaugh, W.J.R., Davidson, E.H., Wong, W.H., and McMahon, A.P. (2007). Genomic characterization of Gli-activator targets in sonic hedgehog-mediated neural patterning. Development *134*, 1977–1989.

Wang, J.T., Kong, D., Hoerner, C.R., Loncarek, J., and Stearns, T. (2017). Centriole triplet microtubules are required for stable centriole formation and inheritance in human cells. Elife *6*, e29061.

Wang, T., and Hong, W. (2002). Interorganellar regulation of lysosome positioning by the Golgi apparatus through Rab34 interaction with Rab-interacting lysosomal protein. Mol. Biol. Cell *13*, 4317–4332.

Xiong, W., Grillet, N., Elledge, H.M., Wagner, T.F.J., Zhao, B., Johnson, K.R., Kazmierczak, P., and Müller, U. (2012). TMHS is an integral component of the mechanotransduction machinery of cochlear hair cells. Cell *151*, 1283–1295.

Zhang, Z., Alpert, D., Francis, R., Chatterjee, B., Yu, Q., Tansey, T., Sabol, S.L., Cui, C., Bai, Y., Koriabine, M., et al. (2009). Massively parallel sequencing identifies the gene Megf8 with ENU-induced mutation causing heterotaxy. Proc. Natl. Acad. Sci. USA *106*, 3219–3224.

STAR★METHODS

KEY RESOURCES TABLE

REAGENT or RESOURCE	SOURCE	IDENTIFIER
Antibodies		
Mouse monoclonal anti-GLI1 (clone L42B10)	Cell Signaling	Cat#2643; RRID: AB_2294746
Goat polyclonal anti-GLI3	R and D Systems	Cat#AF3690; RRID: AB_2232499
Rabbit polyclonal anti-p38	Abcam	Cat#ab7952; RRID: AB_306166
Mouse monoclonal anti-1D4	The University of British Columbia	RRID: AB_325050
Mouse monoclonal anti-Alpha Tubulin (clone DM1A)	Sigma-Aldrich	Cat#T6199; RRID: AB_477583
Mouse monoclonal anti-Acetylated Tubulin	Sigma-Aldrich	Cat#T6793; RRID: AB_477585
Mouse monoclonal anti-NKX2.2	DSHB	Cat#74.5A5; RRID: AB_531794
Mouse monoclonal anti-NKX6.1	DSHB	Cat#F55A10; RRID: AB_532378
Rabbit polyclonal anti-PAX6	EMD Millipore	Cat# AB2237; RRID: AB_1587367
Alexa Fluor 488 conjugated anti-Giantin	Covance	Cat# A488-114L; RRID: AB_389880
Rabbit polyclonal anti-PTCH1	Rohatgi et al., 2007	N/A
Rabbit polyclonal anti-SMO	Rohatgi et al., 2007 and Milenkovic et al., 2009	N/A
Rabbit polyclonal anti-SUFU	Humke et al., 2010	N/A
Guinea pig polyclonal anti-OLIG2	Novitch et al., 2003	N/A
Rabbit polyclonal anti-IQCE	Pusapati et al., 2014	N/A
Guinea pig polyclonal anti-GLI2	Niewiadomski et al., 2014	N/A
Guinea pig polyclonal anti-ARL13B	Dorn et al., 2012	N/A
Donkey anti-Rabbit IgG (H+L) Highly Cross-Adsorbed Secondary Antibody, Alexa Fluor 488	Thermo Fisher Scientific	Cat#A-21206; RRID: AB_2535792
Alexa Fluor® 488 AffiniPure Donkey Anti-Guinea Pig IgG (H+L)	Jackson ImmunoResearch Laboratories	Cat#706-545-148; RRID: AB_2340472
Donkey anti-Rabbit IgG (H+L) Highly Cross-Adsorbed Secondary Antibody, Alexa Fluor 594	Thermo Fisher Scientific	Cat#A-21207; RRID: AB_141637
Donkey anti-Mouse IgG (H+L) Secondary Antibody, Alexa Fluor 647	Thermo Fisher Scientific	Cat#A-31571; RRID: AB_162542
Alexa Fluor® 647 AffiniPure Donkey Anti-Guinea Pig IgG (H+L)	Jackson ImmunoResearch Laboratories	Cat#706-605-148; RRID: AB_2340476
Peroxidase AffiniPure Donkey Anti-Mouse IgG (H+L)	Jackson ImmunoResearch Laboratories	Cat#715-035-150; RRID: AB_2340770
Peroxidase AffiniPure Donkey Anti-Rabbit IgG (H+L)	Jackson ImmunoResearch Laboratories	Cat#111-035-144; RRID: AB_2307391
Peroxidase AffiniPure Donkey Anti-Goat IgG (H+L)	Jackson ImmunoResearch Laboratories	Cat#705-035-003; RRID: AB_2340390
Chemicals, Peptides, and Recombinant Proteins		
Sonic hedgehog	Bishop et al., 2009	N/A
WNT3A	R&D Systems	Cat#1324-WN-002
BMP4	Thermo Fisher Scientific	Cat#PHC9534
Bafilomycin A1	LC Labs	Cat#B-1080
Leupeptin	Sigma-Aldrich	Cat#L2884
Vismodegib	LC Labs	Cat#V-4050
Polybrene	Sigma-Aldrich	Cat#H9268
Puromycin	Sigma-Aldrich	Cat#P8833
Blasticidin	Thermo Fisher Scientific	Cat#R210-01
bFGF	R and D Systems	Cat#3139-FB-025
Retinoic Acid	Sigma-Aldrich	Cat#R2625

(*Continued on next page*)

Continued

REAGENT or RESOURCE	SOURCE	IDENTIFIER
CHIR99021	Axon Medchem LLC	Cat#Axon 1386
PD 98059	Axon Medchem LLC	Cat#Axon 1223
ESGRO LIF	EMD Millipore	Cat#ESG1106
Polyethylenimine	Polysciences, Inc	Cat#23966-1
Anti-DIG alkaline phosphatase Fab fragments	Roche	Cat#11093274910
NBT/BCIP	Roche	Cat#11681451001
Critical Commercial Assays		
X-tremeGENE 9	Roche	Cat#06366236001
Mouse ES Cell Nucleofector Kit	Lonza	Cat#VAPH-1001
Power SYBR Green Cells-to-CT Kit	Thermo Fisher Scientific	Cat#4402955
MiSeq Reagent Kit v3	Illumina	Cat#MS-102-3001
DIG RNA Labeling Kit	Roche	Cat#11175025910
Deposited Data		
All Fastq files from NGS	NIH Short Read Archive (SRA)	SRP116669
Experimental Models: Cell Lines		
NIH/3T3-Flp In	Thermo Fisher Scientific	Cat#R76107
293FT	Thermo Fisher Scientific	Cat#R70007
NIH/3T3-Flp In *Cep350*$^{-/-}$	This paper	N/A
NIH/3T3-Flp In *Rab34*$^{-/-}$	This paper	N/A
NIH/3T3-Flp In *Fkbp10*$^{-/-}$	This paper	N/A
NIH/3T3-Flp In *Tubd1*$^{-/-}$	This paper	N/A
NIH/3T3-Flp In *Pdcl*$^{-/-}$	This paper	N/A
NIH/3T3-Flp In *Atthog*$^{-/-}$	This paper	N/A
NIH/3T3-Flp In *Ccm2*$^{-/-}$	This paper	N/A
NIH/3T3-Flp In *Megf8*$^{-/-}$	This paper	N/A
NIH/3T3-Flp In *Mesdc1*$^{-/-}$	This paper	N/A
NIH/3T3-Flp In *Pdcd10*$^{-/-}$	This paper	N/A
NIH/3T3-Flp In *Mgrn1*$^{-/-}$	This paper	N/A
NIH/3T3-Flp In *Ptch1*$^{-/-}$	This paper	N/A
NIH/3T3-Flp In *Sufu*$^{-/-}$	This paper	N/A
HM1 mESC GBS-Venus and Olig2-mKate	doi: https://doi.org/10.1101/104307	N/A
HM1 mESC *Cep350*$^{-/-}$	This paper	N/A
HM1 mESC *Rab34*$^{-/-}$	This paper	N/A
HM1 mESC *Tubd1*$^{-/-}$	This paper	N/A
HM1 mESC *Pdcl*$^{-/-}$	This paper	N/A
HM1 mESC *Atthog*$^{-/-}$	This paper	N/A
HM1 mESC *Ccm2*$^{-/-}$	This paper	N/A
HM1 mESC *Megf8*$^{-/-}$	This paper	N/A
HM1 mESC *Mesdc1*$^{-/-}$	This paper	N/A
HM1 mESC *Pdcd10*$^{-/-}$	This paper	N/A
HM1 mESC *Mgrn1*$^{-/-}$	This paper	N/A
Oligonucleotides		
NGS-1st PCR Fwd: 5'-AATGGACTATCATATGCTTACC GTAACTTGAAAGTATTTCG-3'	This paper	N/A
NGS-1st PCR Rev: 5'-CTCGGTGCCACTTTTTCAAGTT GATAACGG-3'	This paper	N/A
Recombinant DNA		
pEF5/FRT/V5-DEST-*Atthog*-1D4	This paper	N/A
Brie mouse CRISPR knockout pooled library	Doench et al., 2016	Addgene #73633

(*Continued on next page*)

Continued		
REAGENT or RESOURCE	SOURCE	IDENTIFIER
lentiCas9-Blast	Sanjana et al., 2014	Addgene #52962
lentiCRISPR v2	Sanjana et al., 2014	Addgene #52961
pMD2.G	Didier Trono Lab, EPFL, Switzerland	Addgene #12259
psPAX2	Didier Trono Lab, EPFL, Switzerland	Addgene #12260
pSpCas9(BB)-2A-GFP (PX458)	Ran et al., 2013	Addgene #48138
pSpCas9(BB)-2A-mCherry	This paper	N/A
pSpCas9(BB)-2A-Puro (PX459)	Ran et al., 2013	Addgene #48139
Software and Algorithms		
CRISPR Design	Feng Zhang Lab, MIT, USA	http://crispr.mit.edu
MAGeCK computational tool	Li et al., 2014	https://sourceforge.net/p/mageck/wiki/Home/
Leica Application Suite X	Leica Microsystems	http://www.leica-microsystems.com/products/microscope-software/details/product/leica-las-x-ls/
Fiji	Schindelin et al., 2012	https://fiji.sc
MATLAB R2014a	MathWorks	https://www.mathworks.com
GraphPad Prism version 6	GraphPad Software	https://www.graphpad.com/scientific-software/prism/
R (version 3.3.2)	The R Foundation for Statistical Computing	https://www.r-project.org
Adobe Photoshop CS6	Adobe Systems	http://www.adobe.com/products/photoshop.html
Adobe Illustrator CS6	Adobe Systems	http://www.adobe.com/products/illustrator.html

CONTACT FOR REAGENT AND RESOURCE SHARING

Further information and requests for resources and reagents should be directed to and will be fulfilled by the Lead Contact, Rajat Rohatgi (rrohatgi@stanford.edu).

EXPERIMENTAL MODEL AND SUBJECT DETAILS

Cell Culture

Flp-In-3T3 (a derivative of NIH/3T3 cells) and 293FT cell lines were purchased from Thermo Fisher Scientific and used without further authentication. Information on the gender of cell lines is not available. A Flp-In-3T3 stable cell line expressing tagged *Atthog* was generated as previously described (Pusapati et al., 2014). NIH/3T3 cells expressing GLI-GFP and Cas9 were generated by transduction of the previously described NIH/3T3-GLI-GFP line (Phua et al., 2017) with lentiCas9-Blast (Addgene #52962; (Sanjana et al., 2014)), followed by selection with Blasticidin (2 μg/ml). Single cells were sorted using FACSAria II (BD) and multiple clones were analyzed for optimal Cas9 expression and SHH induced GFP reporter fluorescence. A clonal cell line (NIH3T3-CG) that displayed robust on-target genome editing activity with multiple positive control sgRNAs and also displayed the widest dynamic range for SHH-induced GFP reporter fluorescence was chosen for further studies (Figure S1). All the above mentioned cell lines were cultured in Dulbecco's Modified Eagle Medium (DMEM) containing high glucose (Thermo Scientific) and supplemented with 10% fetal bovine serum (FBS) (Atlanta Biologicals), 1 mM sodium pyruvate (Gibco), 2 mM L-Glutamine (Gemini Biosciences), 1x MEM non-essential amino acids solution (Gibco), penicillin (40 U/ml) and streptomycin (40 μg/ml) (Gemini Biosciences), in a humidified atmosphere containing 5% CO_2 at 37°C. Maintenance of mESCs and their differentiation into NPCs is described below. Stocks of cell lines and derivatives were free of mycoplasma contamination.

To initiate Hh signaling, NIH/3T3 cells were first ciliated by growth to confluence in DMEM containing 10% FBS followed by serum starvation in DMEM containing 0.5% FBS for 24 h. A detailed description of all treatment protocols are included in the "Ligand and small molecule inhibitor treatment protocols" section.

Neural Progenitor Differentiation Assays

The construction and use of the HM1 mESC line harboring the GLI-Venus and OLIG2-mKate dual reporter system to evaluate Hh signaling has been described previously in detail (doi: https://doi.org/10.1101/104307) (Gouti et al., 2014). The parental HM1 mESC line is derived from a male mouse. For mESC maintenance, feeders were plated onto dishes coated with 0.1% gelatin (Sigma-Aldrich) overnight. ESCs were cultured with feeders in mESC media (DMEM containing high glucose, 15% Optima FBS (Atlanta Biologicals), 1x MEM non-essential amino acids, 1% penicillin/streptomycin, 2 mM L-glutamine, 1% EmbryoMax nucleosides (Millipore), 55 μM 2-mercaptoethanol (Gibco), and 1000 U/ml ESGRO LIF (Millipore)). mESCs were differentiated into spinal neural progenitors using a previously described protocol with minor modifications (Gouti et al., 2014). Briefly, mESCs were cleared from feeder cells and plated onto either gelatin coated glass coverslips (12mm diameter, placed in a 24-well plate) at a density of 50,000 cells/coverslip (immunofluorescence staining) or onto gelatin coated CellBIND plates (Corning) at a density of 100,000 cells/6-well (FACS) or 500,000 cells/10 cm plate (Western Blotting). Differentiations were conducted in N2B27 media (Dulbecco's Modified Eagle's Medium F12 (Gibco) and Neurobasal Medium (Gibco) (1:1 ratio) supplemented with N-2 Supplement (Gibco), B-27 Supplement (Gibco), 1% penicillin/streptomycin (Gemini Bio-Products), 2mM L-glutamine (Gemini Bio-Products), 40 μg/ml Bovine Serum Albumin (Sigma), and 55 μM 2-mercaptoethanol (Gibco)). On the day the cells were plated (Day 0) and Day 1, the N2B27 medium was supplemented with 10 ng/ml bFGF (R&D). On Day 2, 10 ng/ml bFGF (R&D) and 5 μM CHIR99021 (Axon) were added to the N2B27 culture medium. On Day 3, cells were cultured in N2B27 medium containing RA (100 nM, Sigma-Aldrich) or RA with different doses of SHH. A fresh medium change with the same ingredients was done on Day 4 and Day 5. On Day 6, cells were rinsed with PBS and either fixed with 4% PFA (immunofluorescence staining), trypsinized (FACS analysis), or lysed for Western Blot analysis.

METHOD DETAILS

Constructs

Mouse *Atthog* was tagged with a C-terminal 1D4 and cloned into pEF5/FRT/V5-DEST vector (Thermo Fisher Scientific).

Reagents and Antibodies

Recombinant SHH was expressed in bacteria and purified as previously described (Bishop et al., 2009). Recombinant WNT3A and BMP4 were purchased from R&D Systems and Thermo Fisher Scientific, respectively. Leupeptin, puromycin, blasticidin, and polybrene were purchased from Sigma-Aldrich. Vismodegib and Bafilomycin A1 were purchased from LC Labs. The following primary antibodies were used: mouse anti-GLI1 (2643; Cell Signaling; 1:1000), goat anti-GLI3 (AF3690; R&D; 1:200), rabbit anti-p38 (ab7952; Abcam; 1:2000), mouse anti-1D4 (The University of British Columbia; 1:5000), mouse α-Tubulin (T6199; Sigma-Aldrich; 1:10000), mouse acetylated-Tubulin (T6793; Sigma-Aldrich; 1:10000), mouse anti-NKX6.1 (F55A10, Developmental Studies Hybridoma Bank; 1:100), mouse anti-NKX2.2 (74.5A5, Developmental Studies Hybridoma Bank; 1:100), rabbit anti-PAX6 (AB2237; EMD Millipore; 1:1000), and Alexa Fluor 488 conjugated anti-Giantin (A488-114L; Covance; 1:500). Polyclonal antibodies against PTCH1, SMO (against both intracellular and extracellular epitopes (Milenkovic et al., 2009; Rohatgi et al., 2007)), SUFU (Humke et al., 2010), IQCE (Pusapati et al., 2014), GLI2 (Niewiadomski et al., 2014), ARL13B (Dorn et al., 2012), and OLIG2 (Novitch et al., 2003) have been described previously. Secondary antibodies conjugated to horseradish peroxidase or Alexa Fluor dyes were obtained from Jackson Laboratories and Thermo Fisher Scientific.

Ligand and Small Molecule Inhibitor Treatment Protocols

Throughout the text, the terms NoSHH, LoSHH, and HiSHH refer, respectively, to treatment with no SHH, a low, sub-saturating concentration of SHH, or a high, near-saturating concentration of SHH (see Figure S1B for the position of the LoSHH and HiSHH concentrations on the dose-response curve). For the directed experiments in NIH/3T3 cells, LoSHH and HiSHH concentrations used were 1 nM and 25 nM. For the genome-wide screens, LoSHH and HiSHH concentrations used were 3.2 nM and 25 nM. For NPCs, the LoSHH and HiSHH concentrations used were 5 nM and 25 nM. NIH/3T3 cells were treated with SHH for either 24 hours (CRISPR screens, qRT-PCR, immunoblotting, cycloheximide chase assay, SMO degradation, and internalization assays) or 6 hours (immunofluorescence studies). For NPCs, the duration of SHH exposure was 72 hours for all assays. Vismodegib was used at 1 μM. WNT3A was used at 200 ng/ml for 9 h and BMP4 was used at 50 ng/ml for 5 h.

Pooled Genome-wide CRISPR/Cas9 Screens

Our screen design was influenced by the lessons learned during our work on dissecting the WNT pathway using genetic screens in human haploid cells (Lebensohn et al., 2016). Since haploid cells lack primary cilia and so cannot transduce Hh signals, we used NIH/3T3 cells, mouse embryonic fibroblasts that are characterized by a robust transcriptional response to SHH. To isolate mutant cells with the desired Hh signaling phenotype, we used a fluorescence-based, quantitative transcriptional reporter of Hh signaling, which allowed the use of FACS to enrich cells with enhanced or reduced signaling phenotypes. The concentration-dependent properties of Hh ligands during development motivated us to perform screens at different concentrations of SHH. The Brie CRISPR library (Addgene #73633 (Doench et al., 2016)) was used to generate our genome-wide collection of mutant NIH/3T3 cells because this moderately sized library, which targets each of 19,674 mouse genes with ∼4 short guide RNAs (sgRNAs) and includes 1000 non-targeting

controls, provided the optimal balance between genome-wide coverage and the tractability of conducting multiple screens under varying conditions using a labor-intensive FACS-based enrichment scheme.

Brie library amplification, lentiviral production, functional titer determination, and transduction were performed as described previously with minor modifications (Joung et al., 2017). Briefly, the Brie library was amplified in Endura electrocompetent cells (Lucigen) and subjected to Next-Generation Sequencing (NGS) to determine sgRNA distribution. For lentivirus production, 18 million 293FT cells were seeded in T225 flasks (40 flasks in total) and transfected the following day with 3.4 μg pMD2.G (Addgene #12259), 6.8 μg psPAX2 (Addgene#12260), and 13.6 μg lentiviral target (CRISPR) plasmid, and 195 μl of 1 mg/ml polyethylenimine (Polysciences). 48 h post transfection, lentivirus was harvested, filtered through a 0.45 μ filter, aliquoted into multiple 50 ml tubes and stored at -80°C. The functional titer of the lentivirus was determined by crystal violent staining. GLI-Reporter cell line stably expressing Cas9 (NIH/3T3-CG) was transduced with the Brie library at a Multiplicity of Infection (MOI) =0.3 (364 million cells were transduced with 109 million Transduction Units (TUs) to achieve ~1000 fold representation of each sgRNA) in the presence of 10 μg/ml polybrene. 48 h post infection, cells were split and selected with puromycin (2 μg/ml) for seven days and frozen in aliquots of 5 million cells/vial. Genomic DNA (gDNA) was extracted from cells using Quick-gDNA Midiprep kit (Zymo Research) and subjected to NGS to determine sgRNA distribution. In all screens, 15 million cells were initially thawed into 3x15 cm plates and two days later split into 5x15 cm plates. Confluent cells were serum starved and left untreated (NoSHH) or treated for 24 h with SHH (LoSHH=3.2 nM or HiSHH=25 nM). Cells were trypsinized and 4 million cells were pelleted and frozen (unsorted population) and the remaining ~30 million cells (corresponding to 300-fold representation of each sgRNA in the Brie library) were sorted for cells with the lowest 10% of GFP fluorescence (HiSHH_Bot10% screen) or the highest 5% of GFP fluorescence (HiSHH_Top5%, LoSHH_Top5%, and NoSHH_Top5% screens). Each screen was performed twice under identical conditions.

Genomic DNA was extracted from unsorted and sorted cells and the sgRNA library was amplified by a two-step PCR protocol for NGS. In the first step, multiple PCR reactions (100 μl each) were set up to make sure that the entire gDNA was amplified using the following primers (NGS-1st PCR Fwd: 5'-aatggactatcatatgcttaccgtaacttgaaagtatttcg-3' and NGS-1st PCR Rev: 5'-ctcggtgccactttttcaagttgataacgg-3'). PCR product from multiple PCR reactions was pooled and 5 μl was used as a template for the second step PCR (100 μl reaction) using NGS library barcoded primers. The final PCR product was purified, quantified by qRT-PCR and subjected to sequencing on Illumina MiSeq with 45 cycles of read 1 using a custom primer (5'-gctcttccgatcttcttgtggaaaggacgaaacaccg-3') and 8 cycles of read 2 using Illumina index primer. In all the screens, we averaged ~100 reads per sgRNA in the library. For analysis, reads from the fastq files generated by sequencing were tallied for each guide by taking the first 20 base-pairs from each read and mapping that sequence to the identical short guide RNA sequence. For each screen, a table of reads per guide that includes the counts from the sorted and unsorted populations from both replicates was generated. The tables generated from the two independent duplicates of each screen were analyzed together by the MAGeCK computational tool (Li et al., 2014), specifying the 1000 control sgRNAs for normalization and generation of the null distribution for MAGeCK with the "–control-sgrna" option and computing the log fold change for the gene using the mean of all of the guides for a given gene with the "–gene-lfc-method mean" option.

Knockout of Candidate Genes in Pooled Cell Lines

The top two sgRNAs targeting candidate genes (based on MAGeCK analysis) were individually cloned into lentiCRISPR v2 plasmid (Addgene #52961; (Sanjana et al., 2014)). Lentivirus was produced as described above and used to infect GLI-Reporter NIH/3T3 cells, followed by selection with puromycin for 7 days. Pooled cell lines were analyzed by FACS for GFP fluorescence after treatment with LoSHH, HiSHH or NoSHH (see Figure S2 and associated text).

Clonal Cell Lines Carrying Deletions in Candidate Genes

Clonal knockout NIH/3T3 lines carrying deletions in candidate genes were generated using a dual sgRNA strategy. Briefly, two sgRNAs targeting candidate genes with an interval spanning anywhere from 50-600 bases (see Table S3) were designed using the CRISPR Design Tool (http://crispr.mit.edu) and cloned into pSpCas9(BB)-2A-GFP (PX458; Addgene #48138; (Ran et al., 2013)) and pSpCas9(BB)-2A-mCherry, the latter generated by replacing the GFP cassette in PX458 with mCherry. Five days after co-transfection into NIH/3T3 cells using X-tremeGENE 9 (Roche), GFP and mCherry double positive single cells were sorted into a 96-well plate using FACSAria II. Clonal lines were screened by PCR to detect excision of the genomic DNA between the two sgRNA cut sites (gels showing successful editing of candidate genes are shown in Table S3). When possible, knockout clones were further confirmed by Western Blotting using commercially available antibodies (*Rab34, Pdcl, and Mgrn1*). Clonal knockout mESCs were generated using the same two sgRNAs for each gene used in NIH/3T3 cells (see Table S3). For mESCs, sgRNAs were cloned into plasmid PX459 (Addgene #48139; (Ran et al., 2013)). Plasmids were electroporated into mESCs using the Lonza nucleofection system (Nucleofector 2b Device using the program A-023 and Lonza Cell Nucleofector Kit #VAPH-1001). mESCs were cultured under feeder free conditions in 2i media (Dulbecco's Modified Eagle's Medium F12 (Gibco) and Neurobasal Medium (Gibco) (1:1 ratio) supplemented with N-2 Supplement (Gibco), B-27 Supplement (Gibco), 1% penicillin/streptomycin (Gemini Bio-Products), 2mM L-glutamine (Gemini Bio-Products), 40 μg/ml Bovine Serum Albumin (Sigma), 55 μM 2-mercaptoethanol (Gibco), 5 μM CHIR99021 (Axon), 1 μM PD 98059 (Axon), and 1000 U/ml ESGRO LIF (Millipore)). 24 h post nucleofection, cells were selected with 1.5 μg/ml puromycin for 48 h. One week later, individual mESC colonies were manually picked, expanded, and screened by PCR to detect deletion of the genomic DNA segment between the two guides (see Table S3).

Real-Time Quantitative Reverse Transcription PCR (Real-Time qRT-PCR)

Real-Time qRT-PCR was performed using the *Power* SYBR Green Cells-to-CT Kit (Thermo Fisher Scientific) on a QuantStudio 5 Real-Time PCR System (Thermo Fisher Scientific) with custom designed primers for *Gli1* (Fwd: 5'-ccaagccaactttatgtcaggg-3' and Rev: 5'-agcccgcttctttgttaatttga-3'), *Axin2* (Fwd: 5'-aaacggattcaggtccttca-3' and Rev: 5'-caaagacatagccggaacct-3'), *Id1* (Fwd: 5'-aacggcgagatcagtgcctt-3' and Rev:5'- cctcagcgacacaagatgcgat-3'), and *Gapdh* (Fwd: 5'-agtggcaaagtggagatt-3' and Rev: 5'-gtggagtcatactggaaca-3'). Transcript levels relative to *gapdh* were calculated using the ΔCt method.

Western Blotting

Whole cell extracts from NIH/3T3 cells and NPCs were prepared in RIPA lysis buffer (50 mM Tris-HCl pH-7.4, 150 mM NaCl, 2% NP-40, 0.25% Deoxycholate, 0.1% SDS, 1mM DTT, 10% glycerol, 1x SIGMA*FAST* protease inhibitor cocktail (Sigma-Aldrich), and 1x PhosSTOP (Roche)). Samples were resuspended in NuPAGE-LDS sample buffer (Thermo Fisher Scientific), incubated at 37°C for 30 min, and subjected to SDS-PAGE. The resolved proteins were transferred onto a nitrocellulose membrane (Bio-Rad) using a wet electroblotting system (Bio-Rad) followed by immunoblotting.

SMO Trafficking Assays

Biotinylation of cell surface SMO with a non-cell permeable, thiol-cleavable probe was performed as described previously (Milenkovic et al., 2009). Briefly, cell culture plates were removed from the 37°C incubator and placed on an ice-chilled metal rack in a 4°C cold room. Growth medium was removed and cells were quickly washed thrice with ice-cold DPBS+ buffer (Dulbecco's PBS supplemented with 0.9 mM $CaCl_2$, 0.49 mM $MgCl_2.6H_2O$, 5.6 mM dextrose, and 0.3 mM sodium pyruvate). Cells were incubated with a freshly prepared solution of 0.4 mM Sulfo-NHS-SS-Biotin (Thermo Fisher Scientific) in DPBS+ buffer for 30 min. Unreacted Sulfo-NHS-SS-Biotin was quenched with Tris pH 7.4 at 50 mM for 10 min. Cells were then washed thrice with 1x Tris-buffered saline (25 mM Tris-HCl pH 7.4, 137 mM NaCl, and 2.7 mM KCl) and whole cell extracts were prepared in a buffer containing 50 mM Tris-HCl pH-7.4, 150 mM NaCl, 2% NP-40, 0.25% Deoxycholate, 1x Sigma-Fast protease inhibitor cocktail, and 1x Roche phosphatase inhibitor cocktail. Biotinylated proteins from clarified supernatants were captured on a streptavidin agarose resin (Solulink), washed, eluted in NuPAGE-LDS sample buffer containing 100 mM DTT at 37°C for 1 h to cleave and release biotinylated proteins, and assayed by immunoblotting. For degradation assays (shown in Figures 7C and 7D), cells were transferred to a 37°C incubator after cell-surface biotinylation and samples were harvested in a 4°C cold room after 0, 0.5, 1, 2, 3, 4, 5, and 6 h. For internalization assays (shown in Figures 7E and 7F), cells were pre-treated with 100 nM Bafilomycin A1 and 50 μM Leupeptin for 2 h to block lysosomal degradation. Cell-surface proteins were biotinylated as described above (with the exception that Bafilomycin A1 and Leupeptin were included at every step) and then transferred to a 37°C incubator for 0, 5, 10, and 20 minutes before being returned to 4°C. To estimate the amount of biotinylated SMO that was internalized during the 37°C incubation period, cells were treated twice with glutathione (50 mM glutathione, 75 mM NaCl, 75 mM NaOH, and 10% FBS in water) at 4°C for 15 min to cleave the biotin attached to any SMO left on the cell surface. Any biotinylated SMO that was internalized would be protected from glutathione cleavage. After quenching unreacted glutathione with iodoacetamide buffer (50 mM iodoacetamide and 1% BSA in D-PBS+ buffer) at 4°C for 20 min, cells were lysed (in the continued presence of 50 mM iodoacetamide) and the amount of biotinylated SMO remaining was assessed by streptavidin pull down followed by immunoblotting.

In Situ Hybridization

Atthog specific primers were designed using the program Primer3 (Fwd: 5'-acacgtgtgtgctgaaaagc-3' and Rev: 5'- gagattaaccctcac taaagggatgagcaggtaacccatctcc-3').' The underlined sequence marks the T3 polymerase binding site incorporated into the reverse primer. The *Atthog* probe was generated using a Digoxigenin (DIG) RNA Labeling Kit (Roche). Briefly, the probe was generated from the *in vitro* transcription of PCR products amplified from mouse neural progenitor cell cDNA. After overnight hybridization at 70°C, the signal was visualized using anti-DIG-alkaline phosphatase (AP) Fab fragments (Roche) and NBT/BCIP (Roche).

Analysis of Cilia Protein Localization by Immunofluorescence

Immunofluorescence staining of 1D4 tagged ATTHOG, endogenous SMO, PTCH1, GLI2, acTub, and ARL13B was performed as described previously (Pusapati et al., 2014). Staining of cell-surface SMO using an antibody against its extracellular domain (Milenkovic et al., 2009) was performed by excluding detergent from the blocking and antibody incubation steps. For cilia internalization assays (shown in Figures 7G and 7H), cell-surface SMO was labeled with the anti-SMO primary antibody at 4°C. Cells were then transferred to 37°C to allow trafficking for 0.25, 0.45, 1.5, 2, 3, and 4 h, followed by immediate fixation with 4% PFA and subsequent staining with a secondary antibody without cell permeabilization to only detect cell-surface SMO. After a second fixation step and washing, cells were permeabilized, stained with a cilia marker and imaged to quantify levels of SMO at primary cilia (see below for quantification details). Fluorescent images were collected on a Leica TCS SP5 confocal imaging system equipped with a 63x oil immersion objective with identical gain, offset, and laser power settings. Z-stacks covering 4 microns were captured and a max-projection of the z-stack was used for fluorescent intensity quantifications. Representative images were captured on the 63x oil objective with an 8x digital zoom option and processed with identical settings using Fiji (Schindelin et al., 2012) (https://fiji.sc).

For quantification of ciliary SMO and PTCH1 levels, Leica Image Files (LIF) were converted into matrices in MATLAB R2014a (MathWorks) using the *bfmatlab* toolbox. A max-z-projection was performed for each set of planes in a given field, followed by quantification of the cilia and protein-of-interest through the following steps. First, a two-dimensional median filter was applied to the cilia

channel (acTub or ARL13B) to remove fine noise, followed by a high-pass user-defined threshold for signal versus noise to generate a cilia mask in the cilia channel. Any group of contiguous pixels that had signal was labeled as a "potential cilium" and measured for area, eccentricity, solidity, intensity, and length. If the "potential cilium" did not meet the static thresholds for eccentricity or solidity, it was discarded from the dataset; if the "potential cilium" did not meet the user-defined thresholds for minimum area or minimum intensity, it was also discarded. Additionally, if the "potential cilium" was within a user-defined distance of another "potential cilium," then it was also discarded to avoid misquantification of false positives arising from non-specific staining. If a "potential cilium" met these tests, each pixel that composed the cilium was noted and mapped to the matrix that held the intensity values for the protein-of-interest channel. To generate the final intensity value for the protein-of-interest, the mean intensity of the pixels mapping to the protein-of-interest channel was calculated, and then a background correction was performed. This correction was performed by calculating the average intensity in a 100x100 pixel grid around each pixel in the protein-of-interest channel, followed by subtraction from the mean intensity. The final, protein-of-interest intensity value for each identified cilium was recorded along with the area and length of the cilium. For quantification of ciliary GLI2 levels, additional sets of parameters were included because GLI2 staining does not always perfectly overlap with the marker used to identify cilia (ARL13B). Therefore, we expanded the box encompassing an identified cilium by about 2.5 times to search for the GLI2 signal. The pixels contained in this box were then mapped to the GLI2 channel. A high-pass user-defined threshold for signal versus noise in the GLI2 channel was applied to generate a GLI2 mask, and any group of contiguous pixels that had signal was labeled as "potential GLI2" and measured for area and intensity. The "potential GLI2" object that had the maximum intensity and the largest area was taken as the true GLI2 signal, and the mean intensity of these pixels was calculated. A background-correction for the final GLI2 intensity value was performed in an identical manner as described earlier for SMO and PTCH1 quantifications. The scripts used for cilia imaging are publicly available at Github (https://github.com/heybhaven/Cilia_protein_quantification).

Protein localization data at cilia were displayed using violin plots, generated from background-corrected, fluorescence values from ~100 cilia per condition, unless otherwise noted. In a violin plot, the width of the shaded area represents the kernel probability density or the proportion of data located in a fluorescence interval centered at that point. Inside each violin, the median fluorescence and interquartile ranges are depicted as a circle and vertical line, respectively. Violin plots were generated in R (version 3.3.2) with the "ggplot2" package.

Protein Sequence Analysis, Domain Identification, and Phylogenetic Analysis

Iterative sequence profile searches were performed using the PSI-BLAST program run against the NCBI non-redundant (NR) protein database (Altschul et al., 1997). Multiple sequence alignments were built using the Kalign2 (Lassmann et al., 2009) and Bayesian Markov chain Monte Carlo (MCMC) based alignment methodology as implemented in the program GISMO (Neuwald and Altschul, 2016). Alignments were later manually adjusted based on profile-profile, secondary structure information, and structural alignments. Similarity-based clustering for both classification and discarding of nearly identical sequences was performed using the BLASTCLUST program (ftp://ftp.ncbi.nih.gov/blast/documents/blastclust.html). Secondary structures were predicted using the JPred (Cuff et al., 1998) and HHpred programs. For previously characterized domains, the PFAM database was used as a guide (Punta et al., 2012). Clustering with BLASTCLUST followed by multiple sequence alignment and further sequence profile searches were used to identify domains that were not detected by the original Pfam models. Structural visualization and manipulations were performed using the PyMol program (http://www.pymol.org). To assess the phylogenetic relationships, an approximate maximum likelihood method as implemented in the FasTree program was used (Price et al., 2009). To upsurge the accuracy of topology, we augmented the number of rounds of minimum-evolution subtree-prune-regraft (SPR) moves to 4 (-spr 4) as well as utilized the options -mlacc and -slownni to make the maximum-likelihood nearest-neighbor interchanges (NNIs) more exhaustive. The FigTree program was used to render phylogenetic trees (http://tree.bio.ed.ac.uk/software/figtree/). The in-house TASS package, which comprises a collection of Perl scripts, was used to automate aspects of large-scale analysis of sequences. Organism abbreviations used in the figure panels (alphabetical order): Acar, *Anolis carolinensis*; Adig, *Acropora digitifera*; Aque, *Amphimedon queenslandica*; Bbel, *Branchiostoma belcheri*; Bflo, *Branchiostoma floridae*; Bgla, *Biomphalaria glabrate*; Cele, *Caenorhabditis elegans*; Cgig, *Crassostrea gigas*; Cint, *Ciona intestinalis*; Ctel, *Capitella teleta*; Dmel, *Drosophila melanogaster*; Dpul, *Daphnia pulex*; Drer, *Danio rerio*; Epal, *Exaiptasia pallida*; Ggal, *Gallus gallus*; Hduj, *Hypsibius dujardini*; Hsap, *Homo sapiens*; Isca, *Ixodes scapularis*; Lana, *Lingula anatine*; Lcha, *Latimeria chalumnae*; Mbre, *Monosiga brevicollis*; Mmus, *Mus musculus*; Nvec, *Nematostella vectensis*; Obim, *Octopus bimaculoides*; Pcau, *Priapulus caudatus*; Skow, *Saccoglossus kowalevskii*; Spur, *Strongylocentrotus purpuratus*; Xlae, *Xenopus laevis*.

Gene Ontology (GO) Analysis

A list composed of 641 genes, which includes genes identified as hits in all four screens with an FDR-corrected p-value ≤ 0.1, was used as the input to query the DAVID Functional Annotation Tool (Huang et al., 2008) to find enriched GO Terms or the Jensen DISEASES library (Pletscher-Frankild et al., 2015) of gene-disease associations using the *Enrichr* server (Kuleshov et al., 2016). The p-values reported are the ones corrected for multiple hypothesis testing.

QUANTIFICATION AND STATISTICAL ANALYSIS

All four screens (Figures 1B–1E) were performed twice under independent conditions and the duplicates from each screen were analyzed together using the MAGeCK tool. For Phase I of the validation (Figure S2A), two cell lines expressing different sgRNAs against each candidate gene were generated. Cell lines expressing short guides against putative positive regulators (Figure S2B) were analyzed three independent times and cell lines expressing short guides against putative negative regulators (Figures S2C–S2E) were analyzed two independent times. Within each of these independent experiments, each measurement represents median fluorescence from ~10,000 cells analyzed by FACS.

For Phase II of the validation (Figure 3), 2-3 independent clonal cell lines (represented by circles, triangles or squares in the Figure 3) were isolated for each genotype and the mean *Gli1* mRNA levels (Figures 3A and 3B) or median reporter fluorescence (Figures 3C and 3D) across these independent clonal cell lines was averaged and depicted as bars. For each cell line, the *Gli1* mRNA levels were taken as the average from two technical duplicates and the average median reporter fluorescence (~10,000 cells) from two independent experiments. Thus, Phase II of validation was scored based on reproducibility across multiple, independent clonal cell lines, not just across biological or technical replicates in the same cell line.

All the cell biological and biochemical experiments shown in Figures 4, 5, 6, 7, and 8 to characterize each of the six genes that emerged from Phase II of the validation were performed at least twice in each of two independent clonal cell lines, with similar results. Space permitting, data from two clonal cell lines is shown together. In some cases, analysis of only one clonal cell line is shown in the main figure, with data from the second clonal cell line presented in a supplemental figure panel noted in the figure legend. A phenotype is discussed in the text only if it was observed in both clonal lines.

The statistical significance between two groups was determined by an unpaired Student's *t*-test (Hh FACS reporter assays and qRT-PCR data). The statistical significance of fluorescent intensity comparisons between two or multiple groups was determined by Mann-Whitney and Kruskal-Wallis non-parametric ANOVA tests, respectively.

DATA AND SOFTWARE AVAILABILITY

All Fastq files from NGS have been deposited into the NIH Short Read Archive (SRA) with Study accession number SRP116669. The scripts used for cilia imaging are publicly available at Github (https://github.com/heybhaven/Cilia_protein_quantification).

Developmental Cell, Volume *44*

Supplemental Information

CRISPR Screens Uncover Genes that Regulate Target Cell Sensitivity to the Morphogen Sonic Hedgehog

Ganesh V. Pusapati, Jennifer H. Kong, Bhaven B. Patel, Arunkumar Krishnan, Andreas Sagner, Maia Kinnebrew, James Briscoe, L. Aravind, and Rajat Rohatgi

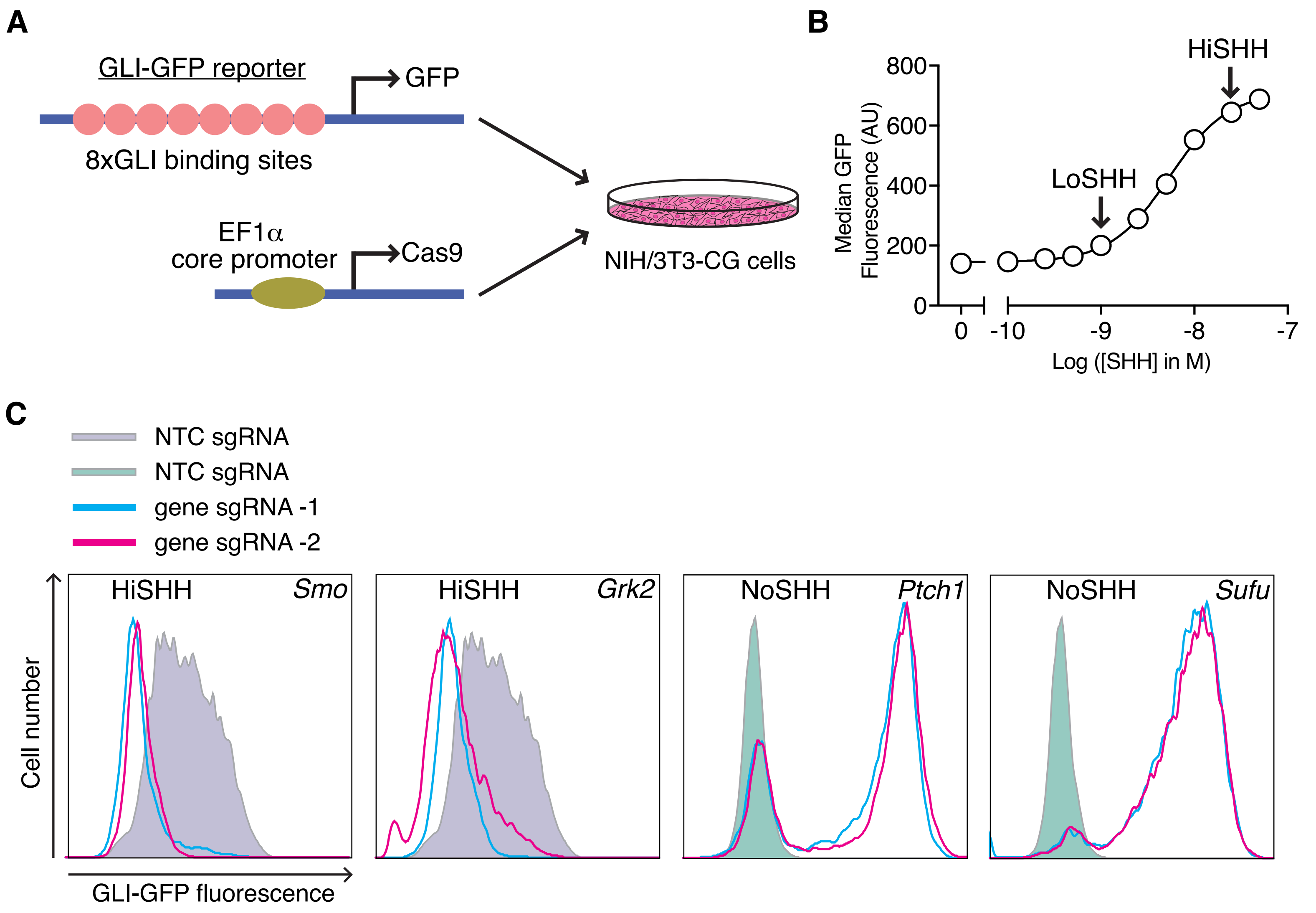
A
GLI-GFP reporter
GFP
8xGLI binding sites
EF1α
core promoter
Cas9
NIH/3T3-CG cells
B
HiSHH
LoSHH
Median GFP
Fluorescence (AU)
800
600
400
200
0
0
-10
-9
-8
-7
Log ([SHH] in M)
C
NTC sgRNA
NTC sgRNA
gene sgRNA -1
gene sgRNA -2
HiSHH
Smo
HiSHH
Grk2
NoSHH
Ptch1
NoSHH
Sufu
Cell number
GLI-GFP fluorescence

Figure S1. Related to Figure 1: **Characterization of the NIH/3T3-CG reporter line used in the screens.** (A) NIH/3T3-CG is a clonal derivative of NIH/3T3 cells transduced with a GFP gene driven by a synthetic Hh-responsive promoter composed of eight consecutive binding sites for the GLI transcription factors (GLI-GFP reporter) and a Cas9 gene driven by an EF1α core promoter. (B) SHH induces a dose-dependent increase in the median GFP fluorescence from ~10,000 NIH/3T3-CG cells. Arrows indicate the points on the dose-response curve that correspond to the LoSHH and HiSHH concentrations used throughout the text. (C) Flow cytometry histograms showing the distribution of GFP fluorescence in ~10,000 NIH/3T3-CG cells after transduction with two independent sgRNAs against known positive (*Smo*, *Grk2*) and negative (*Sufu*, *Ptch1*) regulators or one non-targeting control (NTC) sgRNA, followed by treatment with the indicated doses of SHH.

Figure S2

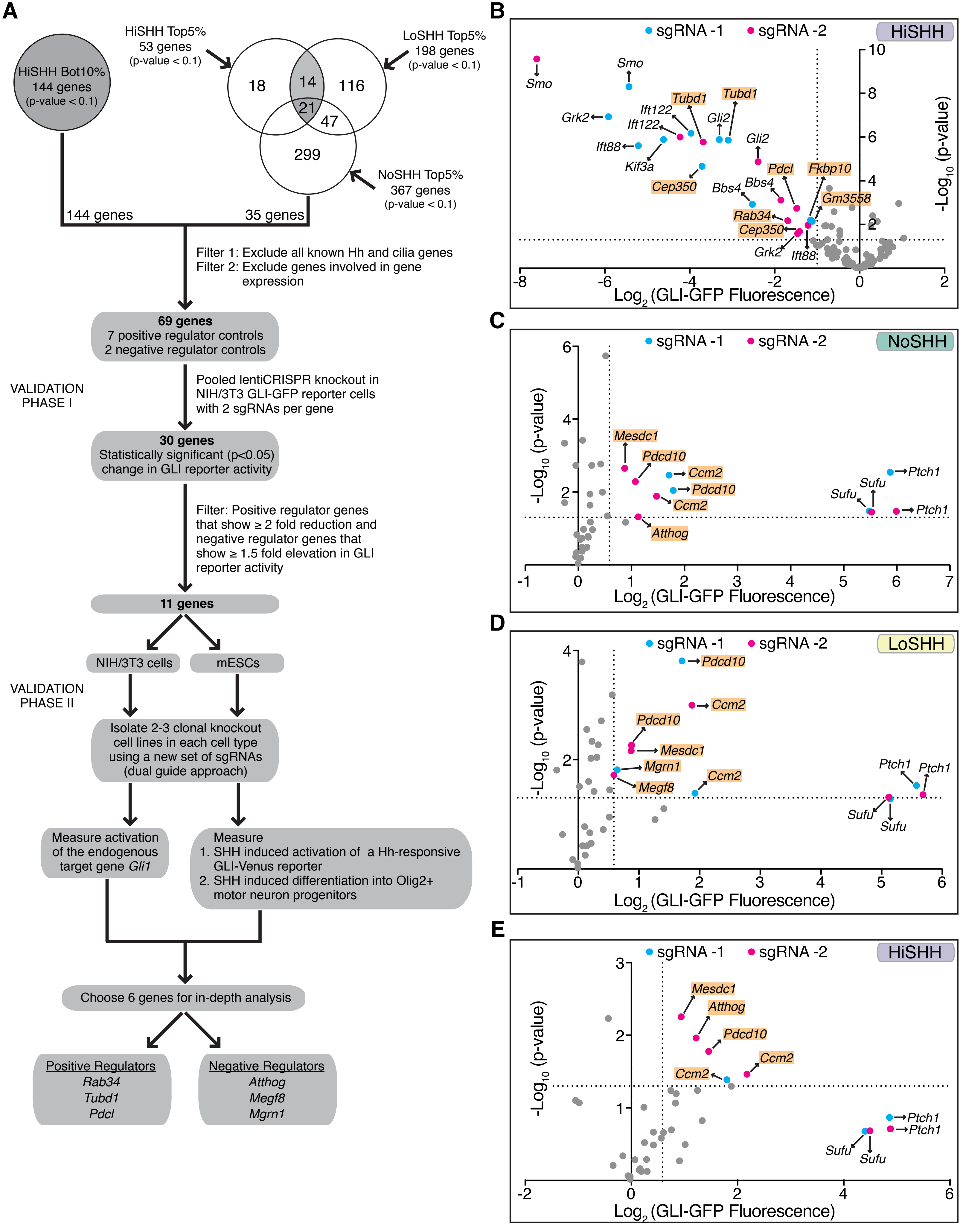
A
HiSHH Bot10%
144 genes
(p-value < 0.1)
HiSHH Top5%
53 genes
(p-value < 0.1)
LoSHH Top5%
198 genes
(p-value < 0.1)
NoSHH Top5%
367 genes
(p-value < 0.1)
18
14
116
21
47
299
144 genes
35 genes
Filter 1: Exclude all known Hh and cilia genes
Filter 2: Exclude genes involved in gene expression
69 genes
7 positive regulator controls
2 negative regulator controls
VALIDATION PHASE I
Pooled lentiCRISPR knockout in NIH/3T3 GLI-GFP reporter cells with 2 sgRNAs per gene
30 genes
Statistically significant (p<0.05) change in GLI reporter activity
Filter: Positive regulator genes that show ≥ 2 fold reduction and negative regulator genes that show ≥ 1.5 fold elevation in GLI reporter activity
11 genes
NIH/3T3 cells
mESCs
VALIDATION PHASE II
Isolate 2-3 clonal knockout cell lines in each cell type using a new set of sgRNAs (dual guide approach)
Measure activation of the endogenous target gene Gli1
Measure
1. SHH induced activation of a Hh-responsive GLI-Venus reporter
2. SHH induced differentiation into Olig2+ motor neuron progenitors
Choose 6 genes for in-depth analysis
Positive Regulators
Rab34
Tubd1
Pdcl
Negative Regulators
Atthog
Megf8
Mgrn1
B
sgRNA -1
sgRNA -2
HiSHH
Smo
Grk2
Ift122
Ift88
Kif3a
Tubd1
Gli2
Cep350
Pdcl
Fkbp10
Bbs4
Gm3558
Rab34
Ift88
-Log10 (p-value)
Log2 (GLI-GFP Fluorescence)
C
NoSHH
Mesdc1
Pdcd10
Ccm2
Atthog
Sufu
Ptch1
D
LoSHH
Pdcd10
Ccm2
Mesdc1
Mgrn1
Megf8
Ptch1
Sufu
E
HiSHH
Mesdc1
Atthog
Pdcd10
Ccm2
Ptch1
Sufu

Figure S2. Related to Figure 3: Flow chart for the validation strategy for top hits from all four genome-wide screens and validation of candidate genes by gene-disruption using individually cloned sgRNAs. (A) Flowchart showing our validation pipeline. Genes from the HiSHH_Bot10% screen (*n*=144) enriched with an FDR-corrected p-value<0.1 (shaded circle at top left) and genes (*n*=35) that were enriched in both the HiSHH_Top5% and the LoSHH_Top5% screens with an FDR-corrected p-value<0.1 (shaded section of the Venn diagram at the top right) were passed through two filters, yielding 69 genes that entered Phase I of validation. In Phase I, the top two sgRNAs from the Brie library targeting each of these 69 genes (a total of 138 sgRNAs) were individually expressed in NIH/3T3-CG cells using a lentiviral vector and tested for effects on the GLI-GFP reporter after exposure to NoSHH, HiSHH, or LoSHH (for negative regulators only; B-E). For Phase II of validation, eleven genes (10 in mESCs) were selected for clonal knockout analysis in NIH/3T3 cells and NPCs derived from mESCs (see Figure 3). Finally, 6 genes were chosen for in-depth analysis based on their strong effect on the SHH-induced Hh responses in both NIH/3T3s and NPCs. (B) Fold-activation of the GLI-GFP reporter in pooled NIH/3T3 GLI-GFP reporter cell lines transduced with two sgRNAs against each of the candidate positive regulators from the HiSHH_Bot10% screen after treatment with HiSHH. The median GFP fluorescence from ~10,000 cells, expressed relative that of a control cell line expressing a non-targeting control (NTC) sgRNA, from three independent experiments was used to calculate the overall mean change in GLI-

GFP fluorescence (x-axis) and a *p*-value (y-axis) using the unpaired student's *t*-test. Analogous experiments with candidate negative regulators were performed under conditions of NoSHH (C), LoSHH (D), or HiSHH (E). Dotted lines denote cut-offs for the fold-change in GLI-GFP fluorescence and *p*-value (<0.05) used to select genes (highlighted in orange on the plots) for Phase II of the validation pipeline. *Smo, Grk2, Ift88*, *Ift122, Kif3a, Gli2,* and *Bbs4* served as controls for positive regulators; *Ptch1* and *Sufu* served as controls for negative regulators.

Figure S3

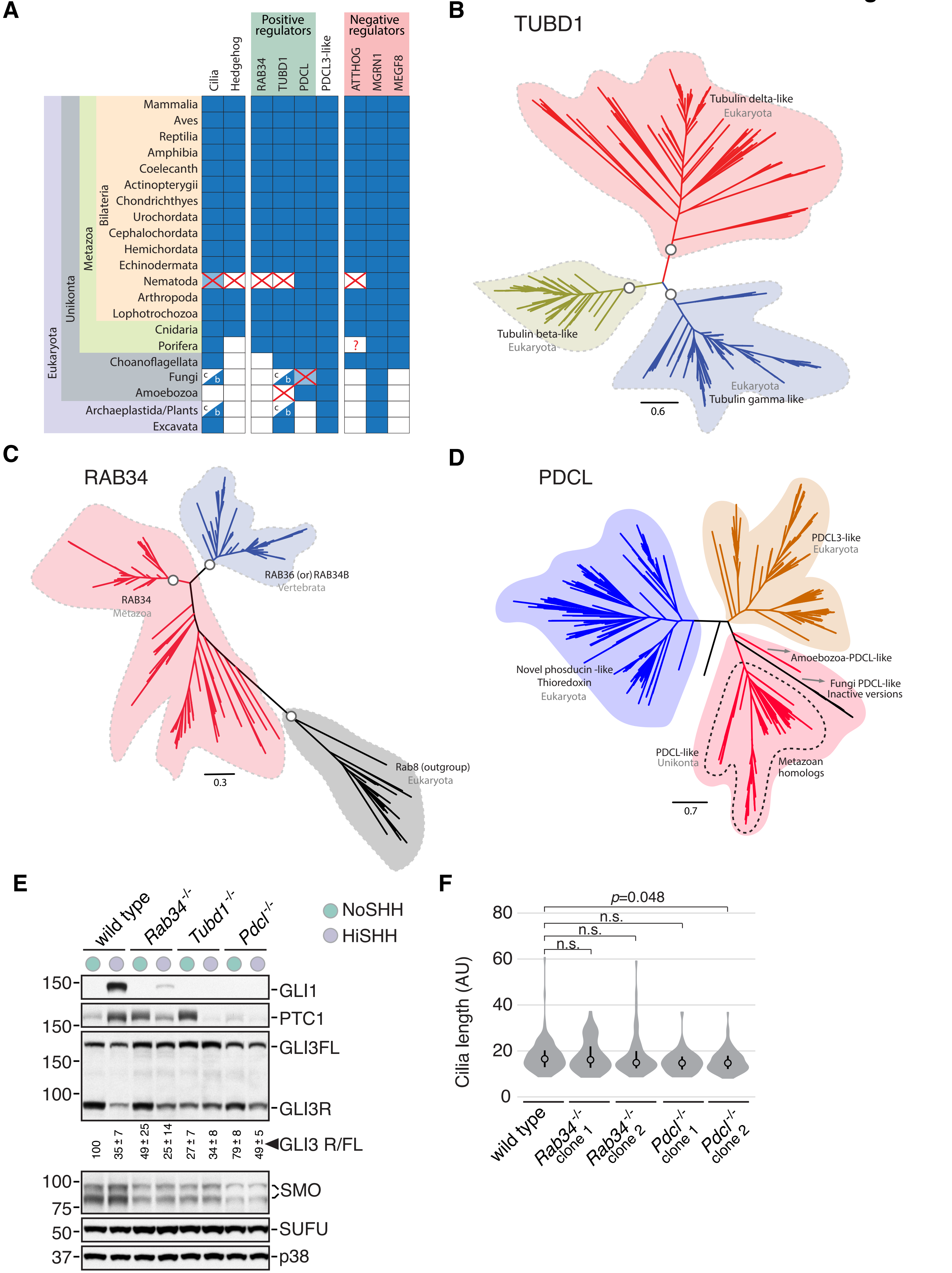
A
Positive regulators
Negative regulators
Cilia
Hedgehog
RAB34
TUBD1
PDCL
PDCL3-like
ATTHOG
MGRN1
MEGF8
Mammalia
Aves
Reptilia
Amphibia
Coelecanth
Actinopterygii
Chondrichthyes
Urochordata
Cephalochordata
Hemichordata
Echinodermata
Nematoda
Arthropoda
Lophotrochozoa
Cnidaria
Porifera
Choanoflagellata
Fungi
Amoebozoa
Archaeplastida/Plants
Excavata
Bilateria
Metazoa
Unikonta
Eukaryota
?
B
TUBD1
Tubulin delta-like
Eukaryota
Tubulin beta-like
Eukaryota
Eukaryota
Tubulin gamma like
0.6
C
RAB34
RAB34
Metazoa
RAB36 (or) RAB34B
Vertebrata
Rab8 (outgroup)
Eukaryota
0.3
D
PDCL
PDCL3-like
Eukaryota
Amoebozoa-PDCL-like
Fungi PDCL-like
Inactive versions
Novel phosducin -like
Thioredoxin
Eukaryota
PDCL-like
Unikonta
Metazoan
homologs
0.7
E
wild type
Rab34-/-
Tubd1-/-
Pdcl-/-
NoSHH
HiSHH
150
GLI1
PTC1
150
GLI3FL
150
100
GLI3R
100
35 ± 7
49 ± 25
25 ± 14
27 ± 7
34 ± 8
79 ± 8
49 ± 5
GLI3 R/FL
100
75
SMO
50
SUFU
37
p38
F
p=0.048
n.s.
n.s.
n.s.
80
60
40
20
0
Cilia length (AU)
wild type
Rab34-/- clone 1
Rab34-/- clone 2
Pdcl-/- clone 1
Pdcl-/- clone 2

Figure S3. Related to Figure 4: Phylogenetic relationships of the Hh signaling regulators identified by CRISPR-based screens. (A) Phyletic patterns of the positive and negative regulators of Hh signaling from our screens relative to the evolution of cilia and Hh signaling. Blue fill denotes presence, white fill denotes absence prior to origin of the gene/pathway/organelle, a red cross denotes a loss, and a red "?" denotes uncertainty given the insufficiency of whole genome data. Half-filled squares shown for fungi and plants denote the presence in basal lineages but a loss in crown lineages. TUBD1, which localizes to centrioles in metazoans, is lost in nematodes, which have an aberrant centriolar morphology and composition and have lost both motile cilia (faded blue box) and Hh signaling but have retained primary (sensory) cilia. The faded blue box for PDCL indicates loss across most fungi but sporadic retention of divergent versions (see D). (B, C, D) Unrooted tree topologies showing evolutionary relationships of TUBD1, RAB34, and PDCL to their closest related families as identified by iterative sequence profile searches. Divergent families forming monophyletic clades are highlighted in distinct colors within each tree. The evolutionary provenance of each family traced in this study is indicated below the protein name. Filled white circles with black outlines on the nodes denote a support of ≥ 0.9 using the Shimodaira-Hasegawa test on 1000 resamples. Phylogenetic analysis of the phosducin-like clade (A and D) indicates that a single member, prototyped by *Pdcl3,* was ancestrally present in eukaryotes and functioned independently of the presence of heterotrimeric G-protein function.

However*, Pdcl* itself is only found in the unikont clade of eukaryotes, including amoebozoans, fungi, and metazoans. Notably, *Pdcl* (unlike *Pdcl3*) is lost or potentially inactive in most fungi and is anomalously fast-evolving in nematodes, both of which lack motile cilia and canonical Hh signaling. (E) Immunoblots were used to measure levels of the indicated Hh pathway proteins in extracts of *Rab34*$^{-/-}$, *Tubd1*$^{-/-}$, or *Pdcl*$^{-/-}$ NIH/3T3 cells. This data is derived from a set of clonal cell lines distinct from that used in Figure 4B. (F) Violin plots showing ciliary length distributions of either wild type NIH/3T3 or two clonal NIH/3T3 cell lines carrying deletions in *Rab34* and *Pdcl*. Statistical significance was determined by the Kruskal-Wallis test and depicted as follows: $p > 0.05$ (ns, not significant).

Figure S4

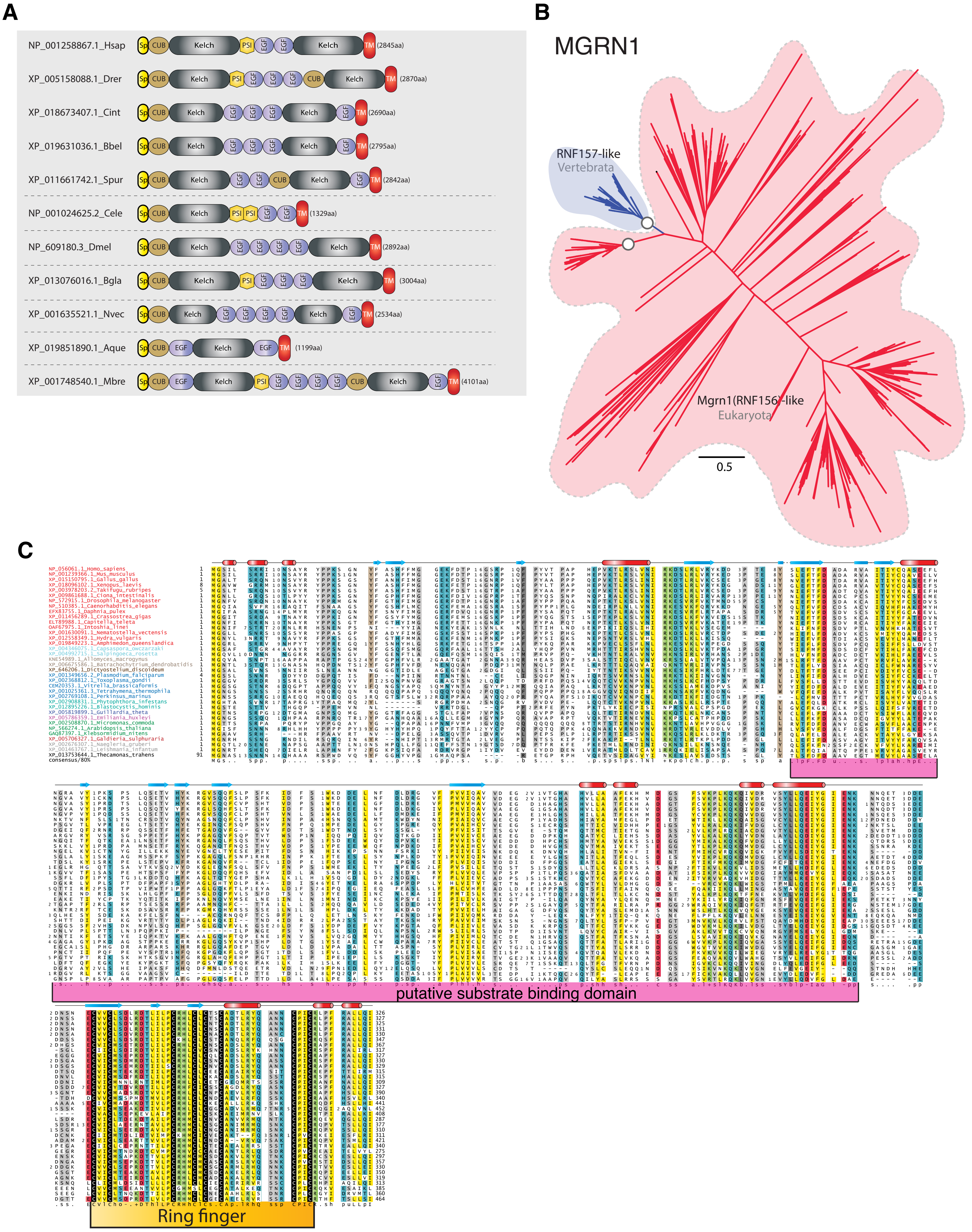
A
NP_001258867.1_Hsap
XP_005158088.1_Drer
XP_018673407.1_Cint
XP_019631036.1_Bbel
XP_011661742.1_Spur
NP_001024625.2_Cele
NP_609180.3_Dmel
XP_013076016.1_Bgla
XP_001635521.1_Nvec
XP_019851890.1_Aque
XP_001748540.1_Mbre
(2845aa)
(2870aa)
(2690aa)
(2795aa)
(2842aa)
(1329aa)
(2892aa)
(3004aa)
(2534aa)
(1199aa)
(4101aa)
Sp
CUB
Kelch
PSI
EGF
TM
B
MGRN1
RNF157-like
Vertebrata
Mgrn1(RNF156)-like
Eukaryota
0.5
C
putative substrate binding domain
Ring finger

Figure S4. Related to Figure 5: Sequence analysis of the two Hh attenuators MEGF8 and MGRN1. (A) Domain architectures of selected MEGF8 homologs across all major holozoan lineages. The proteins are represented by GenBank identifiers (GIs) followed by their species abbreviations (see Methods). Domain abbreviations: SP, signal peptide; TM, transmembrane region; CUB (complement C1r/C1s, Uegf Bmp1) domain; Kelch repeats; PSI repeats, cysteine rich repeats found in plexins, semaphorins and integrins; EGF, Epidermal growth factor. (B) Unrooted tree topologies showing evolutionary relationships of MGRN1 to its closest related families, depicted as in Figure S3. (C) Multiple sequence alignment of the E3 ubiquitin-protein ligase MGRN1. Predicted secondary structure is denoted above the alignment. MGRN1 contains a RING domain likely to interact with E2 ubiquitin conjugating enzymes and a previously unannotated but conserved N-terminal domain that is likely to bind substrates, perhaps microtubules. The proteins names are represented by GIs followed by their full species names (see Methods) and are colored per major lineages of holozoans.

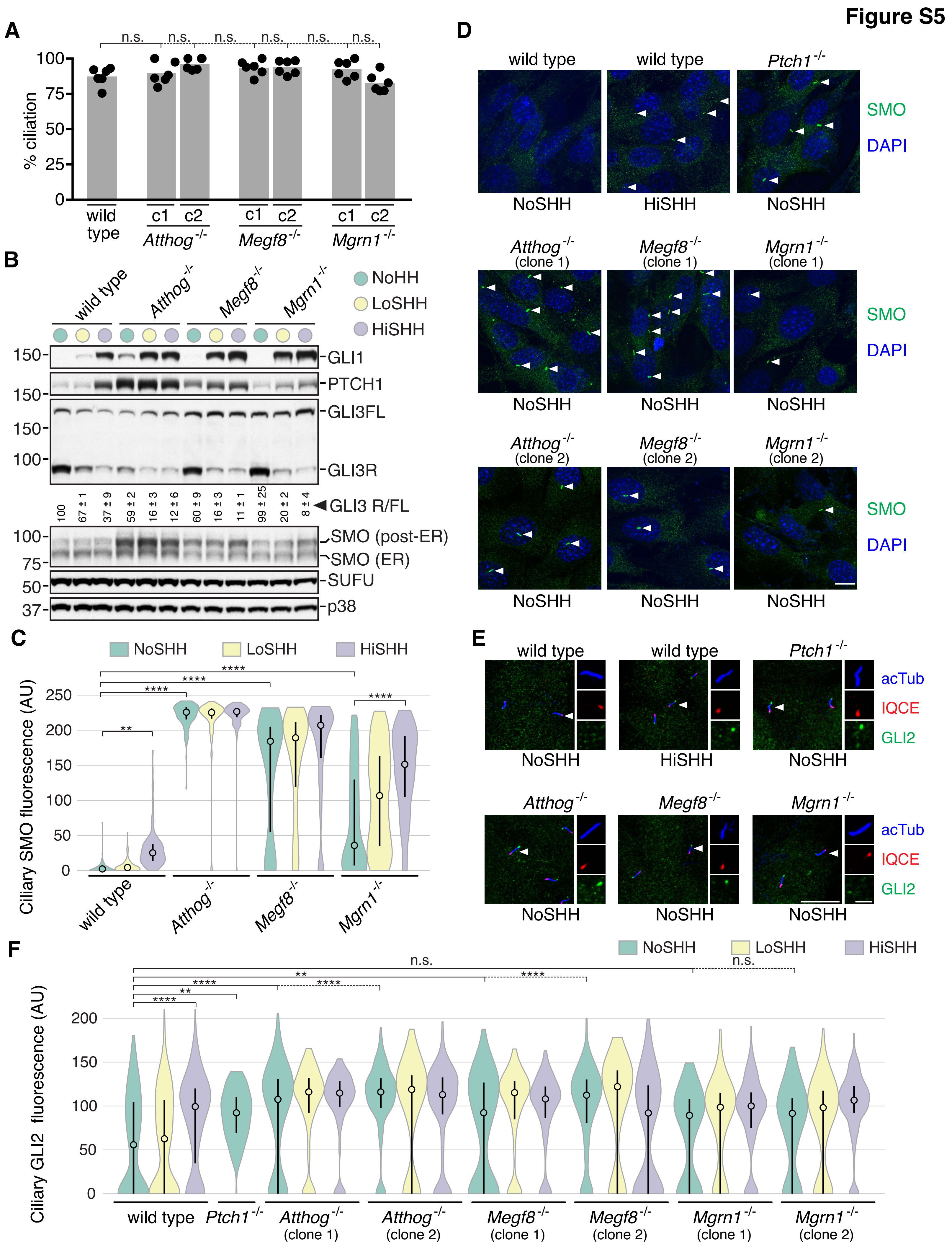
Figure S5
A
% ciliation
n.s.
wild type
c1 c2
Atthog-/-
Megf8-/-
Mgrn1-/-
B
wild type
Atthog-/-
Megf8-/-
Mgrn1-/-
NoHH
LoSHH
HiSHH
GLI1
PTCH1
GLI3FL
GLI3R
100
67 ± 1
37 ± 9
59 ± 2
16 ± 3
12 ± 6
60 ± 9
16 ± 3
11 ± 1
99 ± 25
20 ± 2
8 ± 4
GLI3 R/FL
SMO (post-ER)
SMO (ER)
SUFU
p38
C
NoSHH
LoSHH
HiSHH
Ciliary SMO fluorescence (AU)
D
wild type
Ptch1-/-
Atthog-/- (clone 1)
Megf8-/- (clone 1)
Mgrn1-/- (clone 1)
Atthog-/- (clone 2)
Megf8-/- (clone 2)
Mgrn1-/- (clone 2)
SMO
DAPI
NoSHH
HiSHH
E
acTub
IQCE
GLI2
F
Ciliary GLI2 fluorescence (AU)
wild type
Ptch1-/-
Atthog-/- (clone 1)
Atthog-/- (clone 2)
Megf8-/- (clone 1)
Megf8-/- (clone 2)
Mgrn1-/- (clone 1)
Mgrn1-/- (clone 2)

Figure S5. Related to Figure 5: The deletion of *Atthog, Megf8,* and *Mgrn1* increases ciliary levels of SMO and GLI2. (A) Frequency of ciliation in wild type NIH/3T3 cells and two independent clonal *Atthog*$^{-/-}$, *Megf8*$^{-/-}$, and *Mgrn1*$^{-/-}$ cell lines. Bars represent the mean ciliation frequency derived from six separate fields, each shown by a black dot (n~200 cells). (B) Immunoblots were used to measure levels of the indicated Hh pathway proteins in extracts of *Atthog*$^{-/-}$, *Megf8*$^{-/-}$, and *Mgrn1*$^{-/-}$ cells treated with LoSHH or HiSHH. Analogous data from an independent set of clonal cell lines is shown in Figure 5A. (C) Violin plots showing fluorescence intensity distributions of SMO in primary cilia (n=100 cilia) of the indicated NIH/3T3 cell lines. Analogous data from an independent set of clonal cell lines is shown in Figure 5D. (D) Cell-surface SMO was detected by immunofluorescence in intact (non-permeabilized) NIH/3T3 cell lines using an antibody against an epitope on the extracellular region of SMO. Arrowheads show surface SMO enriched in primary cilia. (E and F) Representative micrographs (E) and corresponding violin plots (F) showing the accumulation of endogenous GLI2 at the tips of primary cilia in clonal NIH/3T3 cell lines. AcTub staining (blue) extends along the entire length of the ciliary axoneme, IQCE staining (red) marks the base of cilia and GLI2 staining (green) is seen at the tip of cilia, at the opposite end of the axoneme from IQCE staining. Statistical significance was determined by the Kruskal-Wallis test ($p < 0.01$ (**), $p < 0.0001$ (****), and $p > 0.05$ (ns)). Scale bars, 10 μm in merged panels and 2 μm in zoomed displays.

Figure S6

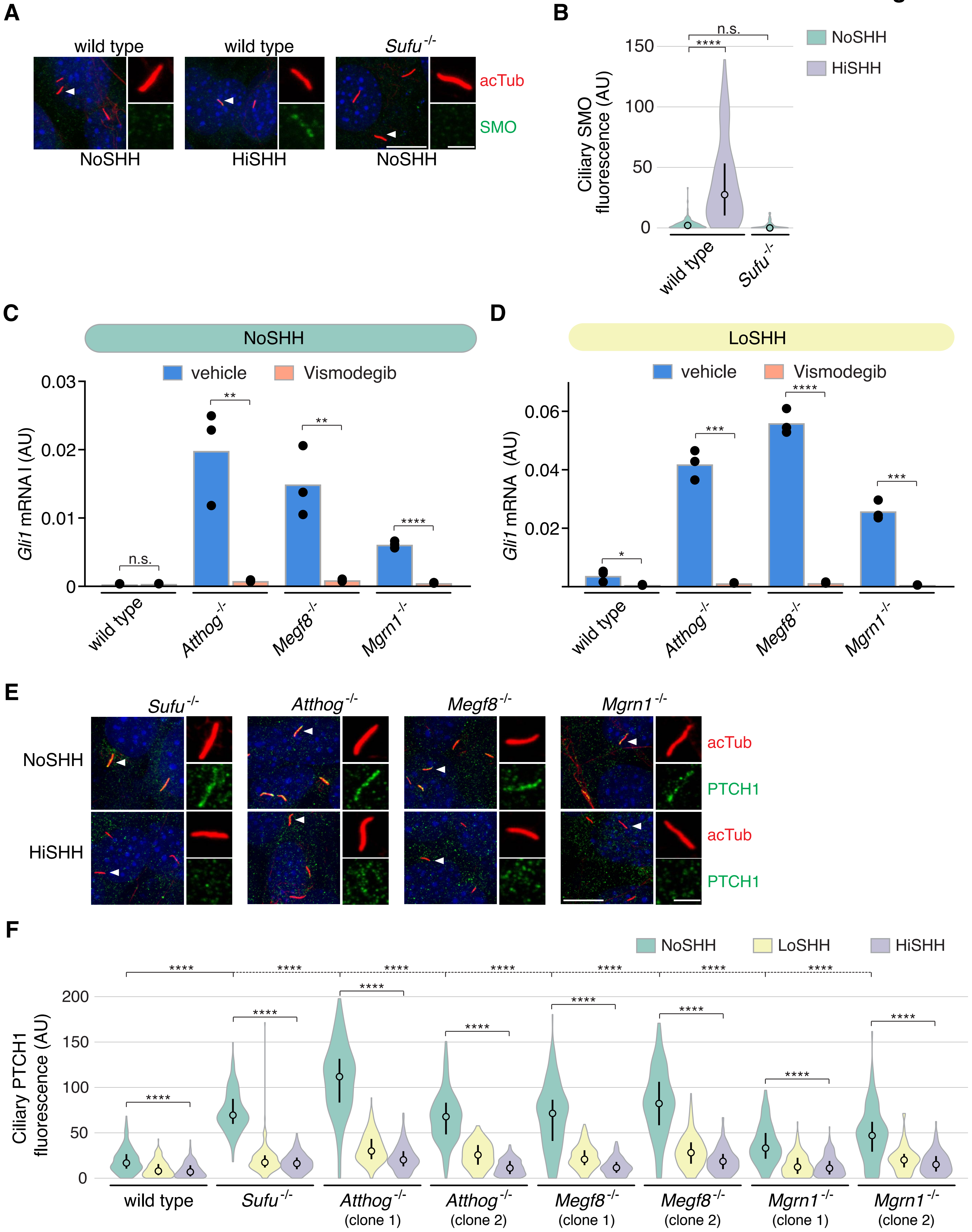

A
wild type
wild type
Sufu -/-
acTub
SMO
NoSHH
HiSHH
NoSHH
B
n.s.
****
NoSHH
HiSHH
150
100
50
0
Ciliary SMO fluorescence (AU)
wild type
Sufu -/-
C
NoSHH
vehicle
Vismodegib
0.03
0.02
0.01
0
Gli1 mRNAl (AU)
n.s.
**
**
****
wild type
Atthog -/-
Megf8 -/-
Mgrn1 -/-
D
LoSHH
vehicle
Vismodegib
0.06
0.04
0.02
Gli1 mRNA (AU)
*
***
****
***
wild type
Atthog -/-
Megf8 -/-
Mgrn1 -/-
E
Sufu -/-
Atthog -/-
Megf8 -/-
Mgrn1 -/-
NoSHH
HiSHH
acTub
PTCH1
acTub
PTCH1
F
NoSHH
LoSHH
HiSHH
200
150
100
50
0
Ciliary PTCH1 fluorescence (AU)
****
wild type
Sufu -/-
Atthog -/- (clone 1)
Atthog -/- (clone 2)
Megf8 -/- (clone 1)
Megf8 -/- (clone 2)
Mgrn1 -/- (clone 1)
Mgrn1 -/- (clone 2)

Figure S6. Related to Figure 5: ATTHOG, MEGF8, and MGRN1 influence the ciliary trafficking of SMO, but not of PTCH1. (A and B) Representative micrographs (A) and corresponding violin plots (B, n=100 cilia per condition) showing the abundance of endogenous SMO in primary cilia of wild type NIH/3T3 cells and *Sufu*$^{-/-}$ cells left untreated or treated with HiSHH. (C and D) Vismodegib blocks both basal (C) and LoSHH-induced (D) *Gli1* mRNA levels in *Atthog*$^{-/-}$, *Megf8*$^{-/-}$, and *Mgrn1*$^{-/-}$ cell lines. Analogous data from an independent set of clonal cell lines is shown in Figures 5E and 5F. Bars depict the mean *Gli1* mRNA level derived from three independent replicates, each denoted by a black dot. Statistical significance was determined by the unpaired student's *t*-test ($p < 0.05$ (*), $p < 0.01$ (**), $p < 0.001$ (***), $p < 0.0001$ (****), and $p > 0.05$ (ns)). (E and F) Representative micrographs (E) and corresponding violin plots (F, n=100 cilia per condition) showing levels of endogenous PTCH1 in primary cilia of NIH/3T3 cell lines left untreated or treated with the indicated doses of SHH. Statistical significance in (B) and (F) was determined by the Kruskal-Wallis test and depicted as $p < 0.0001$ (****) and $p > 0.05$ (ns). Scale bars, 10 μm in merged panels and 2 μm in zoomed displays.

Figure S7

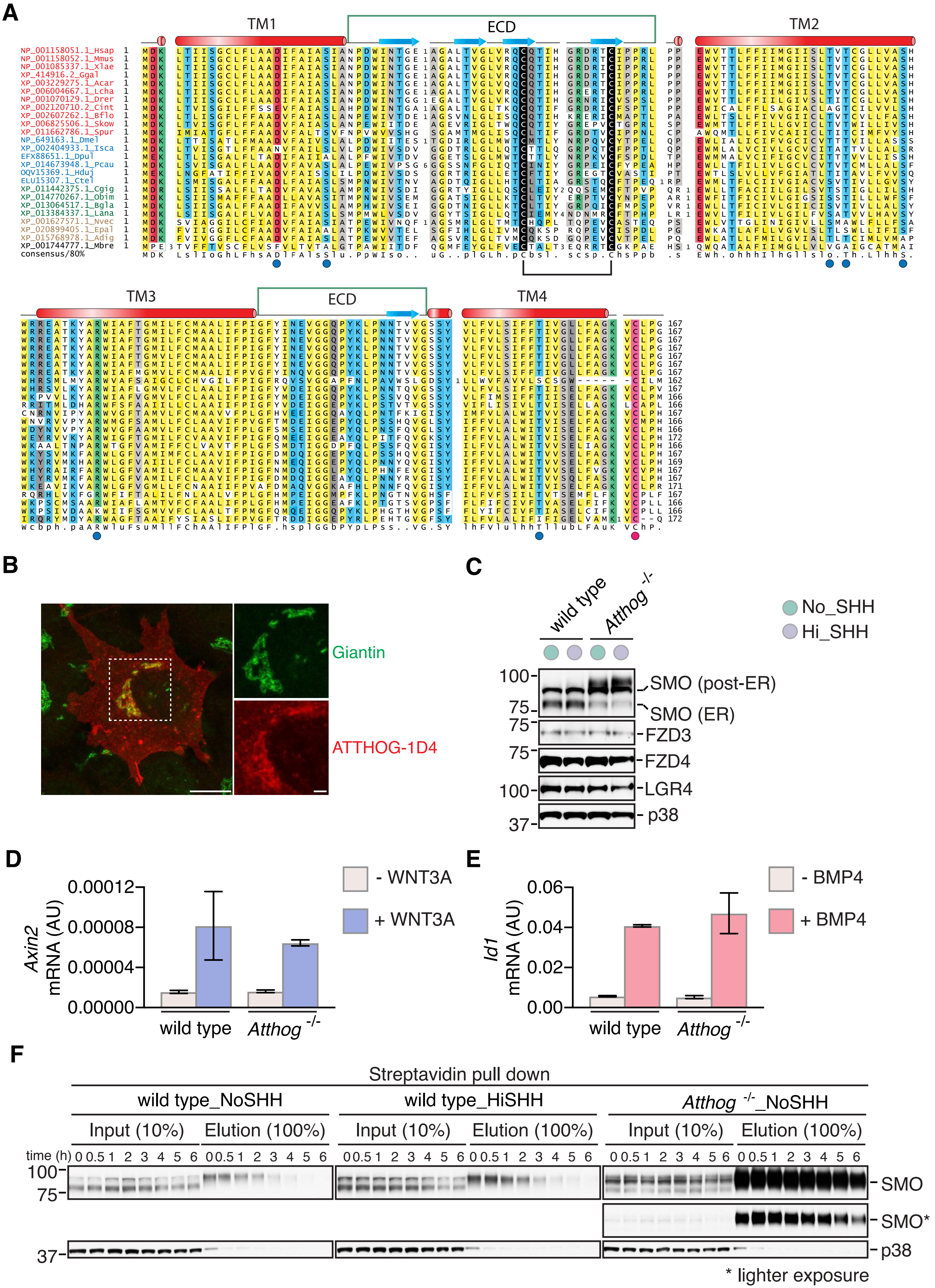
A
TM1
ECD
TM2
TM3
ECD
TM4
NP_001158051.1_Hsap
NP_001158052.1_Mmus
NP_001085337.1_Xlae
XP_414916.2_Ggal
XP_003229275.1_Acar
XP_006004667.1_Lcha
NP_001070129.1_Drer
XP_002120710.2_Cint
XP_002607262.1_Bflo
XP_006825506.1_Skow
XP_011662786.1_Spur
NP_649163.1_Dmel
XP_002404933.1_Isca
EFX88651.1_Dpul
XP_014673948.1_Pcau
OQV15369.1_Hduj
ELU15307.1_Ctel
XP_011442375.1_Cgig
XP_014770267.1_Obim
XP_013064517.1_Bgla
XP_013384337.1_Lana
XP_001627571.1_Nvec
XP_020899405.1_Epal
XP_015768978.1_Adig
XP_001744777.1_Mbre
consensus/80%
B
Giantin
ATTHOG-1D4
C
wild type
Atthog -/-
No_SHH
Hi_SHH
100
75
75
75
100
37
SMO (post-ER)
SMO (ER)
FZD3
FZD4
LGR4
p38
D
Axin2 mRNA (AU)
0.00012
0.00008
0.00004
0.00000
- WNT3A
+ WNT3A
wild type
Atthog -/-
E
Id1 mRNA (AU)
0.06
0.04
0.02
0.00
- BMP4
+ BMP4
wild type
Atthog -/-
F
Streptavidin pull down
wild type_NoSHH
wild type_HiSHH
Atthog -/-_NoSHH
Input (10%)
Elution (100%)
time (h) 0 0.5 1 2 3 4 5 6
100
75
37
SMO
SMO*
p38
* lighter exposure

Figure S7. Related to Figures 6 and 7: Characterization of ATTHOG function in Hedgehog signaling. (A) Sequence alignment of members of the ATTHOG family of claudin-like tetraspanin proteins. The proteins are named by their GenBank identifiers (GIs), followed by their species abbreviations (see Methods) and colored per major lineages of holozoans. Predicted secondary structure is shown on the top, including the putative disulfide bridge in black. Conserved polar and charged residues that spatially cluster within the TM bundle (Figure 6B) are marked with blue circles at the bottom. The terminal cysteine predicted to be palmitoylated is highlighted with a pink circle. The alignment is colored based on 80% consensus with the following scheme: h (hydrophobic), l (aliphatic), and a (aromatic) are shaded yellow; p (polar) are shaded blue; positively charged and negatively charged residues are shaded green and red respectively; s (small) and t (tiny) are shaded light grey; b (big) is shaded dark gray. (B) ATTHOG staining in NIH/3T3 cells overlaps with staining of a marker of a Golgi marker (Giantin). (C) Immunoblotting was used to assess the abundance of SMO or three other members of the GPCR family (FZD3, FZD4, and LGR4) in wild type and *Atthog*$^{-/-}$ cells. (D and E) Endogenous transcriptional responses to WNT3A (D) and BMP4 (E) were measured in wild type and *Atthog*$^{-/-}$ NIH/3T3 cells using qRT-PCR for *Axin2* (target gene for WNT3A) and *Id1* (target gene for BMP4) (F) Immunoblotting was used to measure the abundance of biotinylated SMO at various times after labeling the SMO pool present at the plasma

membrane using a cell-impermeable biotinylation reagent (see methods). Scale bars, 10 μm in merged panels and 2 μm in zoomed displays.
